# Supplementary material for: A polycyclic scaffold identified by structure-based drug design effectively inhibits the human P2X7 receptor
Source: Nat Commun. 2025 Sep 15;16:8283. doi: 10.1038/s41467-025-62643-8 (PMC12436592; doi:10.1038/s41467-025-62643-8)
Supplement: Supplementary file 1 — Supplementary Information [file 41467_2025_62643_MOESM1_ESM.pdf]

A unique polycyclic scaffold identified by structure-based drug design effectively inhibits the human P2X7 receptor

Authors:

Adam C. Oken[a], Andreea L. Turcu[b,c], Eva Tzortzini[d], Kyriakos Georgiou[d], Jessica Nagel[e], Franka G. Westermann[e], Marta Barniol-Xicota[b,f], Jonas Seidler[g], Ga-Ram Kim[h], So-Deok Lee[h], Annette Nicke[g], Yong-Chul Kim[h], Christa E. Müller[e], Antonios Kolocouris[d], Santiago Vázquez[b,c], Steven E. Mansoor[a,i*]

Affiliations:

a. Department of Chemical Physiology & Biochemistry, Oregon Health & Science University, Portland, Oregon 97239, USA.
b. Laboratori de Química Farmacèutica, Facultat de Farmàcia i Ciències de l'Alimentació, Universitat de Barcelona, Av. Joan XXIII, 27-31, 08028 Barcelona, Spain.
c. Institute of Biomedicine of the University of Barcelona, IBUB, 08028 Barcelona, Spain.
d. Laboratory of Medicinal Chemistry, Section of Pharmaceutical Chemistry, Department of Pharmacy, National and Kapodistrian University of Athens, Panepistimiopolis-Zografou, 15771, Greece.
e. PharmaCenter Bonn & Pharmaceutical Institute, Pharmaceutical & Medicinal Chemistry, University of Bonn, 53121 Bonn, Germany.
f. Present address: Department of Medicine and Life Sciences, Biomedical Research Park (PRBB), Universitat Pompeu Fabra, 08003 Barcelona, Spain.
g. Walther Straub Institute of Pharmacology and Toxicology, Faculty of Medicine, Ludwig-Maximilians-Universität München, Munich, Germany.
h. School of Life Sciences, Gwangju Institute of Science and Technology, 123 Cheomdangwagi-ro, Buk-gu, Gwangju 61005, Republic of Korea.
i. Division of Cardiovascular Medicine, Knight Cardiovascular Institute, Oregon Health & Science University, Portland, Oregon 97239, USA.

* To whom correspondence should be addressed: mansoors@ohsu.edu

This PDF file includes:

Supplementary Figures 1 to 21
Supplementary Table 1
Synthesis and characterization methods
Detailed molecular dynamics simulations methods

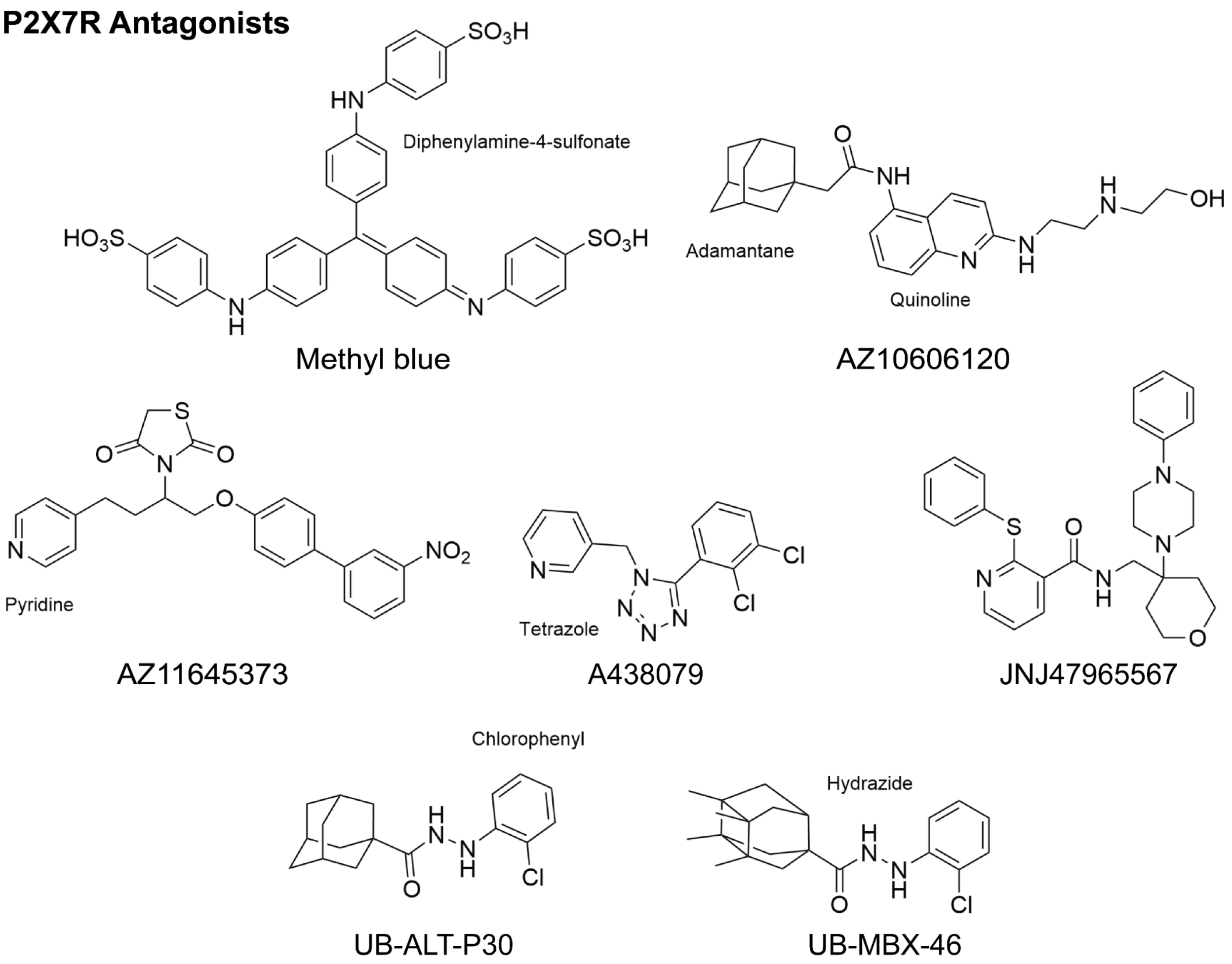

Supplementary Fig. 1: 2D chemical structures of allosteric P2X7R antagonists. The structural bases of antagonism for AZ10606120, A438079, JNJ47965567, and methyl blue have been previously described[1,2]. The synthesis and activity of UB-ALT-P30 was originally disclosed by Abbott[3]. In contrast, UB-MBX-46 is a new ligand scaffold with high potency and selectivity to the hP2X7R.

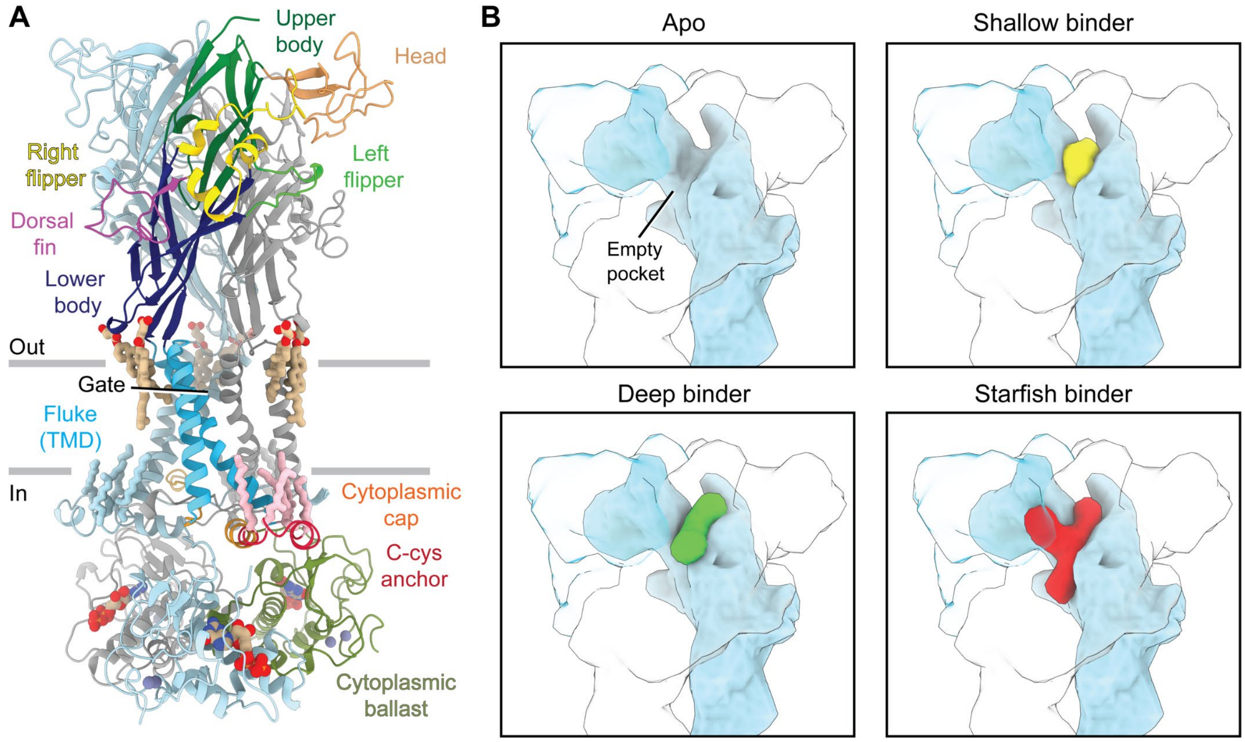

Supplementary Fig. 2: Domain architecture and allosteric binding modes of the P2X7R. (**A**) Ribbon representation of the hP2X7R in the apo closed state with one protomer colored by domain nomenclature and the other two colored in light blue and grey[4,5]. (**B**) Cartoon representation of the P2X7R highlighting three allosteric antagonist binding modes defined from structures of antagonists bound to the rP2X7R ortholog, with one protomer shown in light blue and the other two in transparent blue and transparent light grey[2]. The apo receptor with an empty allosteric pocket contrasts allosteric antagonists with a shallow binding mode (yellow), a deep binding mode (green), and a starfish binding mode (red)[2]. Each mode of binding has distinct functional properties[2].

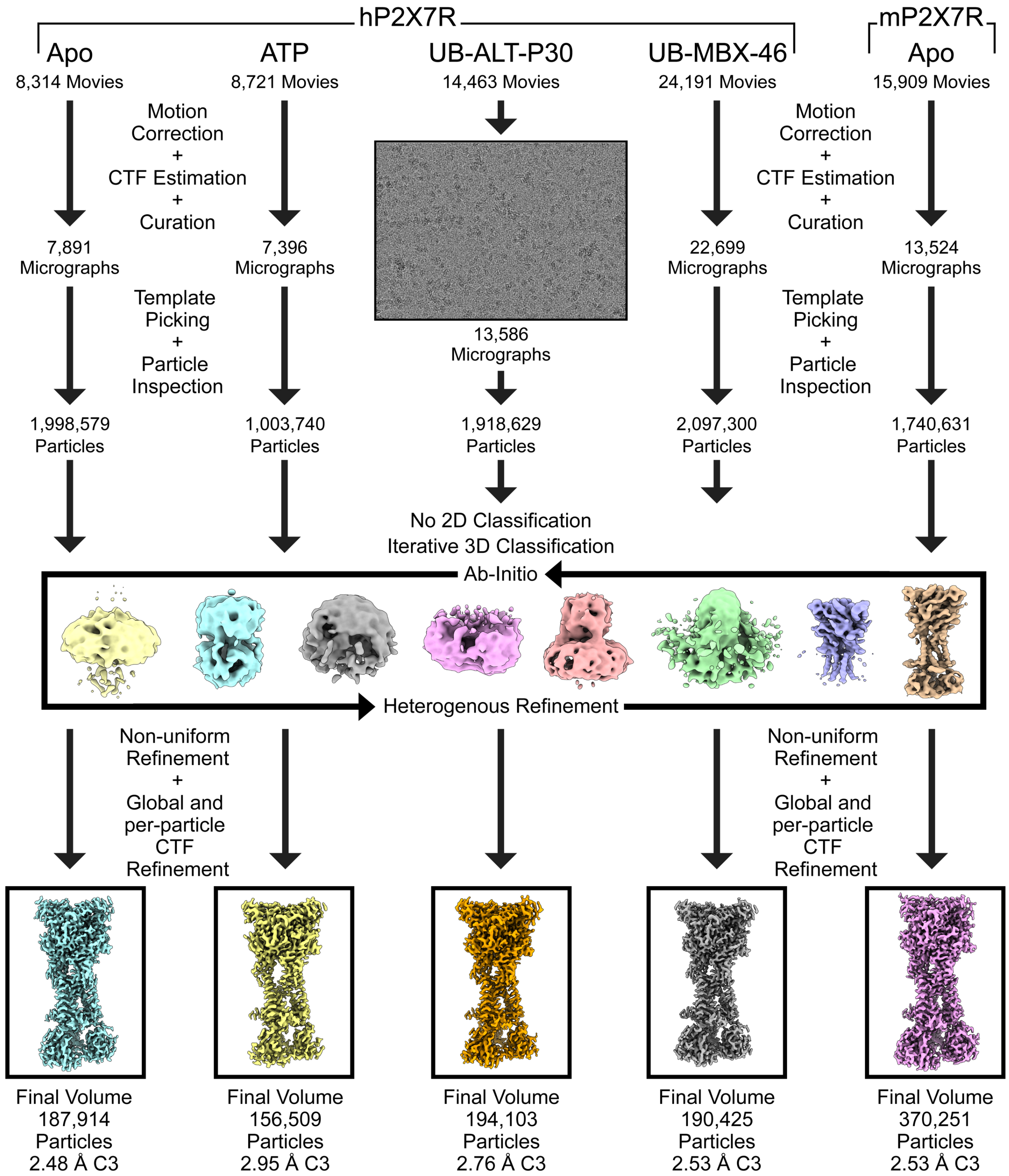

Supplementary Fig. 3: Cryo-EM processing pipeline for P2X7R reconstructions. Following data acquisition, movies were binned to the physical pixel size during motion correction and patch CTF correction was performed in cryoSPARC[6]. Next, micrographs were culled, and particles were picked using 2D templates generated from a low-resolution 3D reconstruction. Initial particle picks were inspected, extracted, and sent directly to iterative 3D classification in cryoSPARC using ab initio and heterogeneous refinement jobs. After classification into a culled particle stack, the final particles were re-extracted at the physical pixel size for non-uniform

refinement performed with 3-fold (C3) symmetry as well as global and per-particle CTF refinements[7].

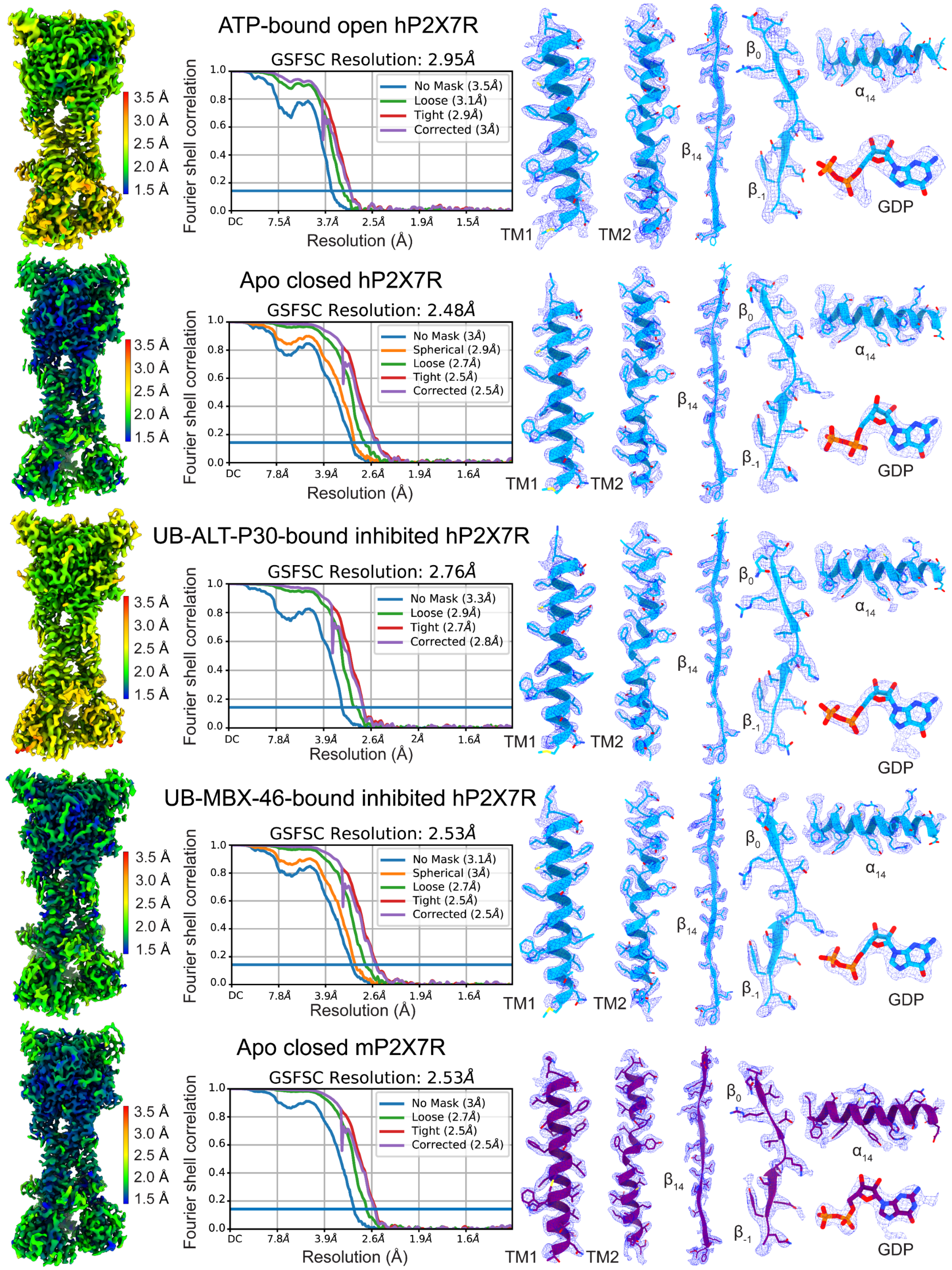

Supplementary Fig. 4: FSC and local resolution plots for P2X7R reconstructions.

Resolution stated is at an FSC = 0.143. All local resolution plots range between 1.5 Å (blue) and 3.5 Å (red). Representative regions (TM1, TM2, β_{-1}, β_0, β_{14}, α_{14}, and GDP) from the structures of the ATP-bound open hP2X7R, apo closed hP2X7R, UB-ALT-P30-bound inhibited hP2X7R, UB-MBX-46-bound inhibited hP2X7R, and apo closed mP2X7R shown within their respective cryo-EM densities (blue mesh) highlighting the strong map-to-model fits. Models for the hP2X7R are colored blue and the model for the mP2X7R is colored purple.

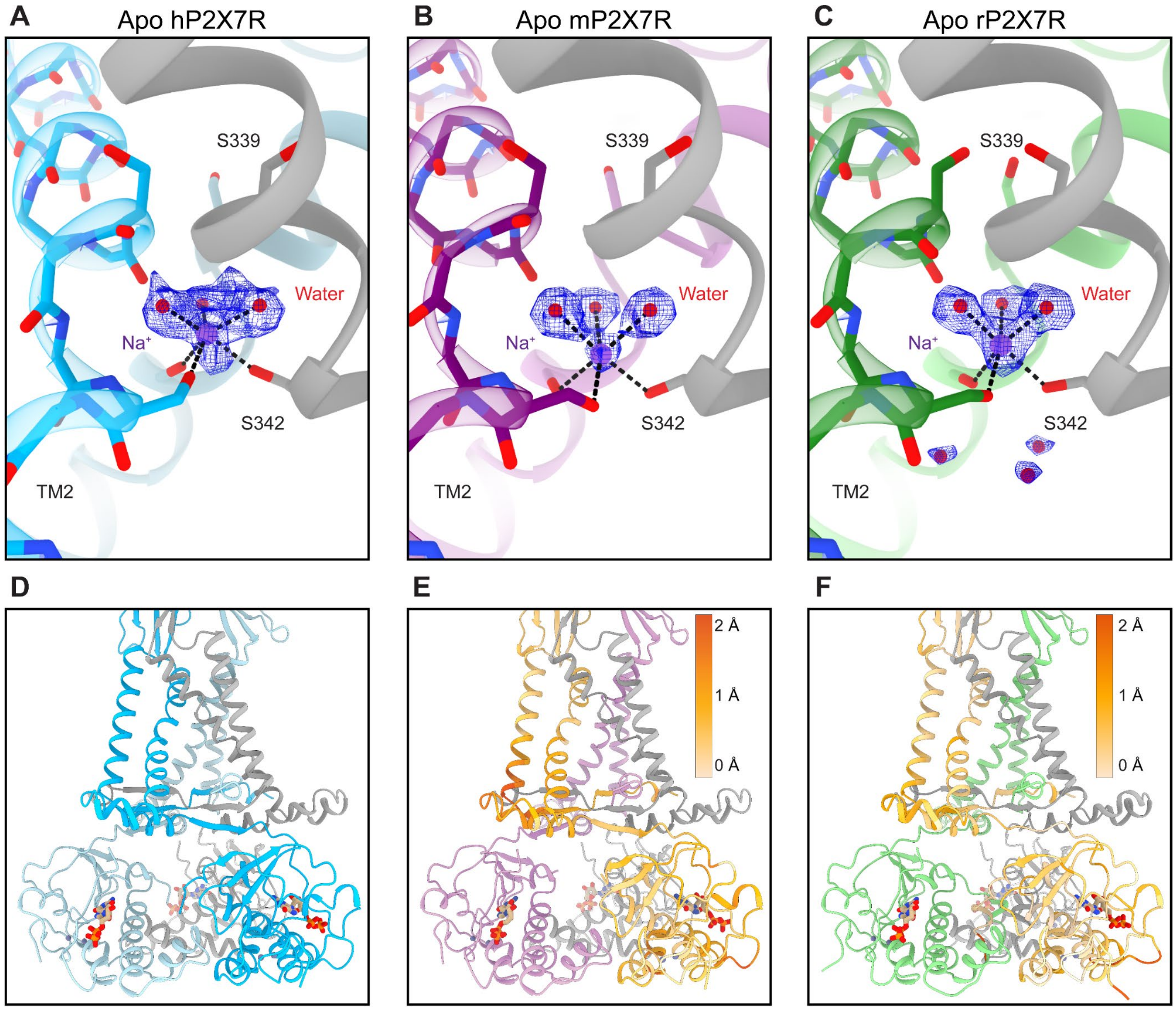

Supplementary Fig. 5: The pore architecture and cytoplasmic domain of P2X7Rs are conserved across human, mouse, and rat orthologs. (**A-C**) Ribbon representation of the hP2X7R (shades of blue and grey), the mP2X7R (shades of purple and grey), and the rP2X7R (shades of green and grey, PDB code 8TR5) in the apo closed state conformation highlighting the coordination of a partially hydrated Na^+ ion (purple sphere) directly above the closed gate with the cryo-EM density shown in blue mesh[8]. Across all three ortholog structures, the partially hydrated Na^+ ion is coordinated by three symmetry-related waters above (red spheres) and the sidechain hydroxyl of S342 from each protomer below[8]. The identity of the partially hydrated Na^+ ion was previously confirmed, and the same coordination is now observed in human and mouse orthologs for the first time[2,8]. (**D-F**) Ribbon representation of the transmembrane and cytoplasmic domains of human, mouse, and rat P2X7Rs in the apo closed state conformation are similar and maintain the same ligands (one GDP molecule and two zinc ions per protomer). (**D-F**) View highlighting the cytoplasmic domains of the hP2X7R (shades of blue and grey), the mP2X7R (shades of purple and grey), and the rP2X7R (shades of green and grey, PDB code 8TR5) in the apo closed state[8]. (**E and F**) Same view as **D** with one protomer of the mP2X7R (**E**) or the rP2X7R (**F**) colored in shades of orange by RMSD as compared to the human

ortholog, highlighting the similarity between ortholog structures. A light tan color represents an RMSD of 0 Å while a dark orange indicates a 2 Å or greater change.

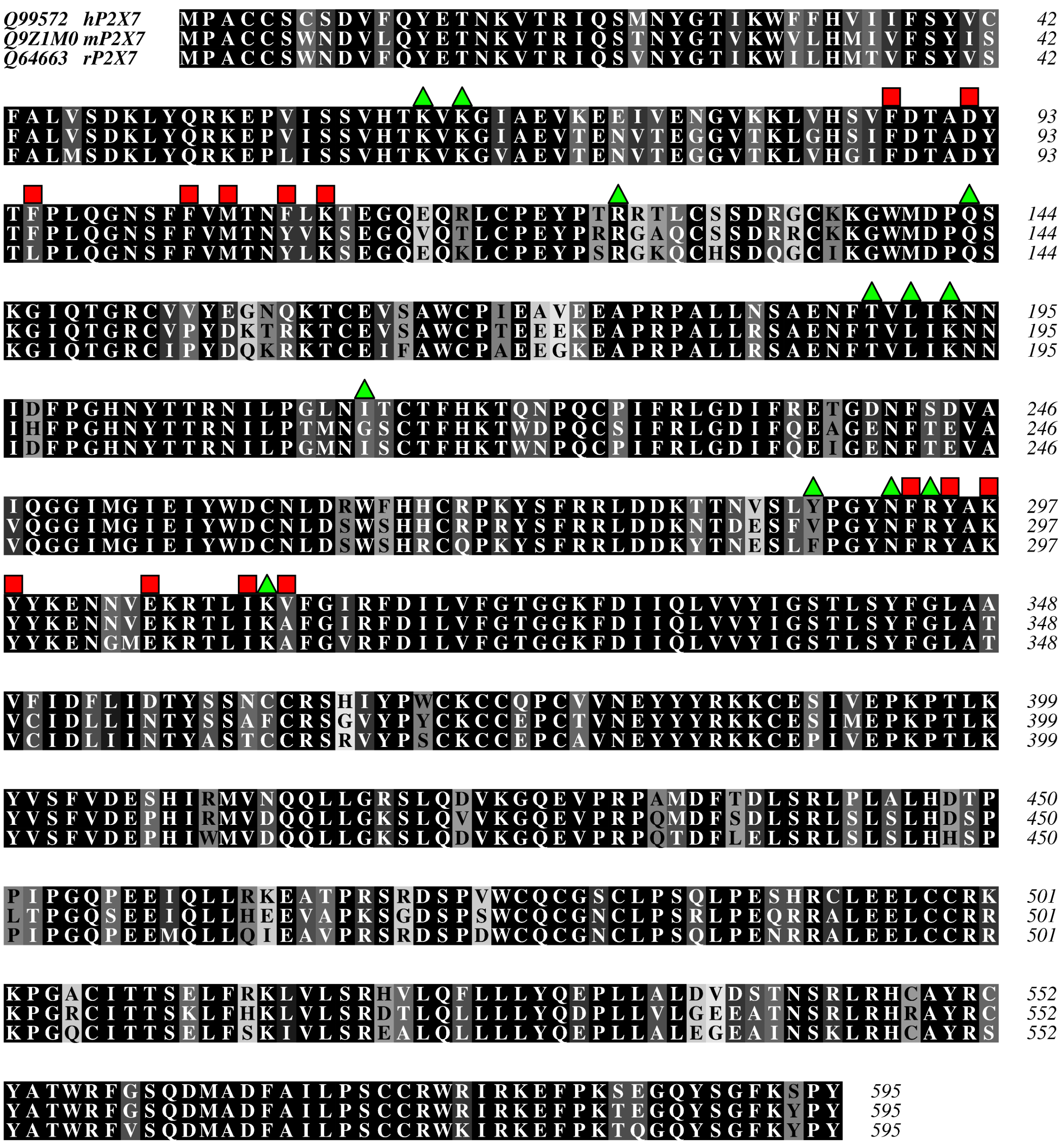

Supplementary Fig. 6: Sequence alignment of human, mouse, and rat P2X7Rs. Protein sequence alignment of human, mouse, and rat P2X7Rs colored by level of sequence conservation as calculated by Alscript with black denoting full sequence conservation and white denoting no sequence conservation[9]. Green triangles above the alignment indicate the residues that coordinate ATP. Red squares above the alignment indicate the residues that are located within the classical allosteric ligand-binding site. The sequence alignment was generated in Clustal Omega and visualized in Aline[10,11]. Sequences were obtained from UniProt with accession numbers Q99572, Q9Z1M0, and Q64663 for the hP2X7R, the mP2X7R, and the rP2X7R, respectively.

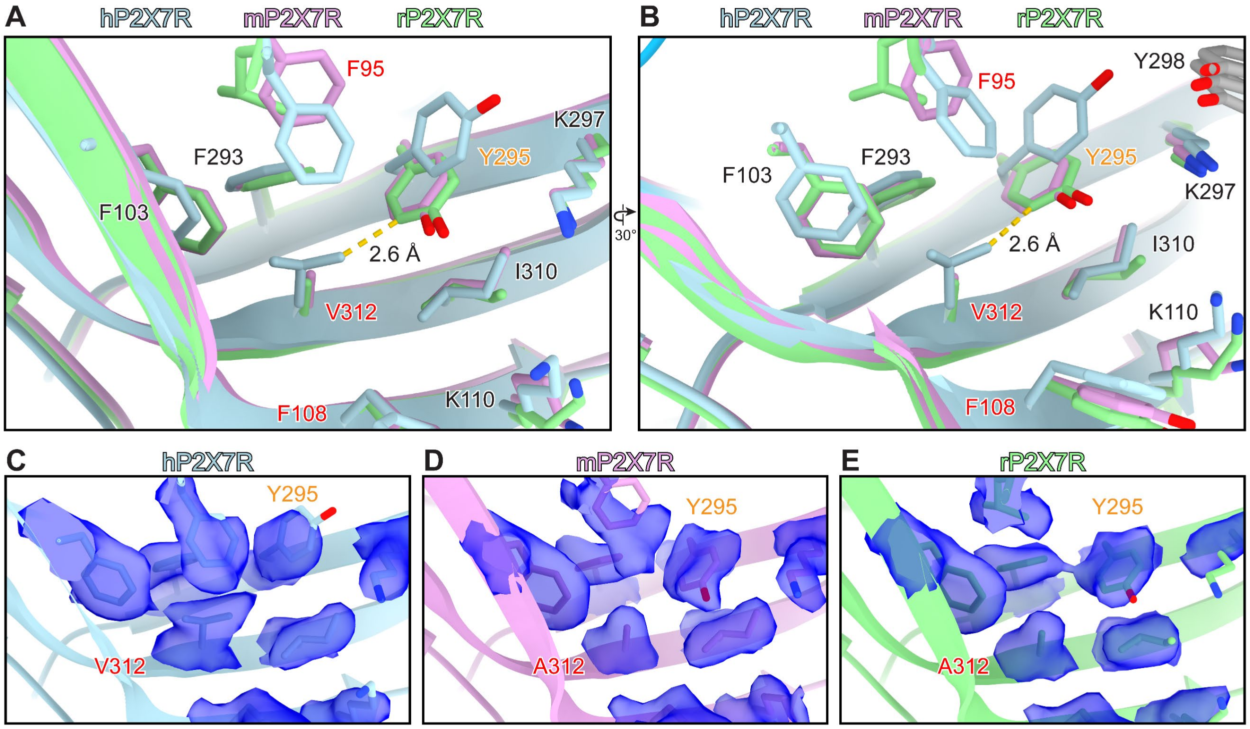

Supplementary Fig. 7: Residue V312 in the hP2X7R forces Y295 to adopt a different rotameric conformation, changing the shape of the classical allosteric pocket in the human ortholog. (**A**) Magnified view of the classical allosteric ligand-binding site focusing on residues 312 and 295 between human (light blue), mouse (light purple), and rat (light green) P2X7Rs. Residues 95, 108, and 312 (all labeled in red) have different residue identities between the three orthologs. The larger V312 in hP2X7Rs (A312 in mP2X7Rs and rP2X7Rs) forces the neighboring residue Y295 (orange) to adopt an alternative rotameric conformation. The shortest distance between the sidechain of V312 in the hP2X7R to the sidechain of Y295 in the mP2X7R or rP2X7R is only ~2.6 Å, which would create a steric clash. Thus, Y295 in the hP2X7R is forced into a distinct rotameric conformation. (**B**) Small rotation (30°) from panel **A** highlighting a slightly different view of residues V312 and Y295 in the hP2X7R. (**C-E**) Same view as panel **A** highlighting the good fit of sidechains within cryo-EM density (blue transparent surface) for each P2X7R ortholog.

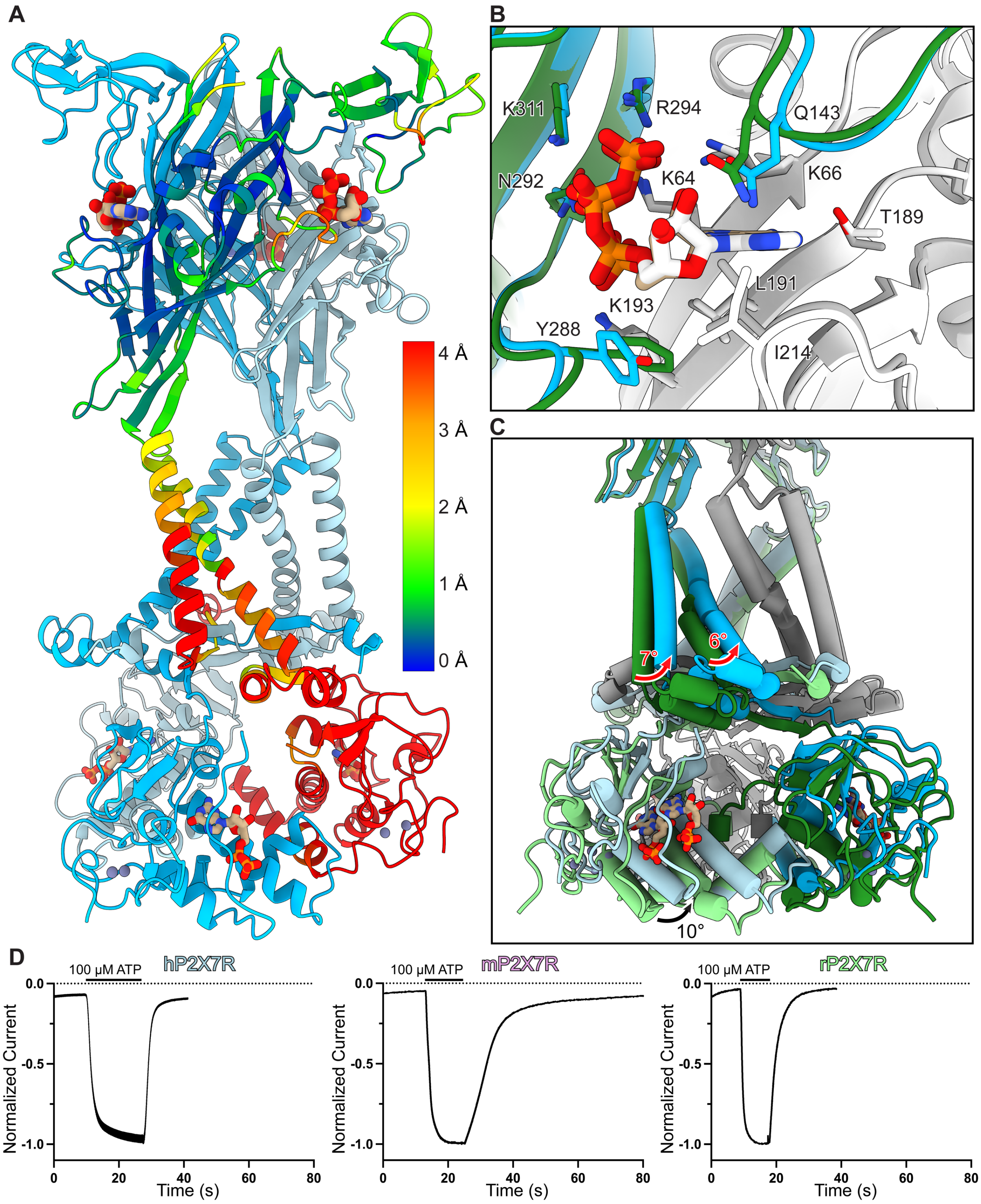

Supplementary Fig. 8: Structural differences between the rat and human P2X7Rs in the ATP-bound open state conformation. (**A**) Ribbon representation of the hP2X7R in the ATP-bound open state conformation (shades of blue) with one protomer colored by RMSD according to the structural differences between human and rat ortholog structures (PDB code: 8TR5)[8]. Residues colored in blue signify little change with an RMSD of 0 Å while residues colored in red

signify a larger change with an RMSD of >4 Å. Overall, the extracellular domains of the rP2X7R and the hP2X7R structures in the ATP-bound open state are similar with small RMSDs, while the transmembrane and cytoplasmic domains are different with larger RMSDs. (**B**) Same view as Fig. 2E highlighting the structural differences in the orthosteric ATP-binding pocket between the ATP-bound open states of the rP2X7R (green and white with the carbon atoms of ATP in white) and the hP2X7R (blue and grey with the carbon atoms of ATP in tan). (**C**) Magnified view of the transmembrane and cytoplasmic domains of the rP2X7R and the hP2X7R in the ATP-bound open state conformation highlighting the 7º and 6º rotations of TM1 and TM2, respectively, ultimately resulting in a 10º rotation of the cytoplasmic ballast between rat and human receptor orthologs. These rotations correspond to lateral displacements of 5.2 Å (distance between Cα carbons of residue 24) at the start of TM1 and 4.8 Å (distance between Cα carbons of residue 358) at the end of TM2, respectively. (**D**) Representative TEVC traces of wild-type hP2X7R (left), mP2X7R (middle), and rP2X7R (right) after facilitation, highlighting activation by 100 μM ATP, no desensitization, and deactivation following the removal of ATP.

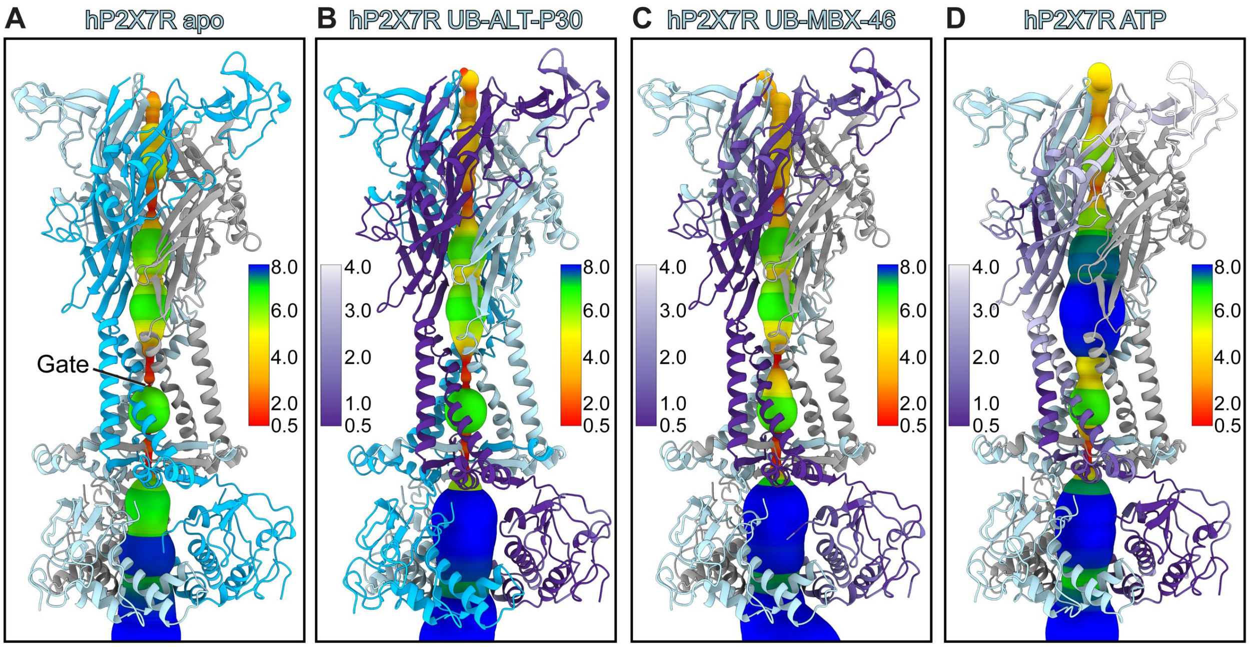

Supplementary Fig. 9: Comparison of the pore and general architecture between structures of the hP2X7R. (**A-D**) Ribbon representation of the hP2X7R (shades of blue and gray) in (**A**) the apo closed state, (**B**) the UB-ALT-P30-bound inhibited state, (**C**) the UB-MBX-46-bound inhibited state, and (**D**) the ATP-bound open state. The ion permeation pathways of the hP2X7R in each state were generated by MOLEonline with different colors representing different pore radii (color key shown on the right edge of the panel)[12]. The constriction gates, formed by Ser342 from each protomer, in the apo closed and antagonist-bound inhibited states are too small to pass dehydrated Na^+ ions while the gate in the ATP-open state is large enough to pass partially hydrated Na^+ ions[13]. (**B-D**) One protomer of the hP2X7R in different ligand-bound states is colored by RMSD (shades of purple) according to the structural variations compared to the hP2X7R in the apo closed state. Residues colored in dark purple signify little change with an RMSD of 0.5 Å while residues colored in pale purple signify a larger change with an RMSD of >4 Å (color key shown on the left edge of the panel). (**B-D**) Overall, the structures of the hP2X7R in the apo closed and antagonist-bound inhibited states are very similar. (**D**) As expected, the ATP-bound open state of the hP2X7R is quite different than the apo closed state, especially in the extracellular and transmembrane domains. The structural movements involved in P2XR activation have been described previously[14,15].

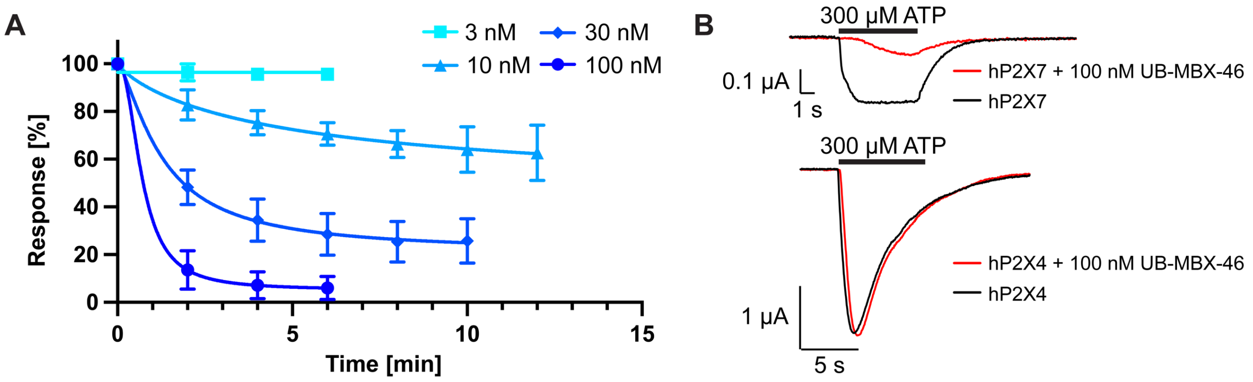

C

Antagonist	UB-ALT-P30	UB-MBX-46
IC_{50} (M)	1.5×10^{-8} [1.2×10^{-8} - 2.0×10^{-8}]	3.8×10^{-9} [3.6×10^{-9} - 4.0×10^{-9}] 1.8×10^{-10}*
K_i (M)	2.8×10^{-8}	1.03×10^{-8}
k_{on} (nM x min)$^{-1}$	0.0105 [0.0076 - 0.0133]	0.0065 [0.0058 - 0.0073]
k_{off} (min)$^{-1}$	0.29 [0.12 - 0.46]	0.07 [0.03 - 0.11]
k_{off} (min)$^{-1}$ (based on IC_{50})	0.15	0.025 0.001*

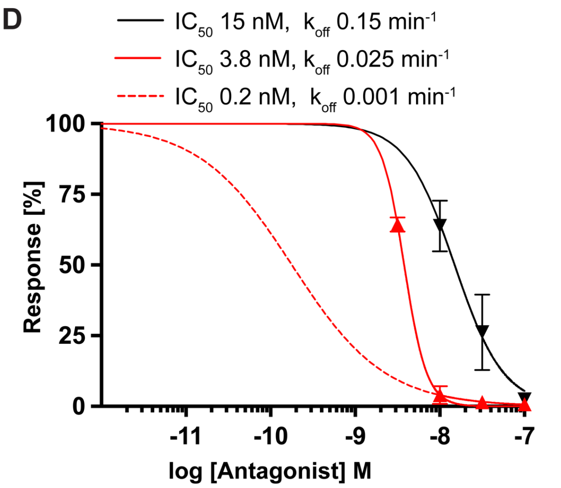

Supplementary Fig. 10: Kinetics of ligands binding to the hP2X7R measured by TEVC. (**A**) Normalized current response to 5s-pulses of 300 µM ATP over time with the indicated concentrations of UB-ALT-P30 continuously perfused. All data represented as mean ± S.D. with n=3, 7, 7, and 7 for the experiments performed at 3 nM, 10 nM, 30 nM, and 100 nM concentrations of UB-ALT-P30, respectively. (**B**) Representative current traces for the activation of the hP2X7R (top, black) and the hP2X4R (bottom, black) by 300 µM ATP with or without the co-application of 100 nM UB-MBX-46 (red traces), highlighting strong antagonism of the hP2X7R and virtually no antagonism of the hP2X4R. (**C**) On- and off-rate constants and K_i values for UB-ALT-P30 and UB-MBX-46 at the hP2X7R. Observed on-rates k_{obs} were determined by fitting the data (Fig. 4H, Supplementary Fig. 10A) to the function: *% response = (100-Plateau)*exp(-*k_{obs}**time)+Plateau* and plotted against the antagonist concentrations to obtain k_{off} according to $k_{obs}=k_{on}$*F+k_{off}. Assuming a (simplified) 1:1 binding model, off-rate constant k_{off} and K_i values were estimated using the previous formulas as well as $K_i=k_{off}/k_{on}$. While the K_i value of UB-ALT-P30 (28 nM) agreed very well with both the IC_{50} values determined by Ca^{2+} imaging (18 nM, Fig. 3B) and the binding equilibrium reached at each antagonist concentration (15 nM, panel **D**), the graphically determined k_{off} and resulting K_i values of UB-MBX-46 cannot reflect an accurate value due to the nearly negligible dissociation on the timescale of the experiment. If k_{off} was estimated based on the IC_{50} value (IC_{50} = k_{off}/k_{on} panel **D**), a more accurate k_{off} value can be obtained. Numbers in square brackets are 95% Confidence Intervals. *values obtained from an extrapolated dose inhibition curve from **D**. (**D**) The plateau values, representing the binding equilibrium, were used to generate dose inhibition

curves. The dotted line represents an extrapolated curve without the 3 nM plateau value, which might be overestimated due to technical limitations at low agonist concentrations. The respective k_{off} value represents the irreversible binding more accurately. All data are represented as mean ± S.D. from 3 or 4 measurements.

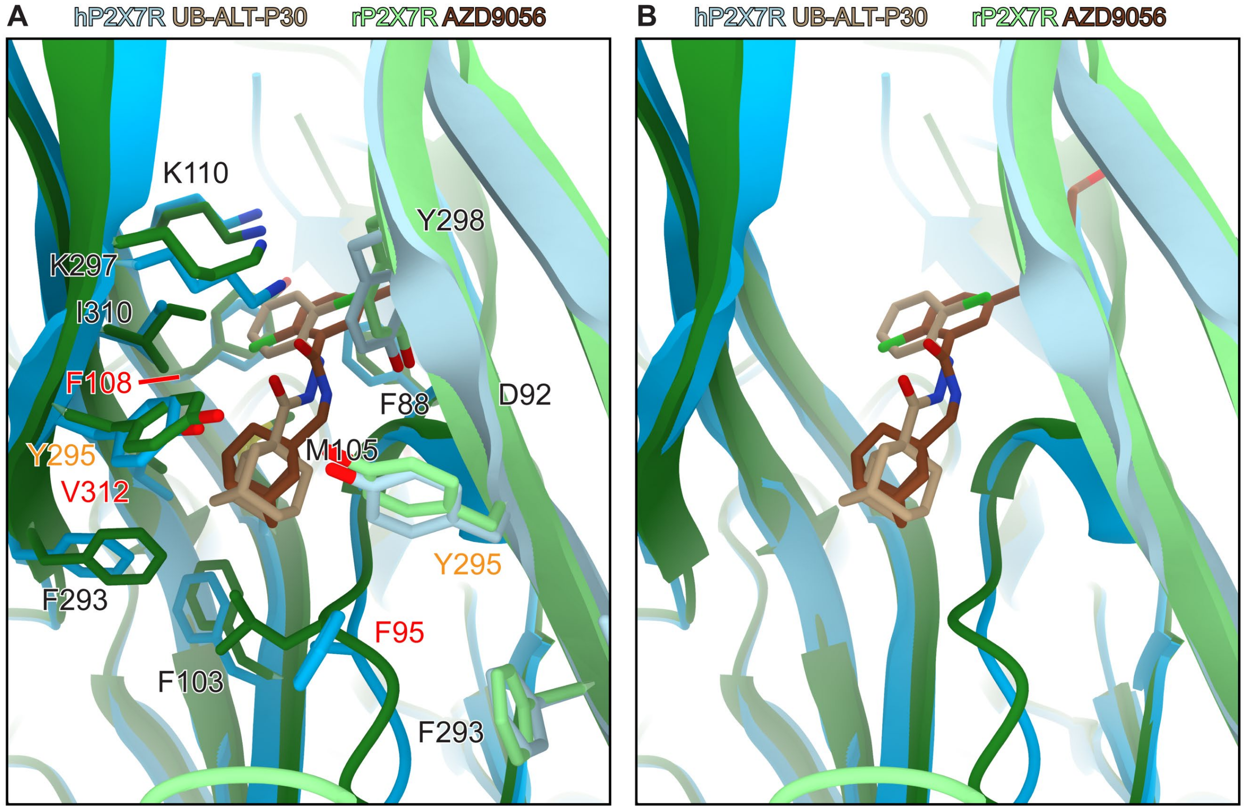

Supplementary Fig. 11: Comparison of adamantane-containing antagonists bound to the classical allosteric pocket of rat and human P2X7Rs. (**A**) Ribbon representation of UB-ALT-P30 bound to the hP2X7R (shades of blue, ligand in tan) compared to AZD9056 bound to the rP2X7R (shades of green, ligand in brown, PDB code: 8TR8), highlighting the residue interactions with UB-ALT-P30 or AZD9056 within the classical allosteric pocket[2]. Residues 95, 108, and 312 (red) as well as Y295 (orange) are the key ortholog-specific differences in the classical allosteric pockets. (**B**) Same view as panel **A** without sidechains, highlighting the different poses of UB-ALT-P30 and AZD9056 within the classical allosteric pocket of hP2X7R and rP2X7R, respectively.

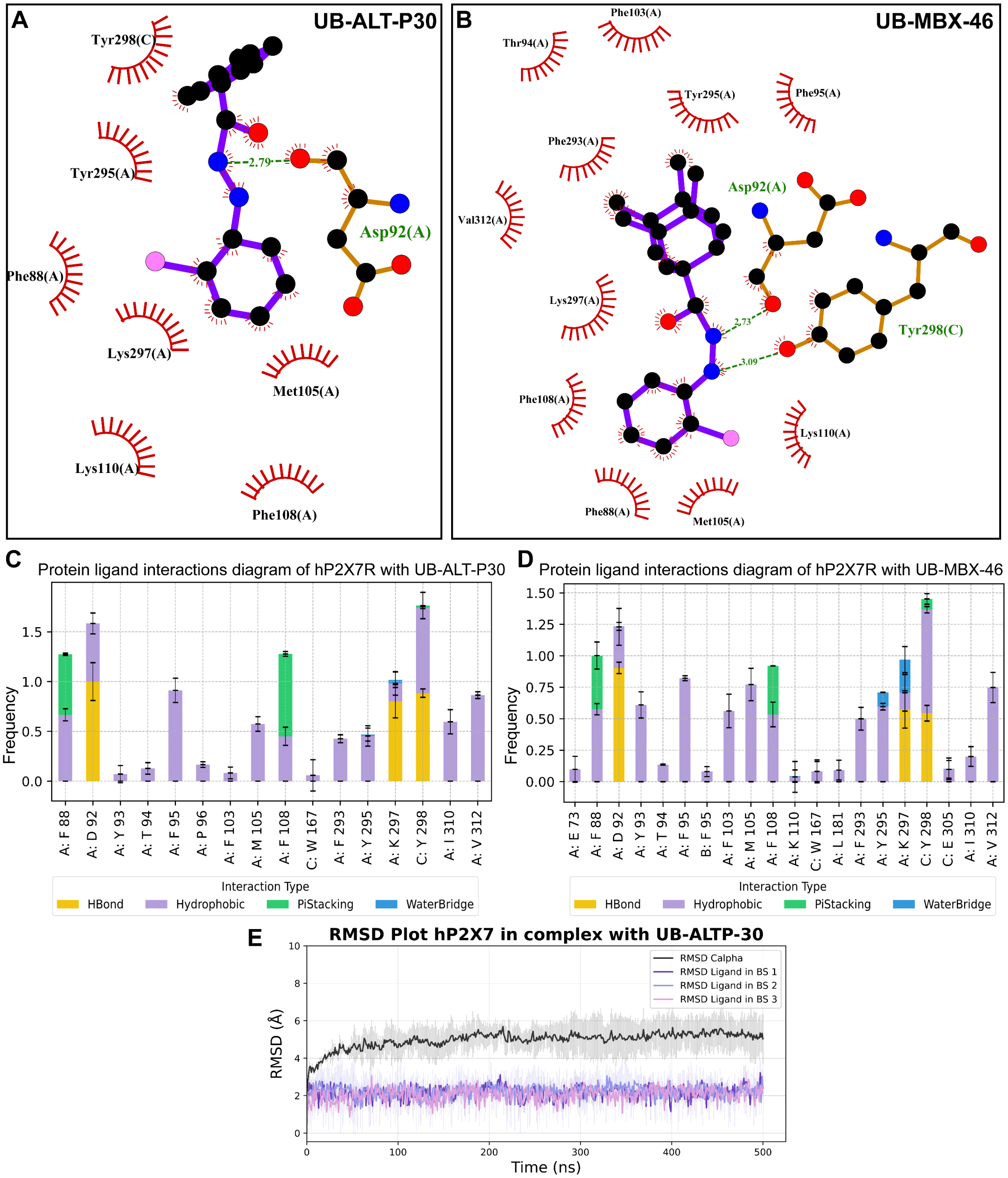

Supplementary Fig. 12: Receptor-ligand interactions for UB-ALT-P30 and UB-MBX-46 bound to the classical allosteric pocket of the hP2X7R. (**A and B**) LigPlot diagrams for UB-ALT-P30 (**A**) and UB-MBX-46 (**B**) bound to the classical allosteric pocket of the hP2X7R from cryo-EM structures[16]. These diagrams highlight the interactions between the ligand and the receptor. (**C and D**) Results from 500 ns simulations of full-length wild-type hP2X7R in complex with UB-ALT-P30 (**C**) or UB-MBX-46 (**D**), respectively. Amino acid-ligand frequency interactions

are depicted with bar plots. Each color within the graph corresponds to a different interaction: hydrogen bonding interactions are shown with yellow bars; hydrophobic interactions are shown with magenta bars; cation-π interactions are shown with green bars; water bridges with blue bars. Bars are plotted only for residues with interaction frequencies ≥ 0.2 and their standard deviations across two replicas are depicted. (**E**) RMSD plots of Cα carbons for the protein and of the heavy atoms for the ligand UB-ALT-P30; statistics from three replicas are described with the shaded plots. This plot confirms the protein reached equilibrium in the simulations for each of the three symmetry-related binding sites (BS) in the receptor.

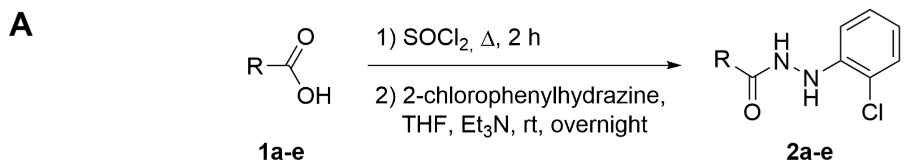

a, R = Adamant-1-yl; **b**, R = 3,5-dimethyladamant-1-yl; **c**, R = 3,5,7-trimethyladamant-1-yl; **d** = 1-cubyl, **e** = 3,4,8,9-tetramethyltetracyclo[4.4.0.$0^{3,9}$.$0^{4,8}$]dec-1-yl.

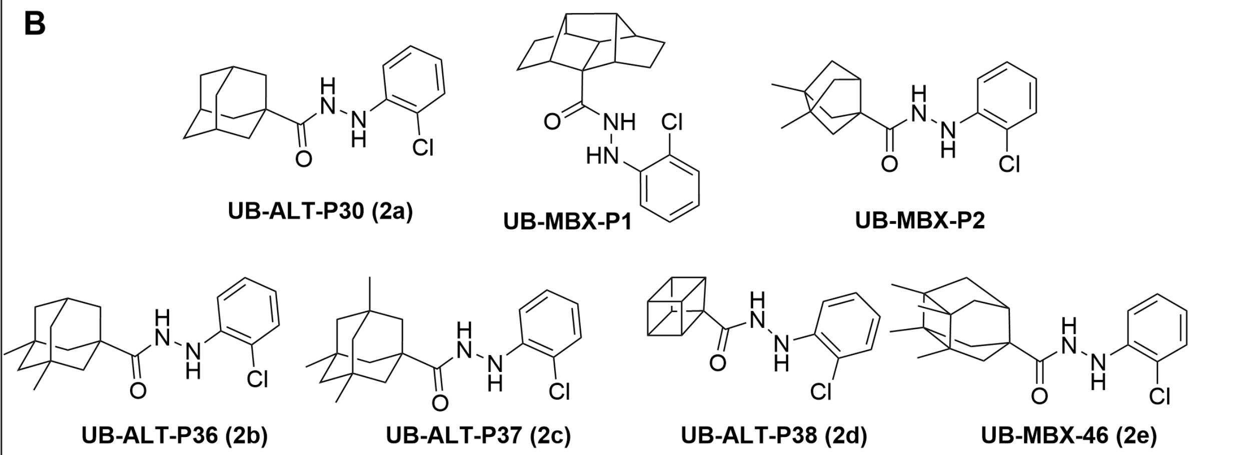

Supplementary Fig. 13: (A) Synthetic route for the new acyl hydrazines. (B) Chemical structures of known P2X7R antagonists (UB-ALT-P30, UB-MBX-P1, UB-MBX-P2) and previously uncharacterized polycyclic analogs (UB-ALT-P36, UB-ALT-P37, UB-ALT-P38 and UB-MBX-46)[3,17].

^{1}H NMR (400 MHz, $CDCl_3$)

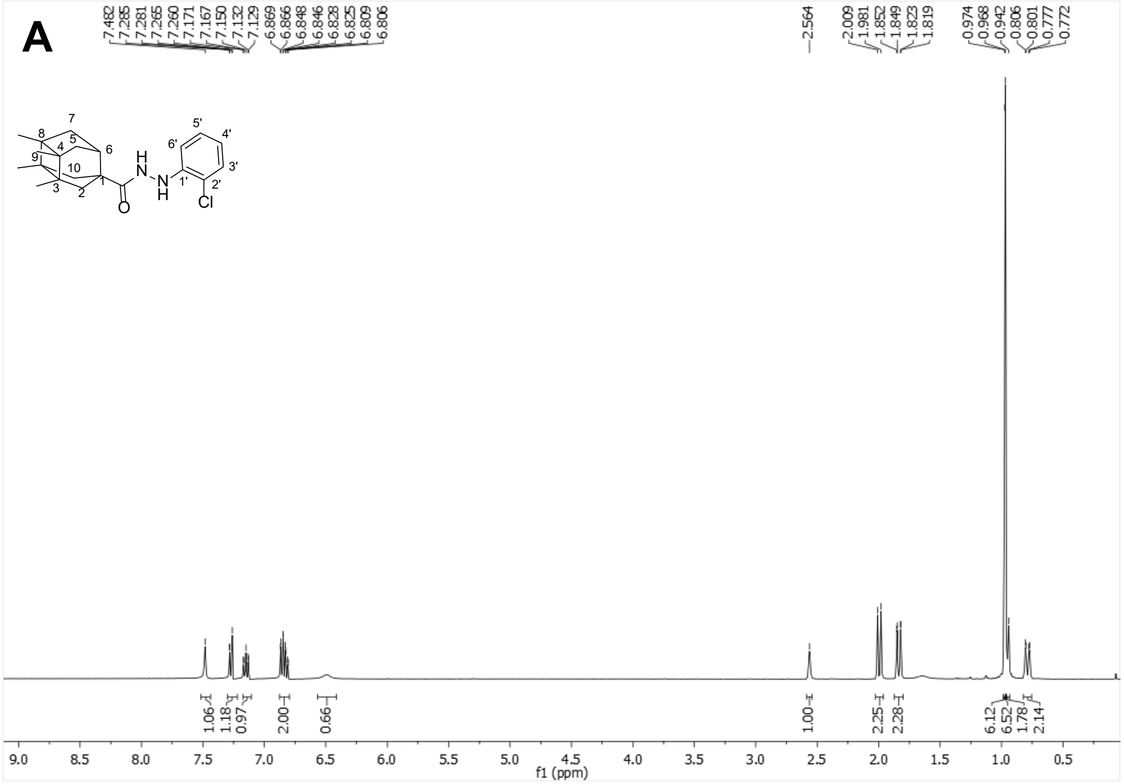

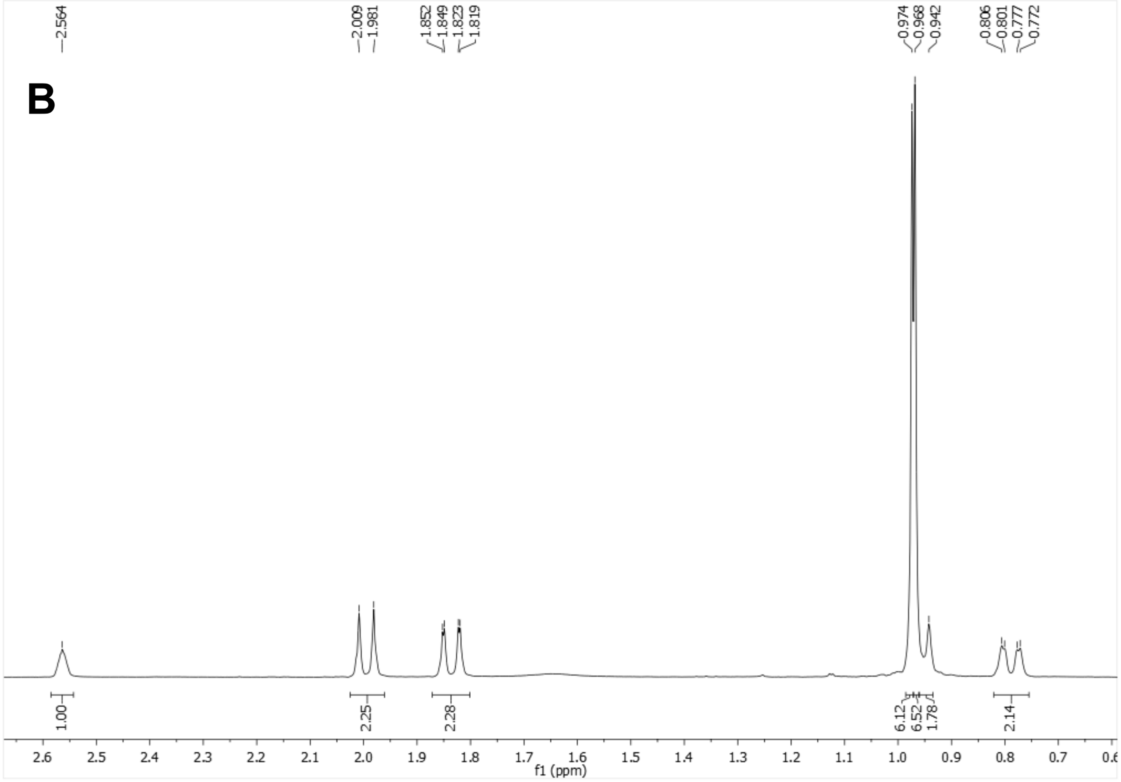

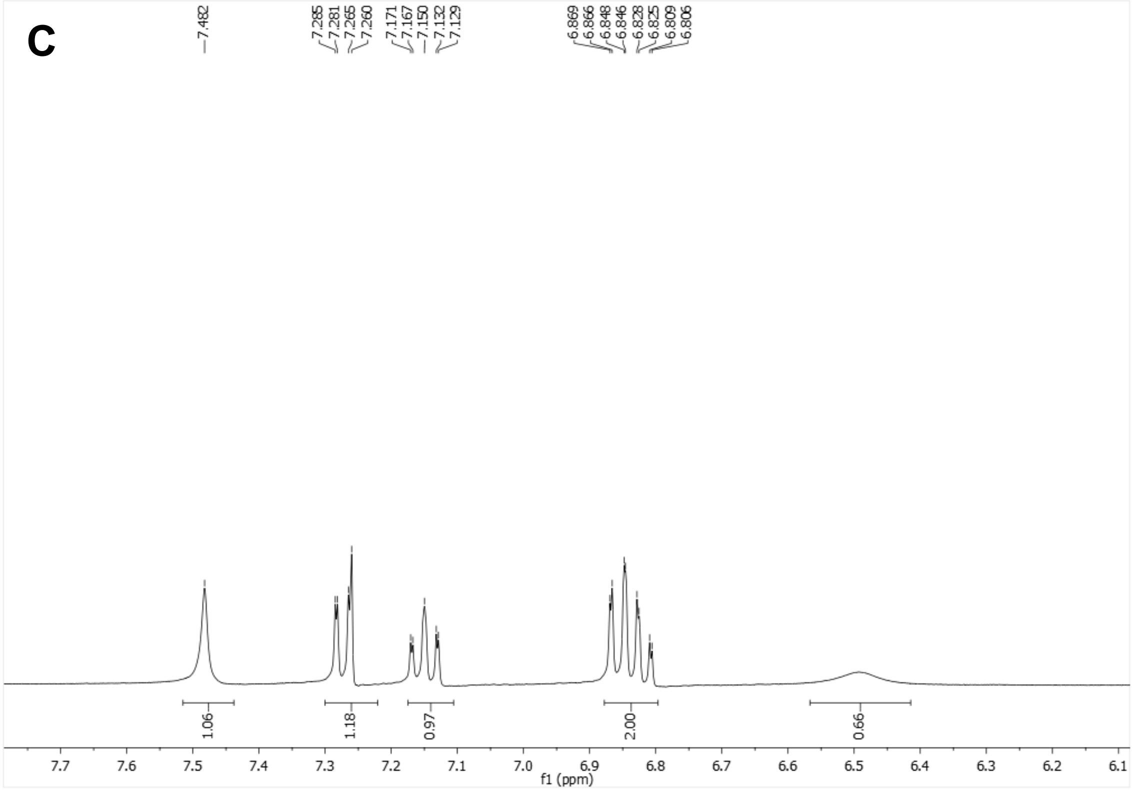

^{13}C-NMR (100.6 MHz, $CDCl_3$)

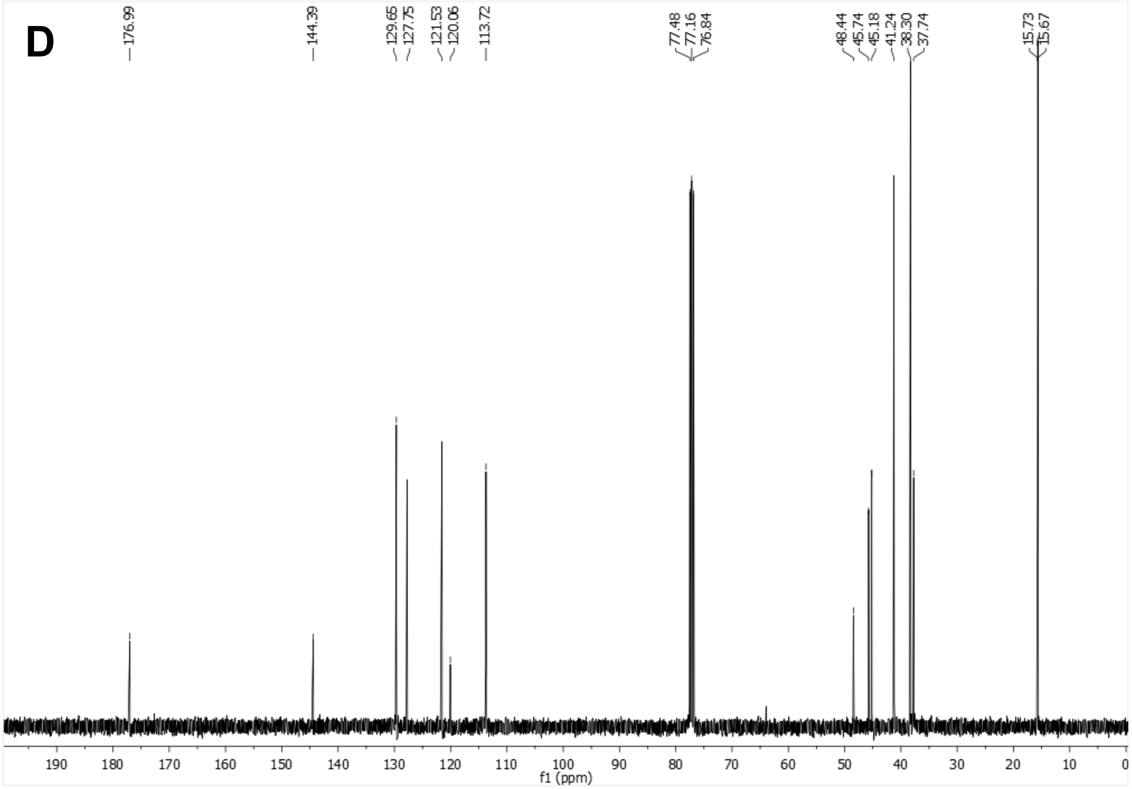

E

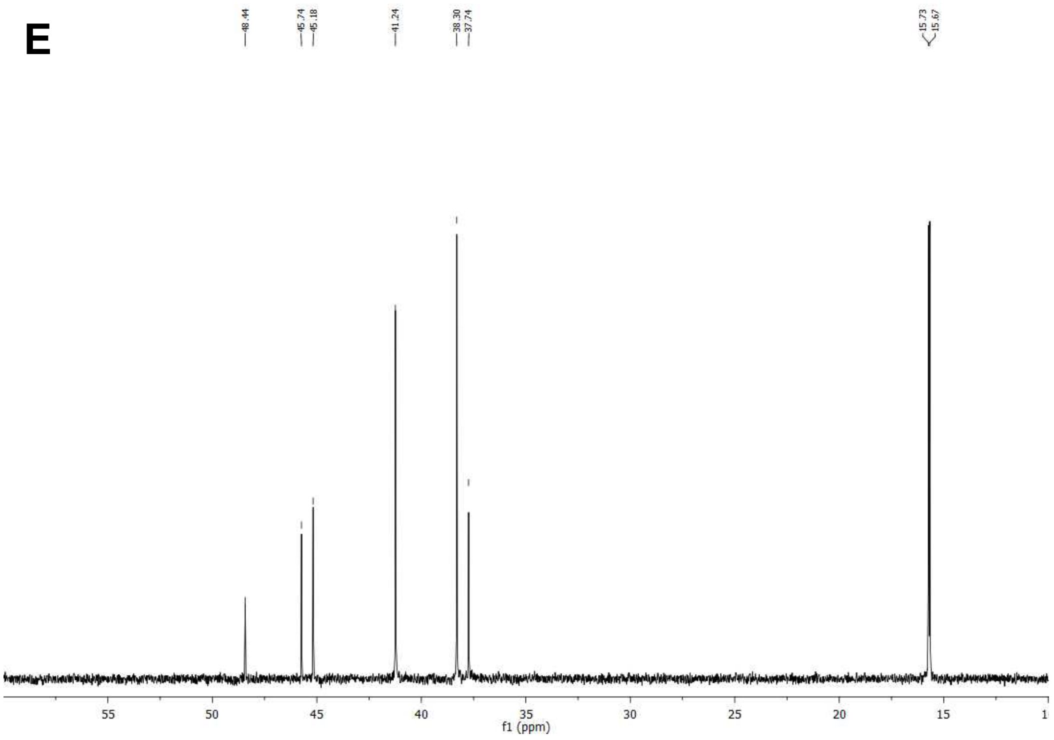
48.44
45.74
45.18
41.24
38.30
37.74
15.73
15.67
55
50
45
40
35
30
25
20
15
f1 (ppm)

F

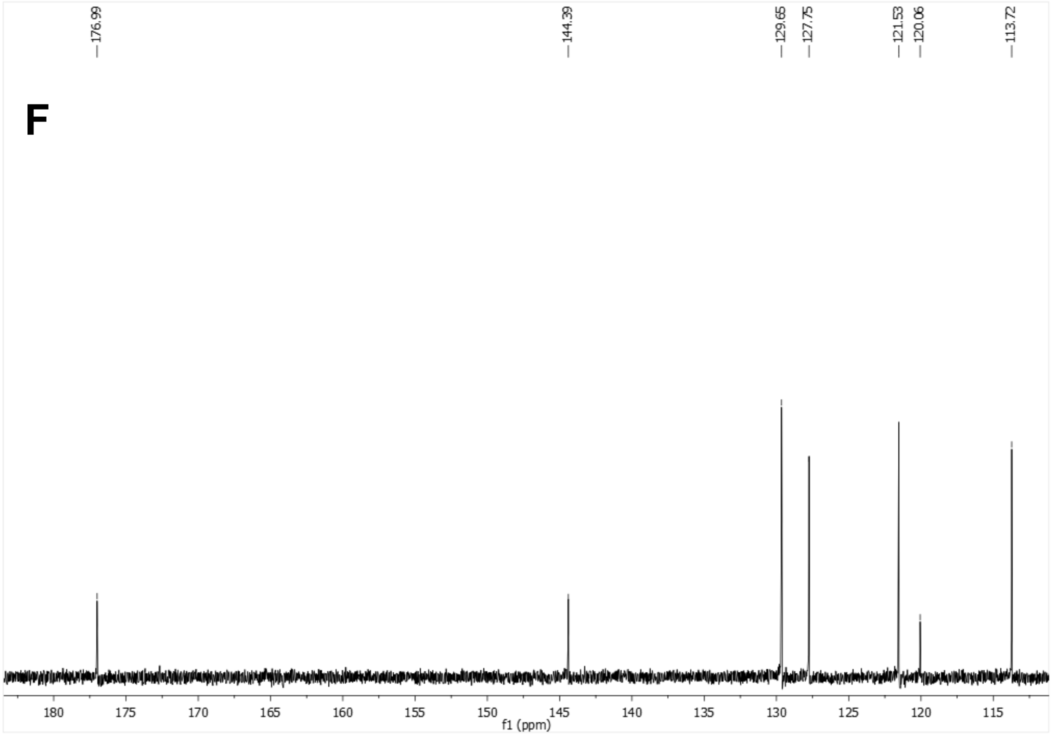
176.99
144.39
129.65
127.75
121.53
120.06
113.72
180
175
170
165
160
155
150
145
140
135
130
125
120
115
f1 (ppm)

HSQC ($CDCl_3$)

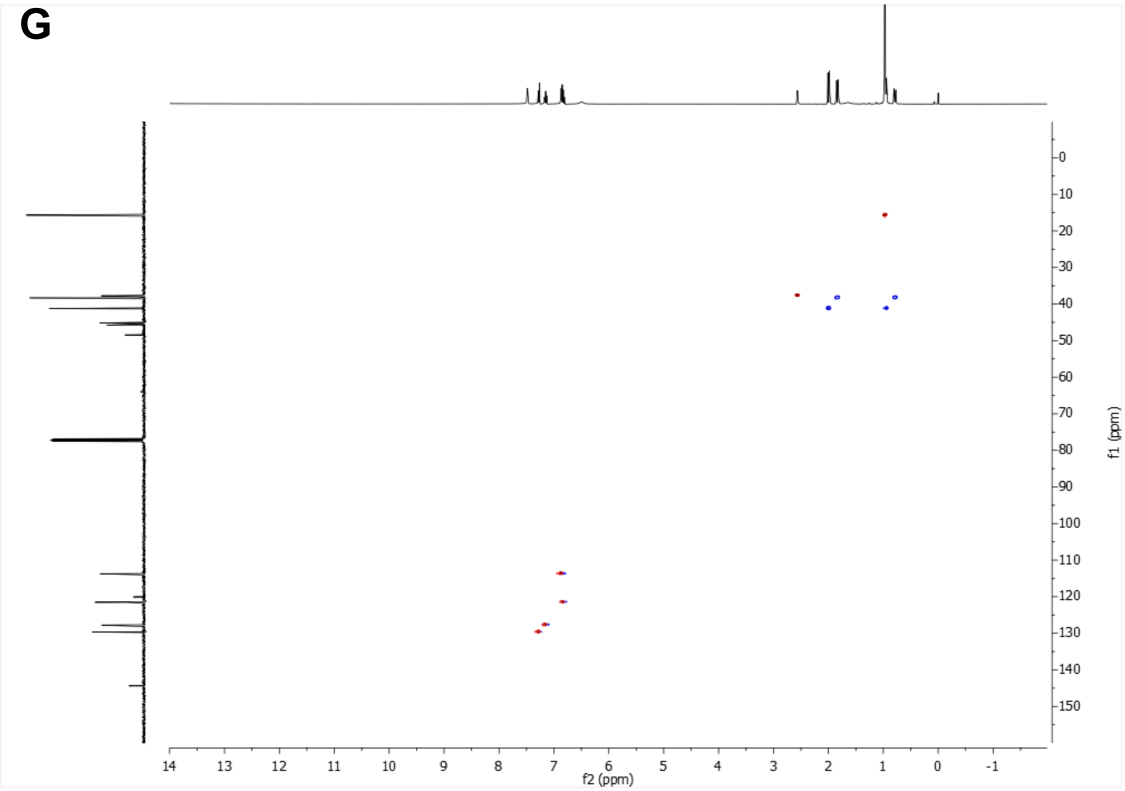

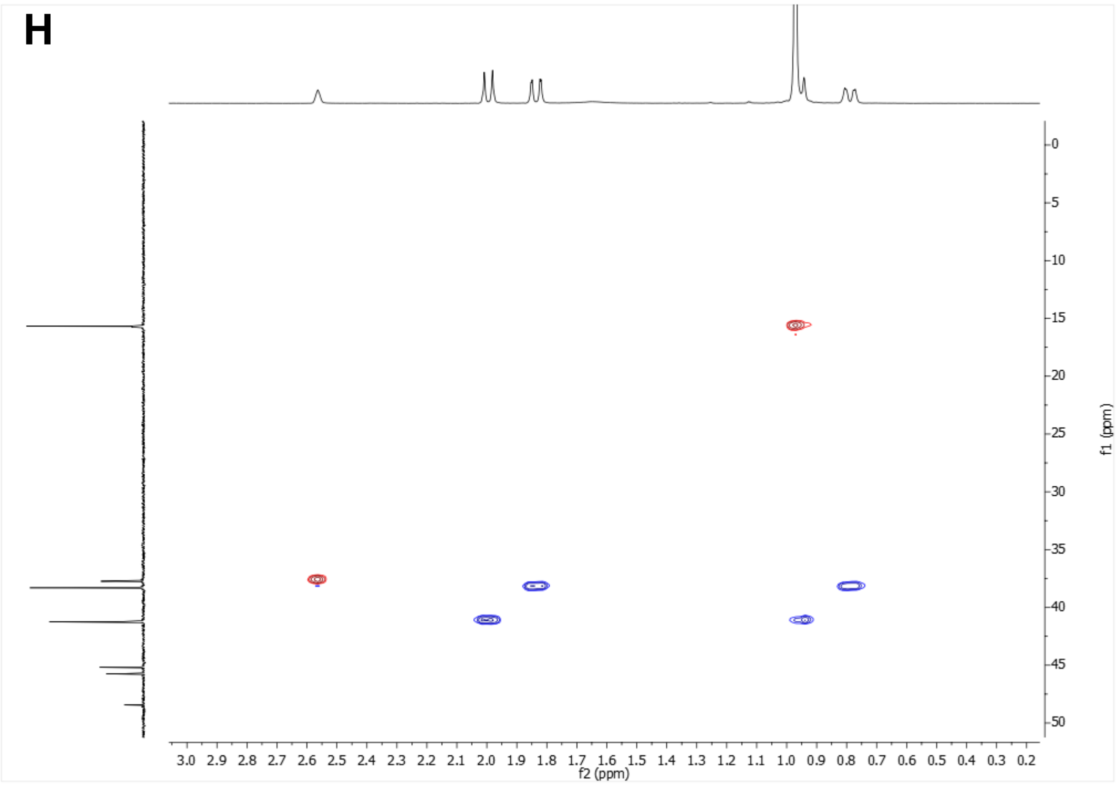

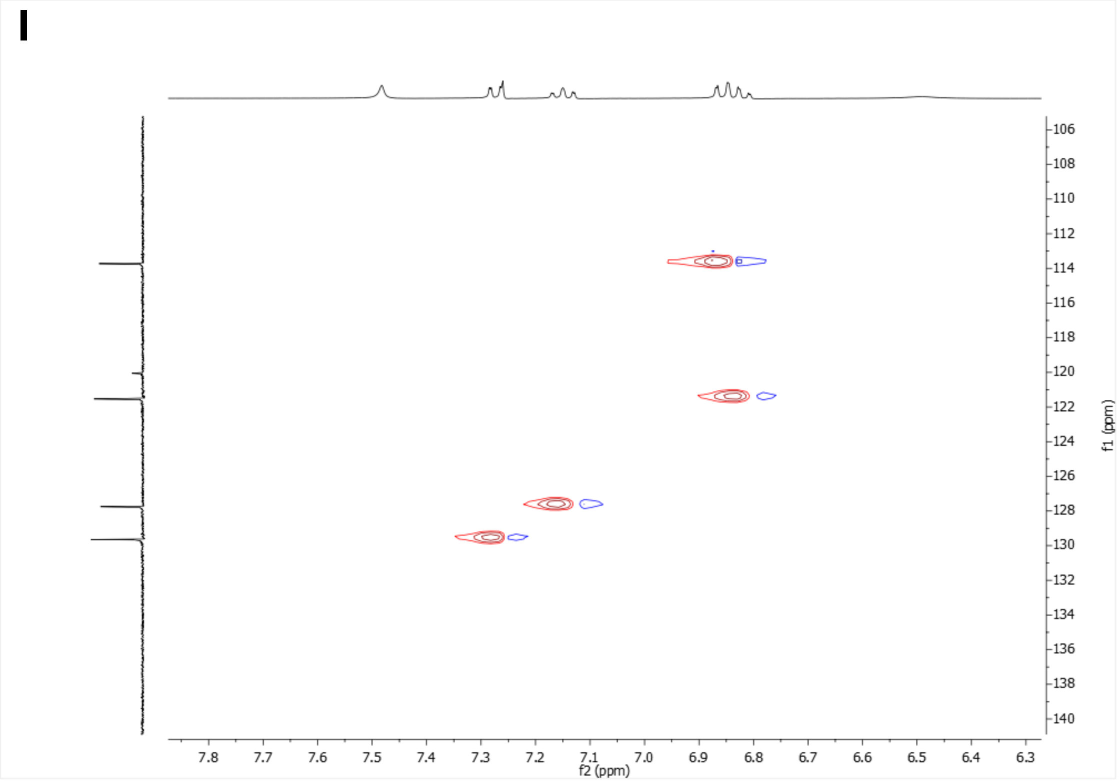

Supplementary Fig. 14: Characterization of *N'*-(2-chlorophenyl)-3,4,8,9-tetramethyltetracyclo[4.4.0.0^{3,9}.0^{4,8}]decane-1-carbohydrazide (UB-MBX-46). (A-C) ^{1}H NMR spectrum. (D-F) ^{13}C NMR spectrum. (G-I) HSQC spectrum.

^1H NMR (400 MHz, $CDCl_3$)

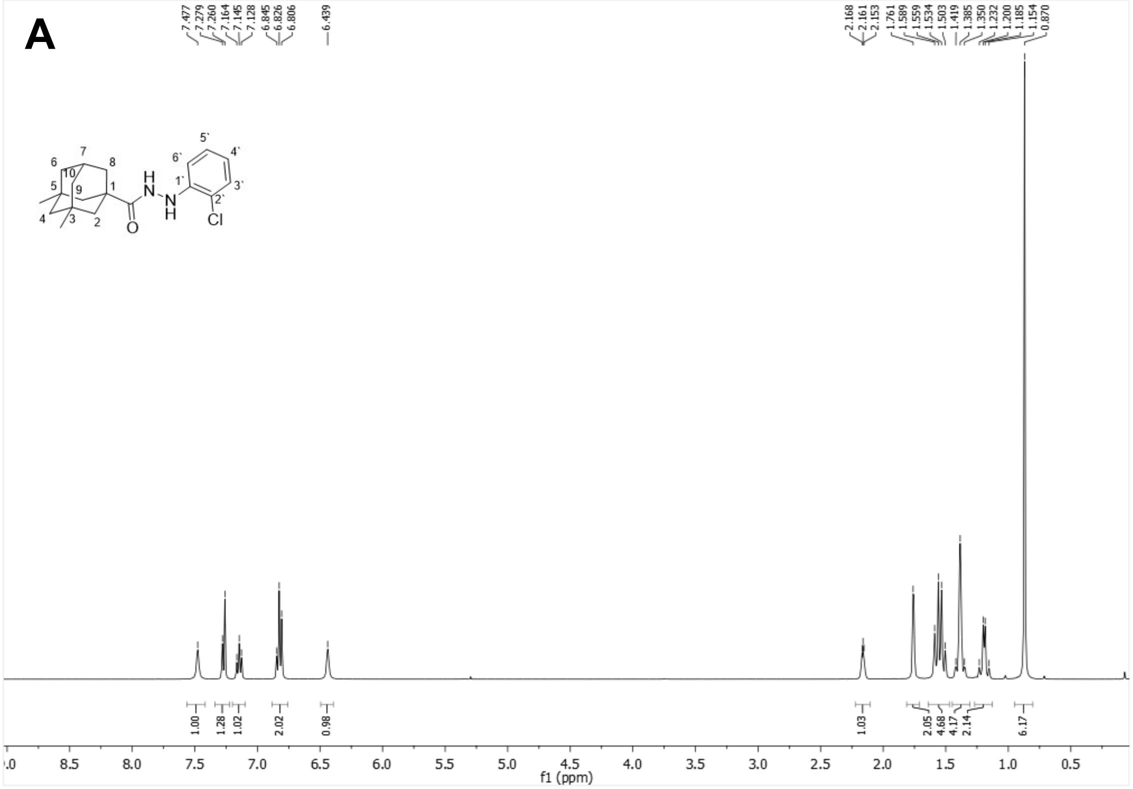

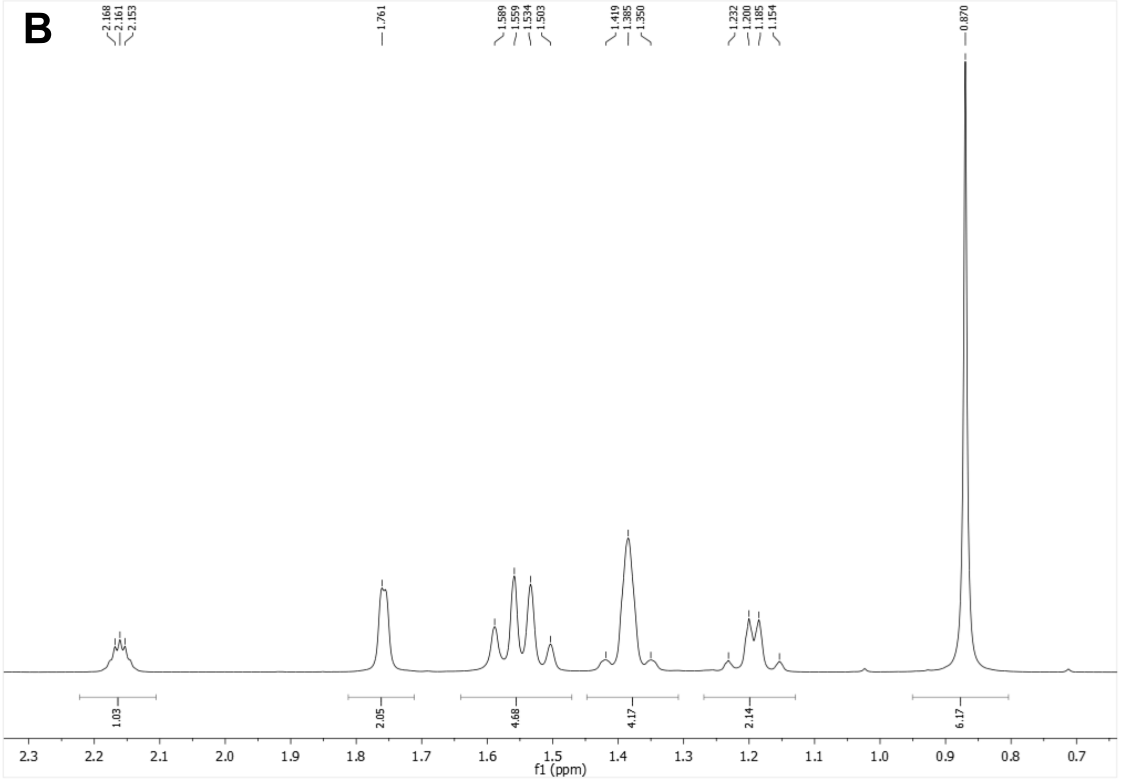

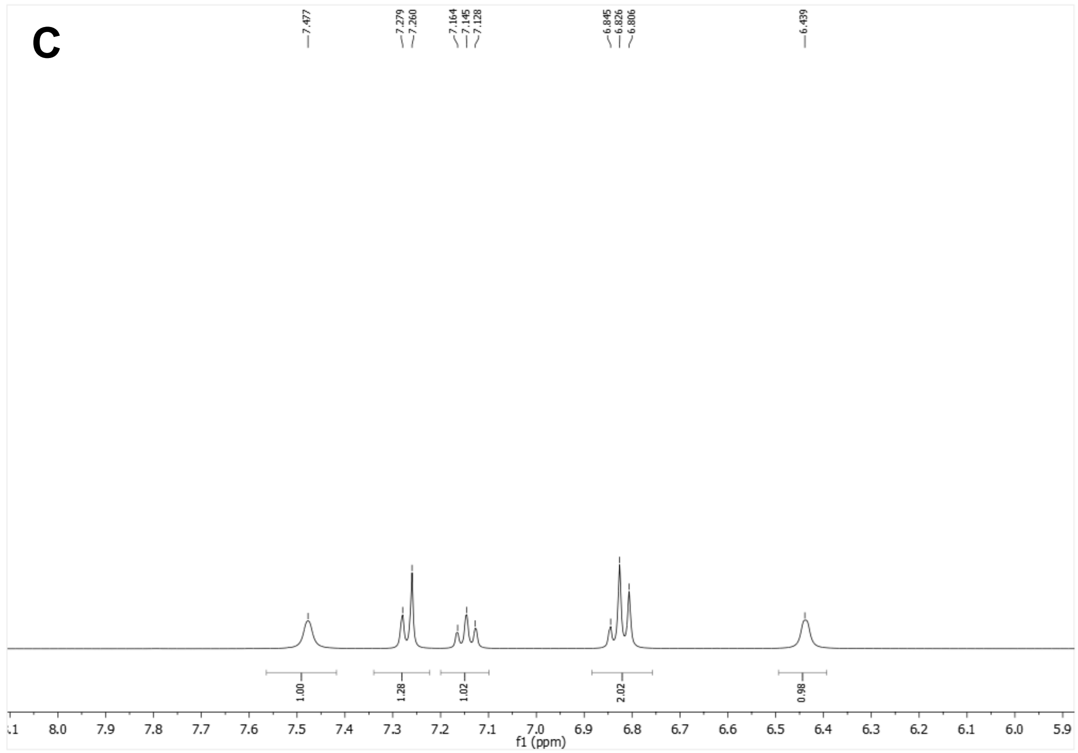

^{13}C-NMR (100.6 MHz, $CDCl_3$)

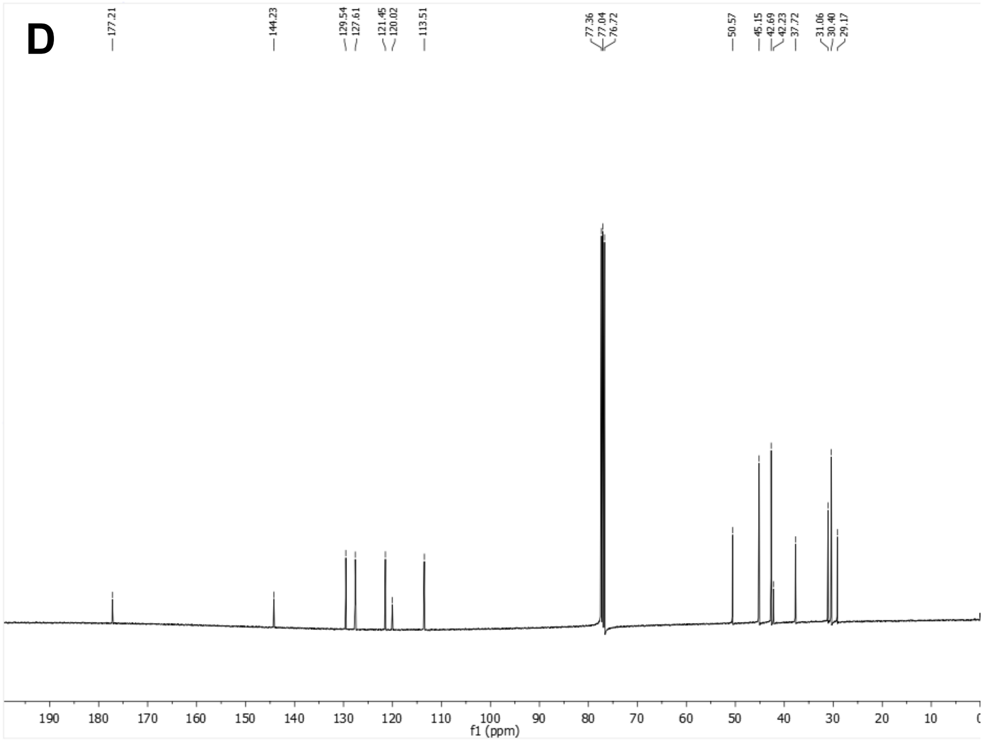

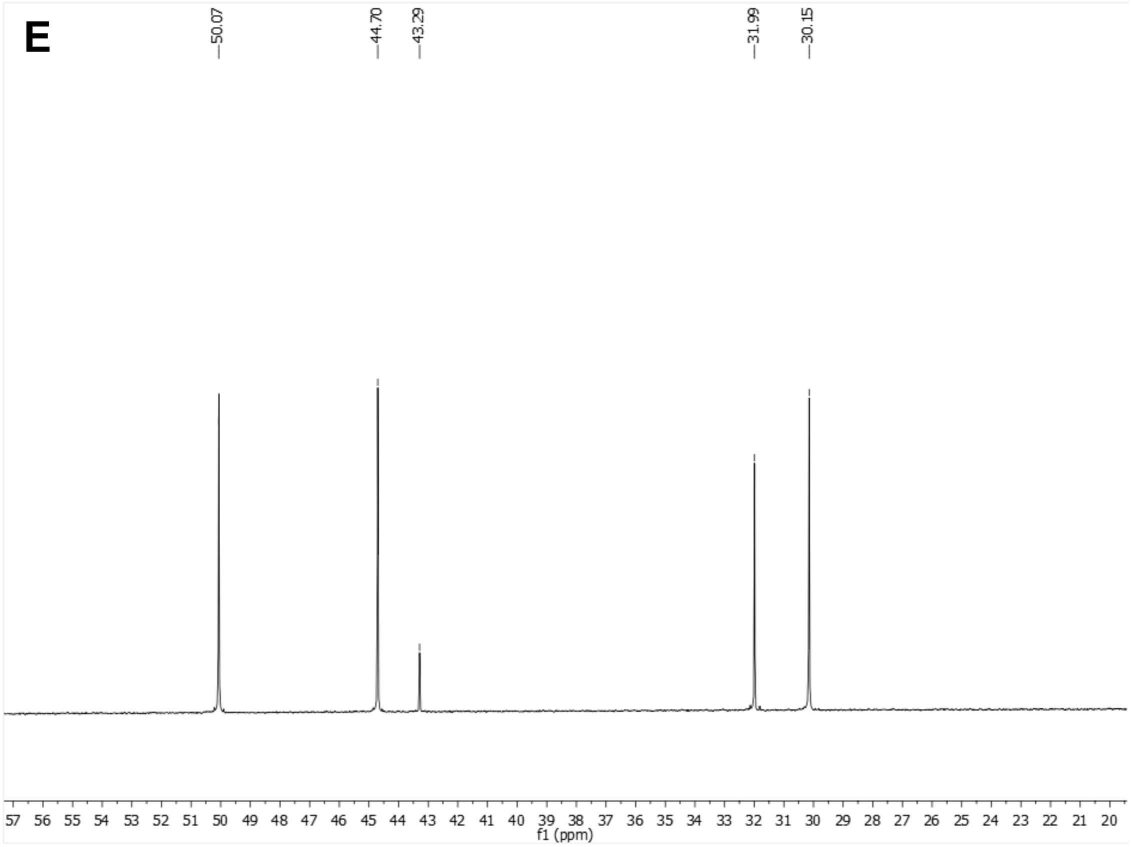
E
50.07
44.70
43.29
31.99
30.15
57 56 55 54 53 52 51 50 49 48 47 46 45 44 43 42 41 40 39 38 37 36 35 34 33 32 31 30 29 28 27 26 25 24 23 22 21 20
f1 (ppm)

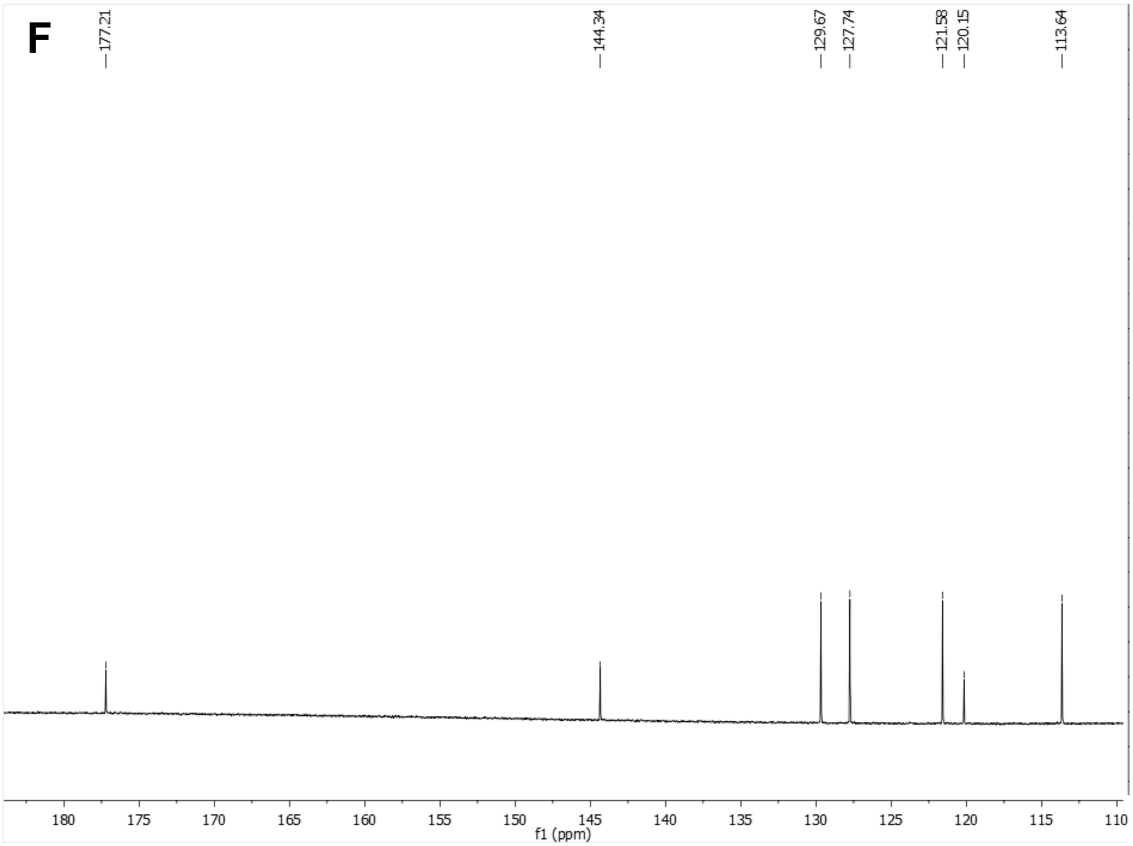
F
177.21
144.34
129.67
127.74
121.58
120.15
113.64
180 175 170 165 160 155 150 145 140 135 130 125 120 115 110
f1 (ppm)

HSQC ($CDCL_3$)

G

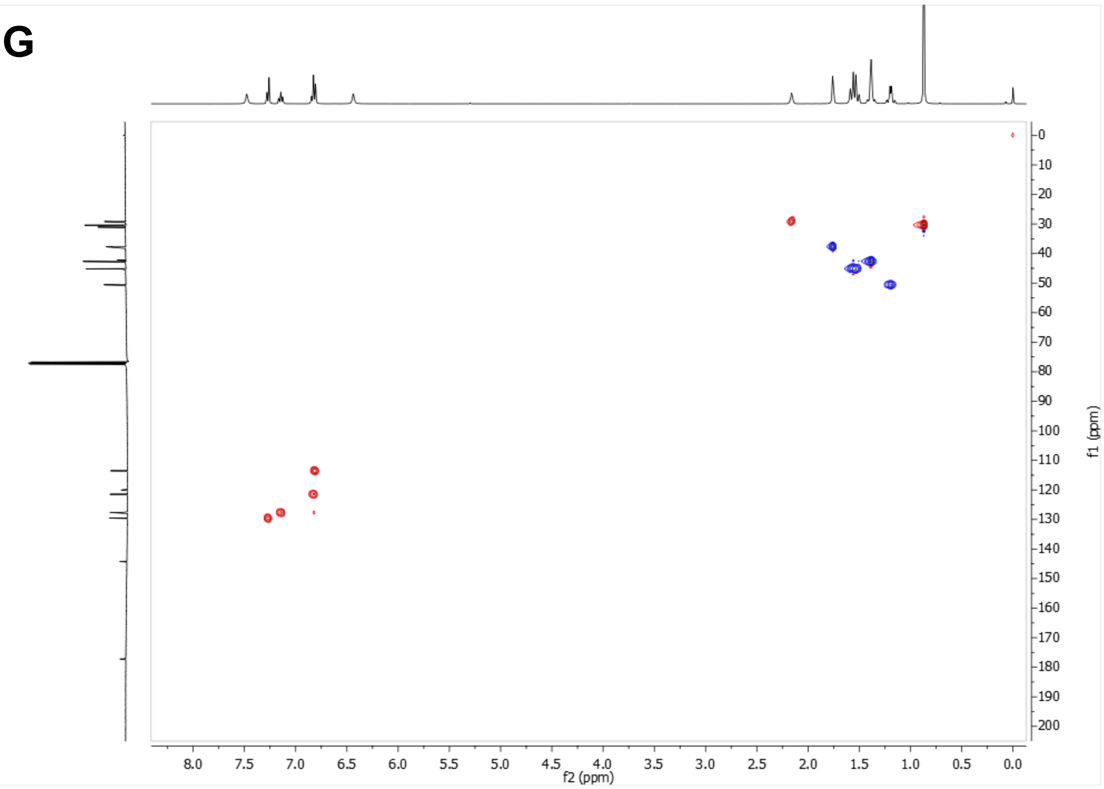

H

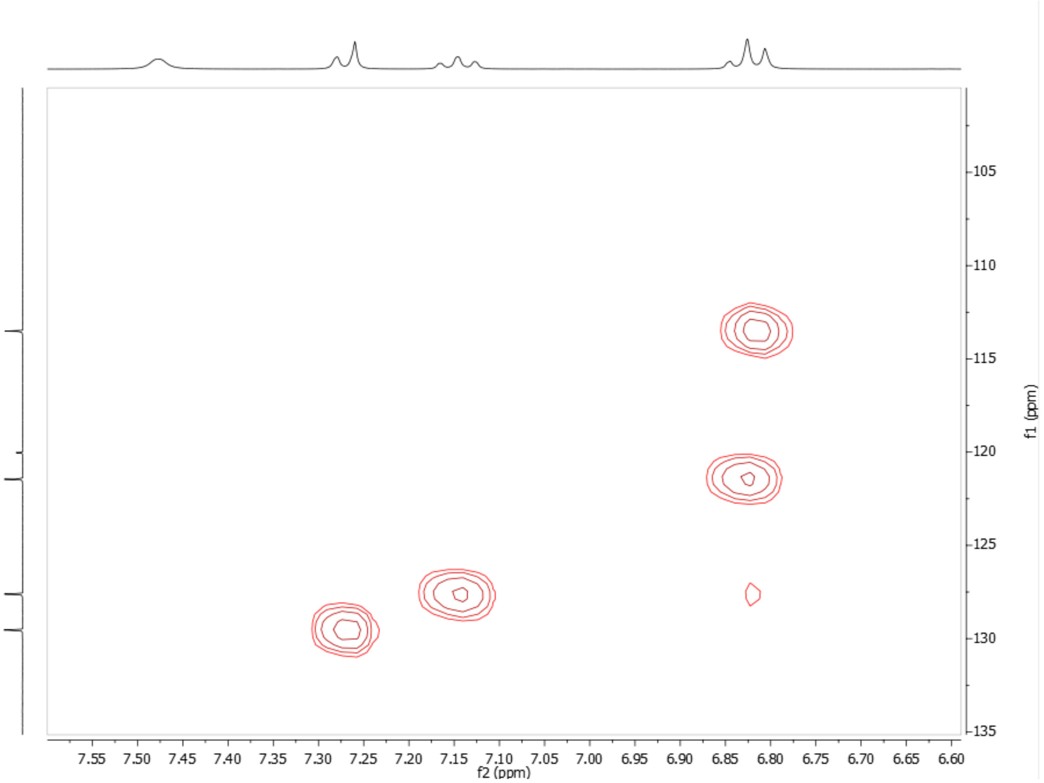

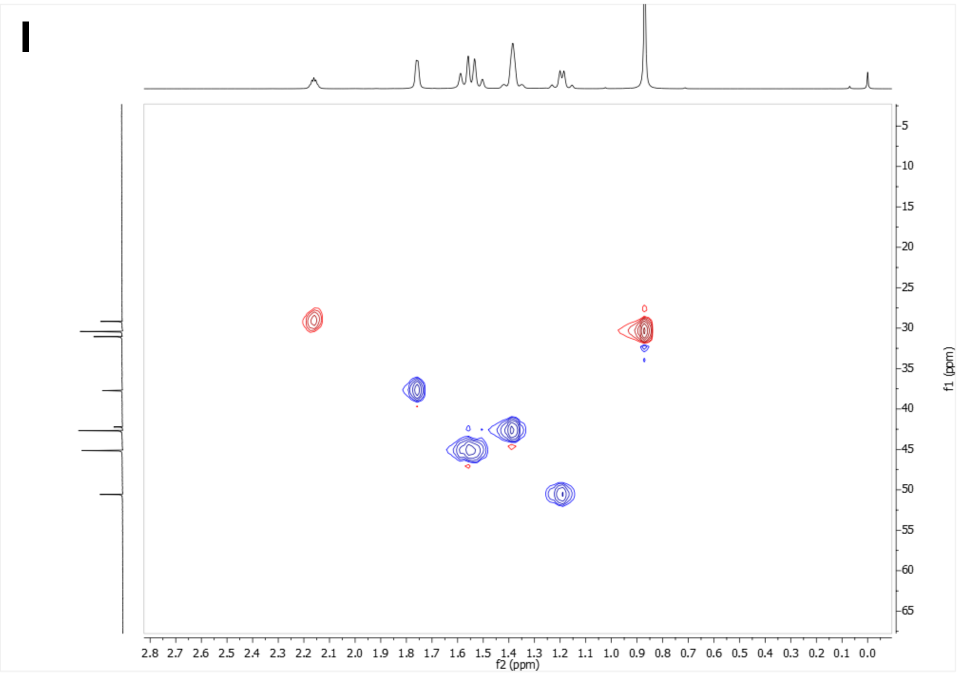

COSY ($CDCl_3$)

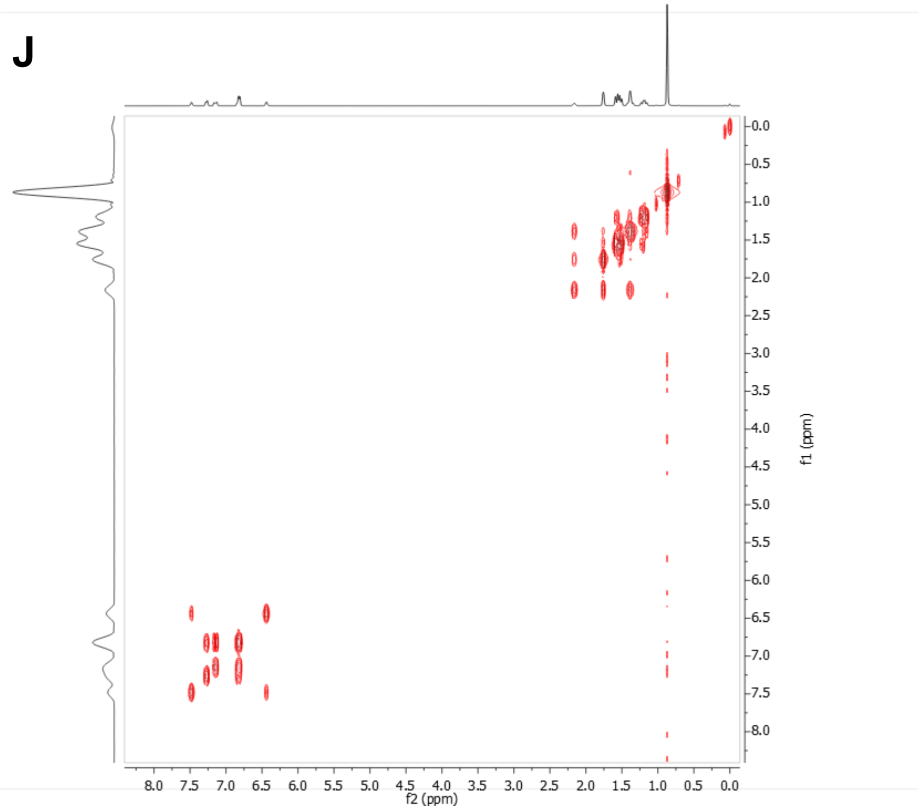

Supplementary Fig. 15: Characterization of *N'*-(2-chlorophenyl)-3,5-dimethyladamantane-1-carbohydrazide (UB-ALT-36). (A-C) ^{1}H NMR spectrum. (D-F) ^{13}C NMR spectrum. (G-I) HSQC spectrum. (J) COSY spectrum.

^1H NMR (400 MHz, $CDCl_3$)

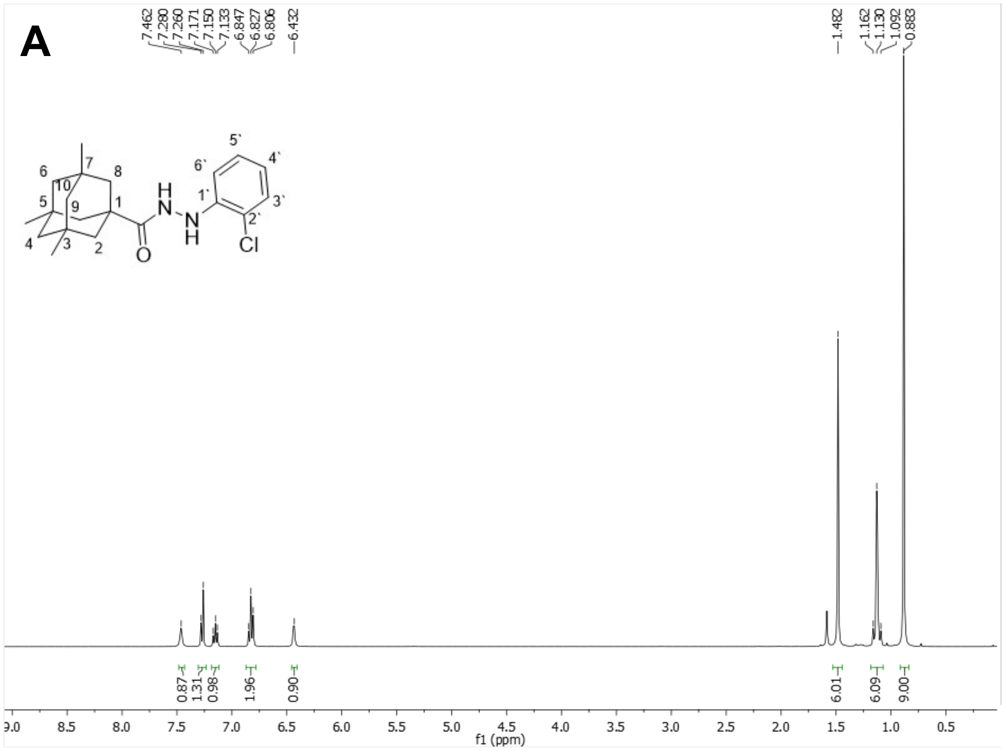

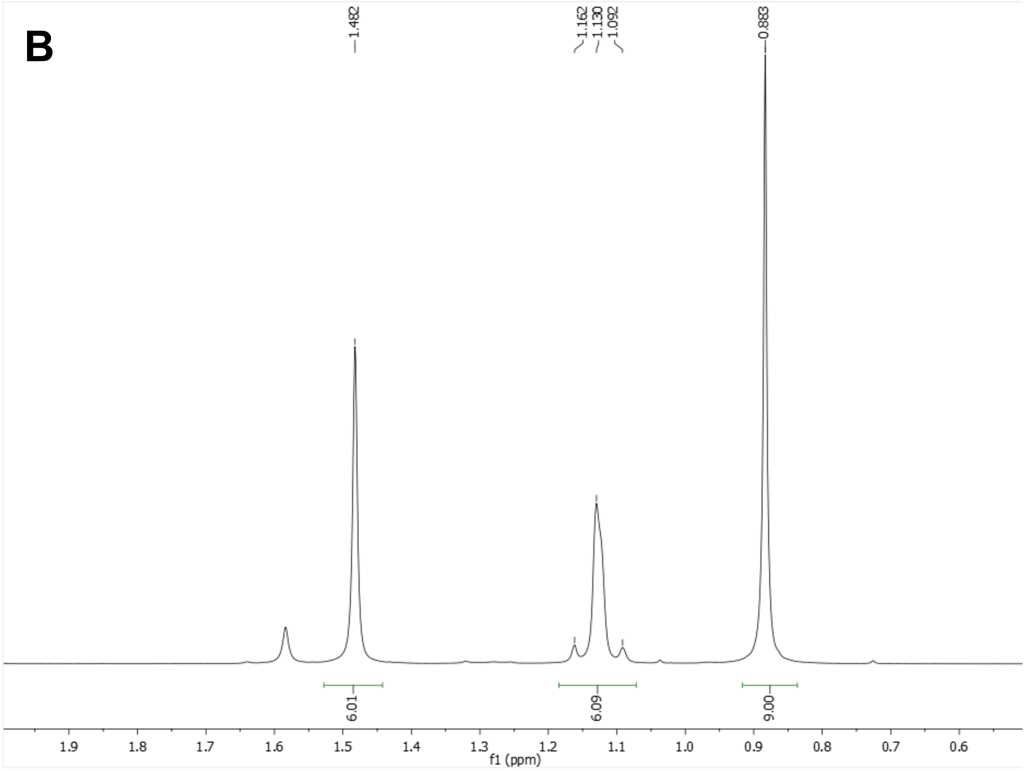

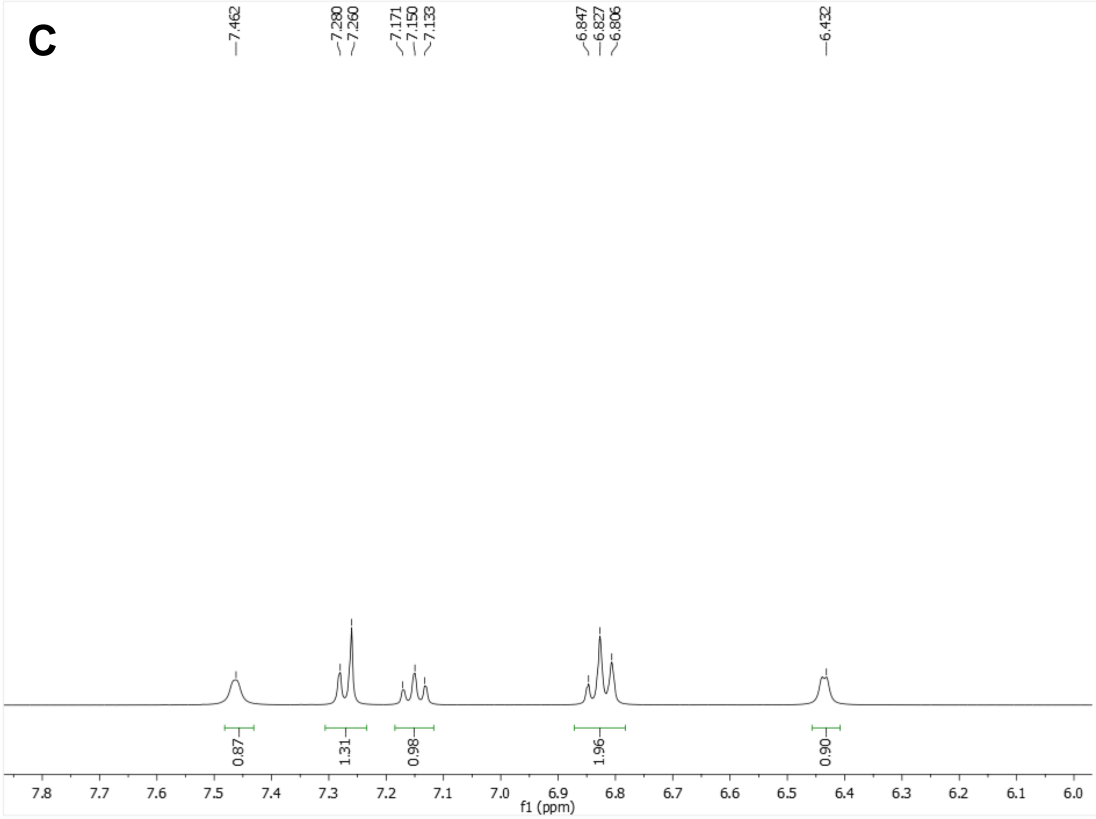

^{13}C-NMR (100.6 MHz, $CDCl_3$)

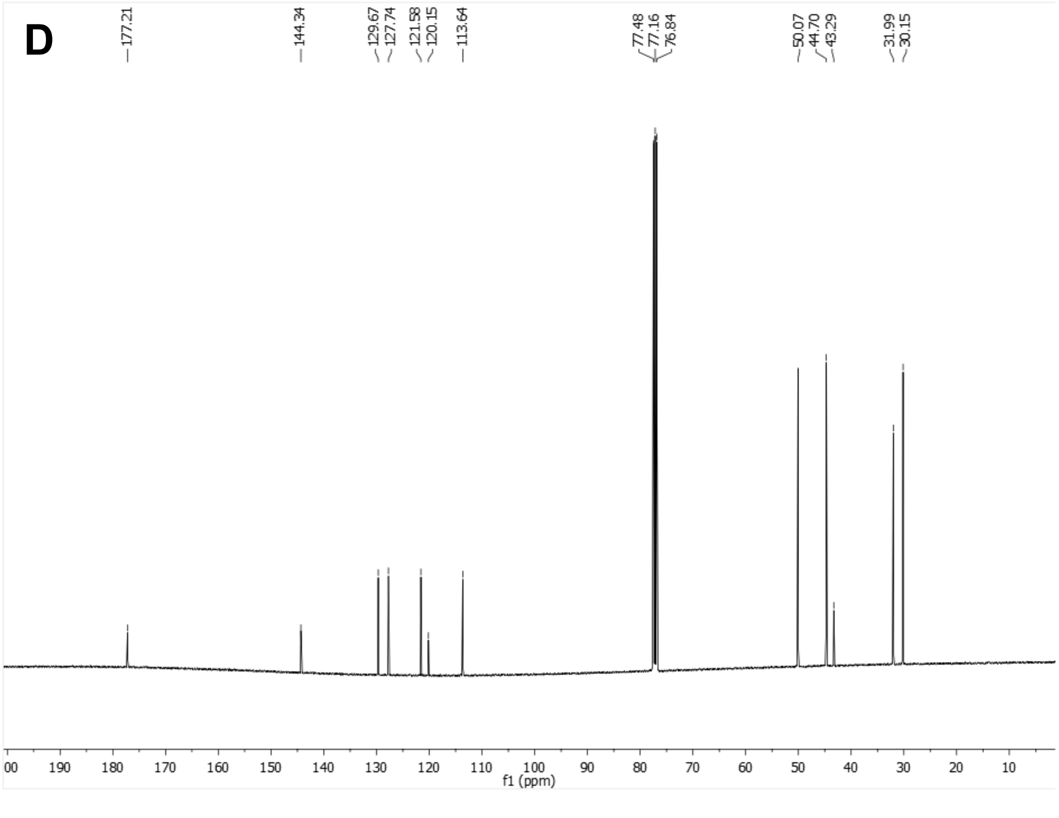

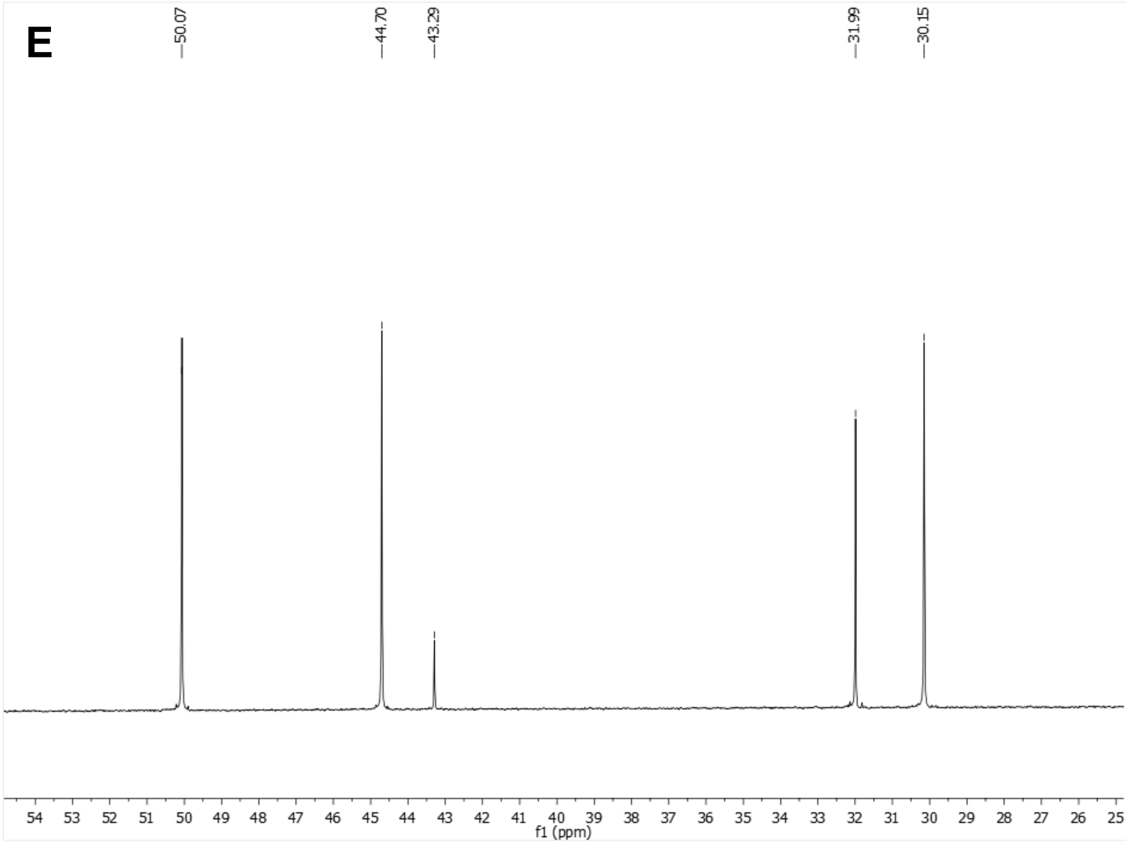
E
50.07
44.70
43.29
31.99
30.15
54 53 52 51 50 49 48 47 46 45 44 43 42 41 40 39 38 37 36 35 34 33 32 31 30 29 28 27 26 25
f1 (ppm)

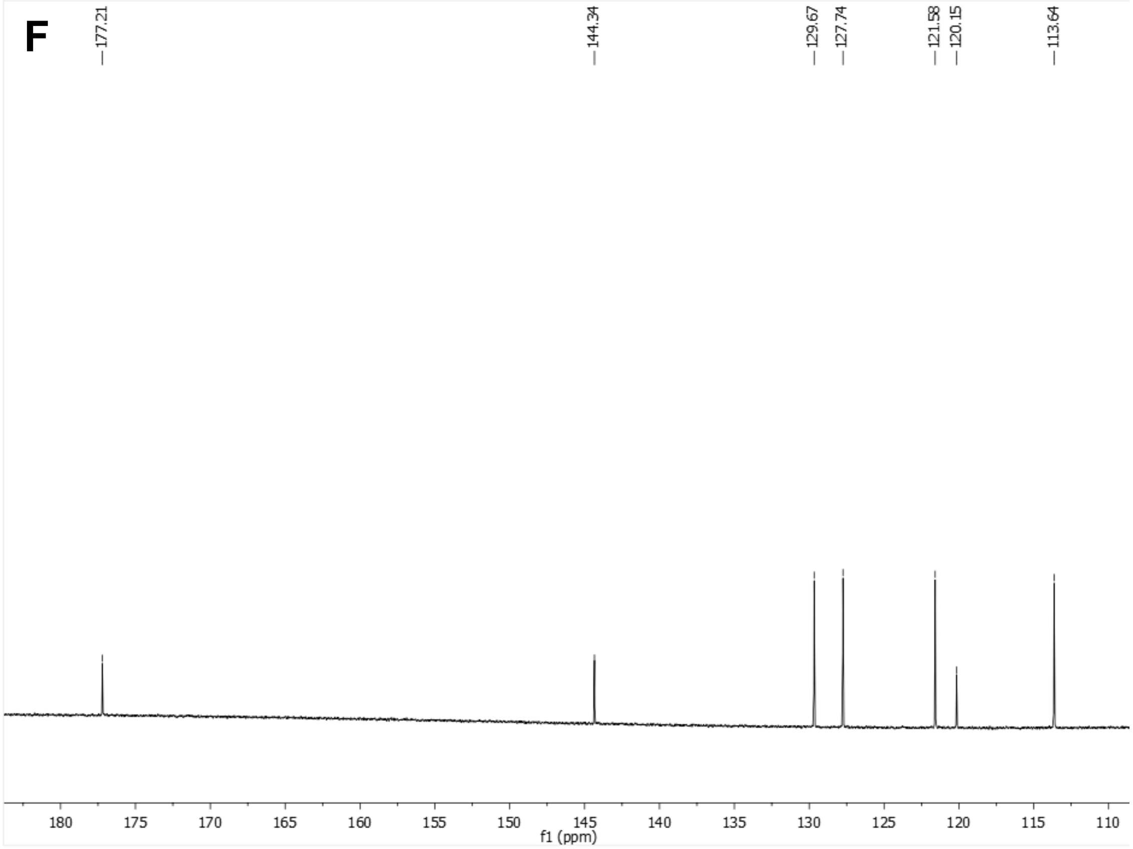
F
177.21
144.34
129.67
127.74
121.58
120.15
113.64
180 175 170 165 160 155 150 145 140 135 130 125 120 115 110
f1 (ppm)

HSQC ($CDCl_3$)

G

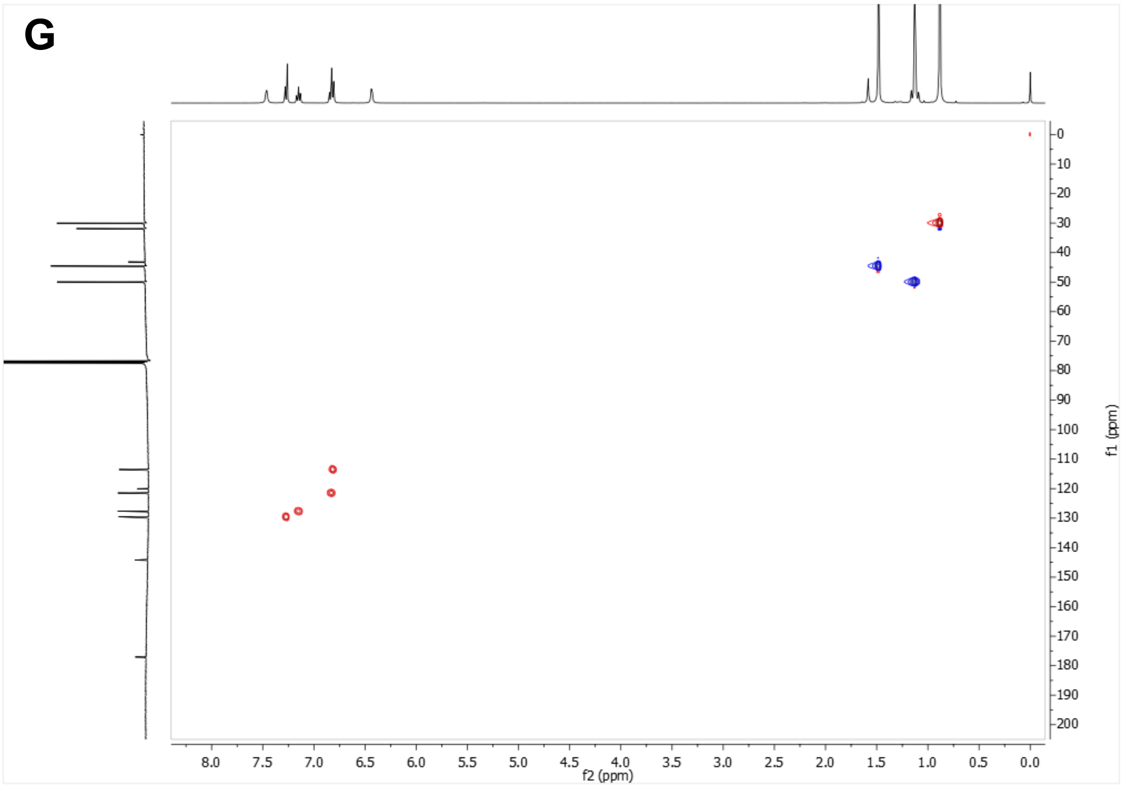

H

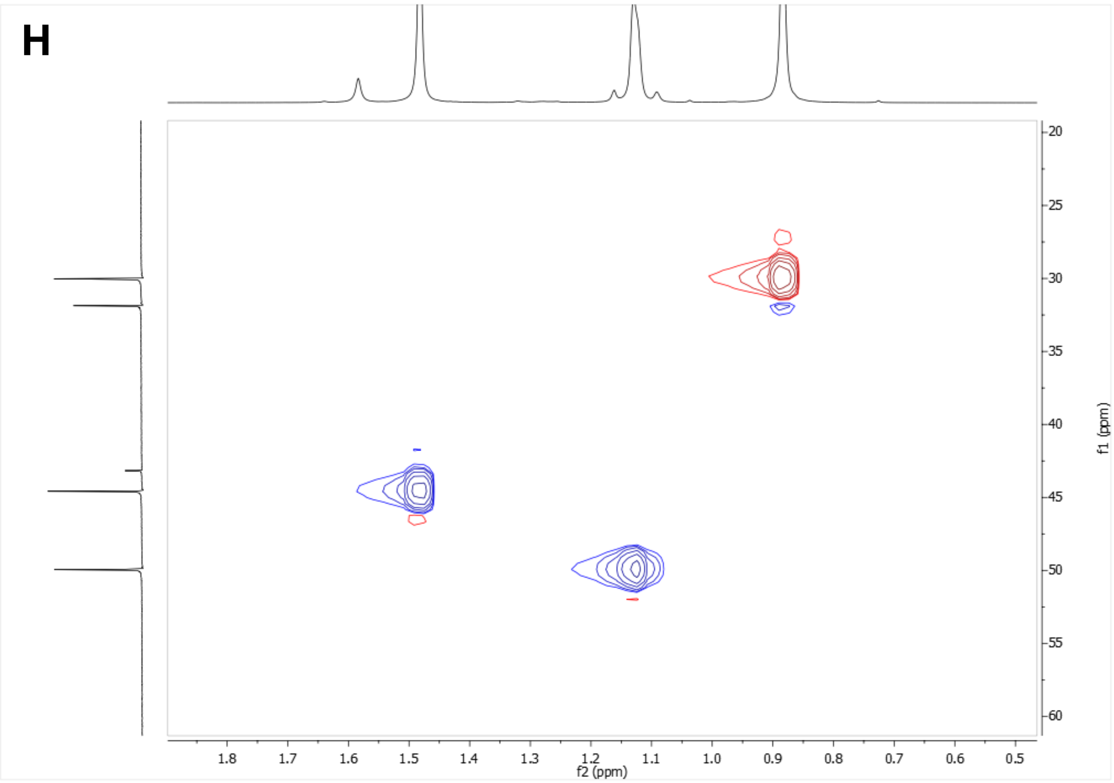

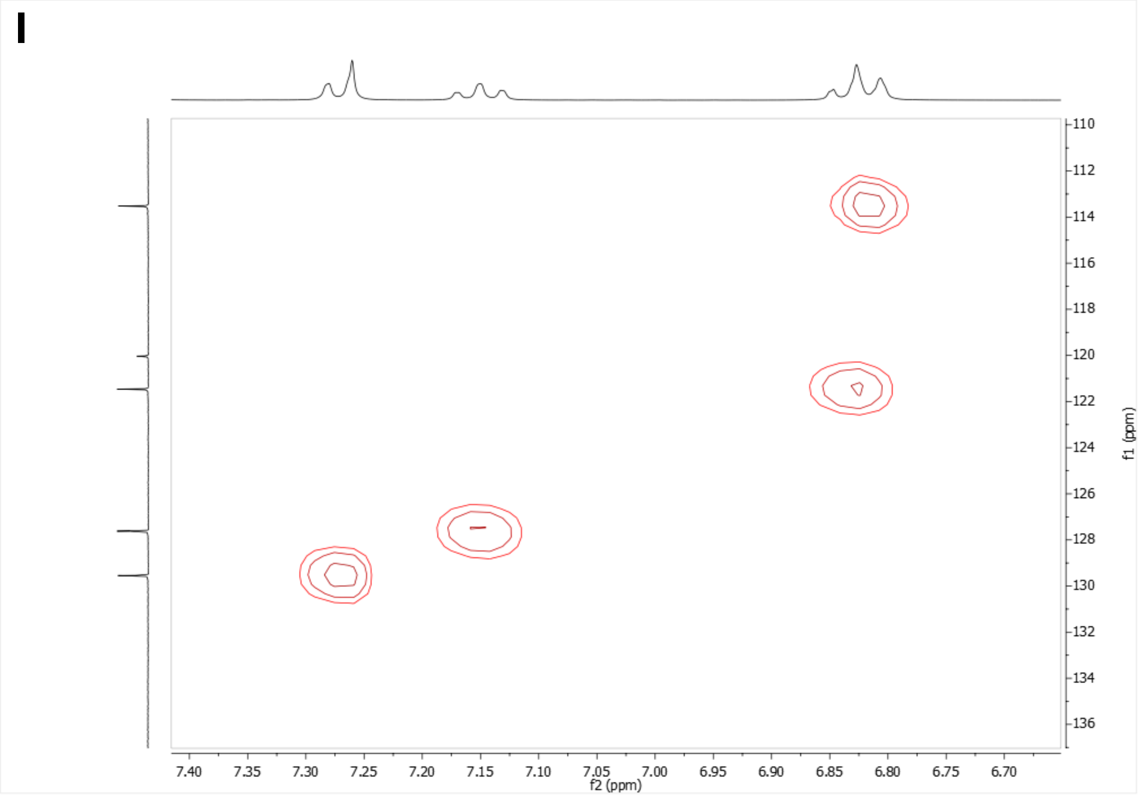
I
7.40
7.35
7.30
7.25
7.20
7.15
7.10
7.05
7.00
6.95
6.90
6.85
6.80
6.75
6.70
f2 (ppm)
110
112
114
116
118
120
122
124
126
128
130
132
134
136
f1 (ppm)

COSY ($CDCl_3$)

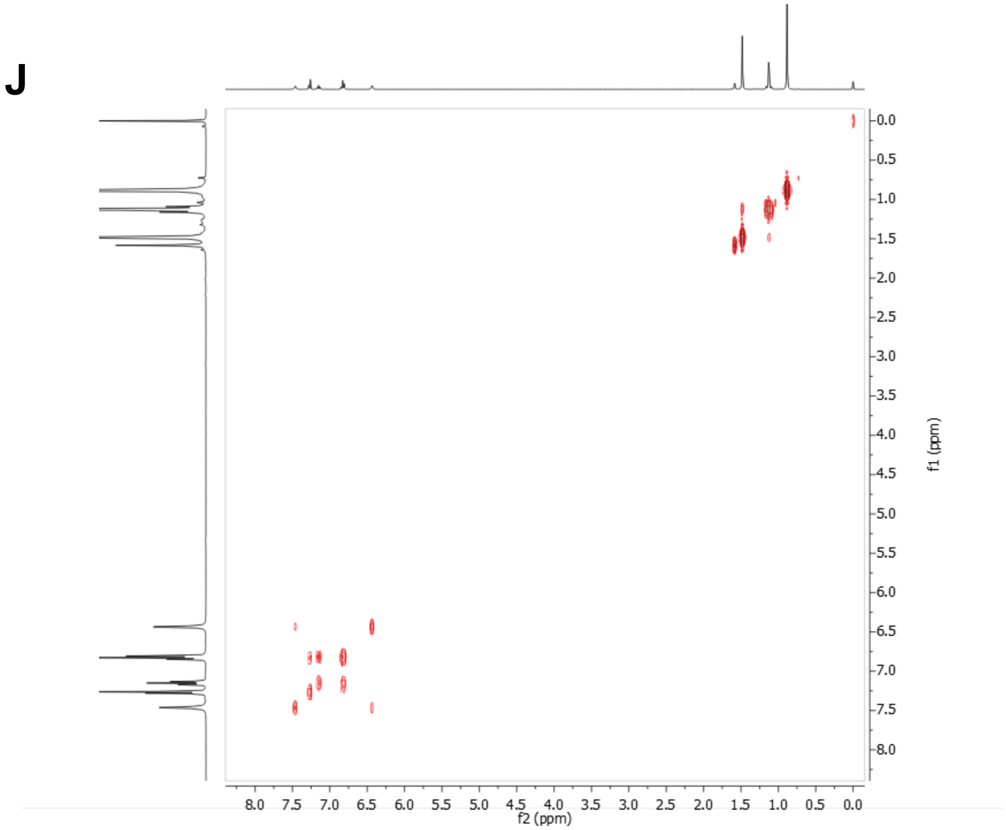
J
8.0
7.5
7.0
6.5
6.0
5.5
5.0
4.5
4.0
3.5
3.0
2.5
2.0
1.5
1.0
0.5
0.0
f2 (ppm)
f1 (ppm)

Supplementary Fig. 16: Characterization of *N'*-(2-chlorophenyl)-3,5,7-trimethyladamantane-1-carbohydrazide (UB-ALT-P37). (A-C) ^1H NMR spectrum. (D-F) ^{13}C NMR spectrum. (G-I) HSQC spectrum. (J) COSY spectrum.

^{1}H NMR (400 MHz, $CDCl_3$

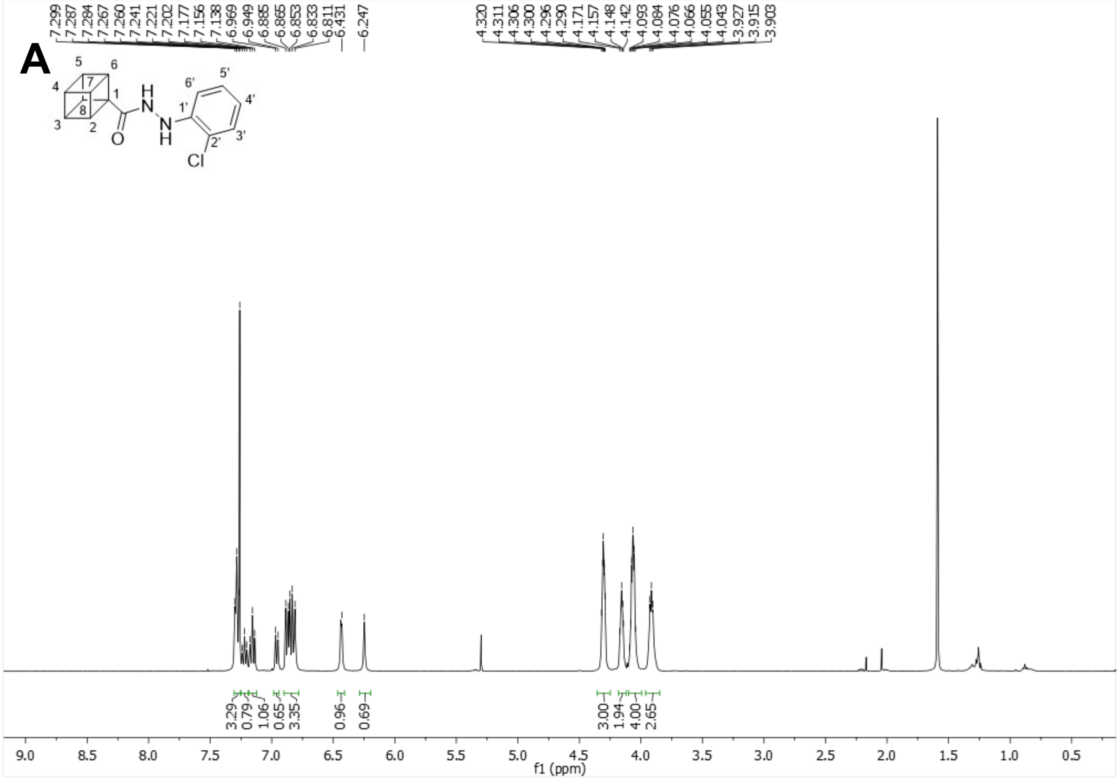

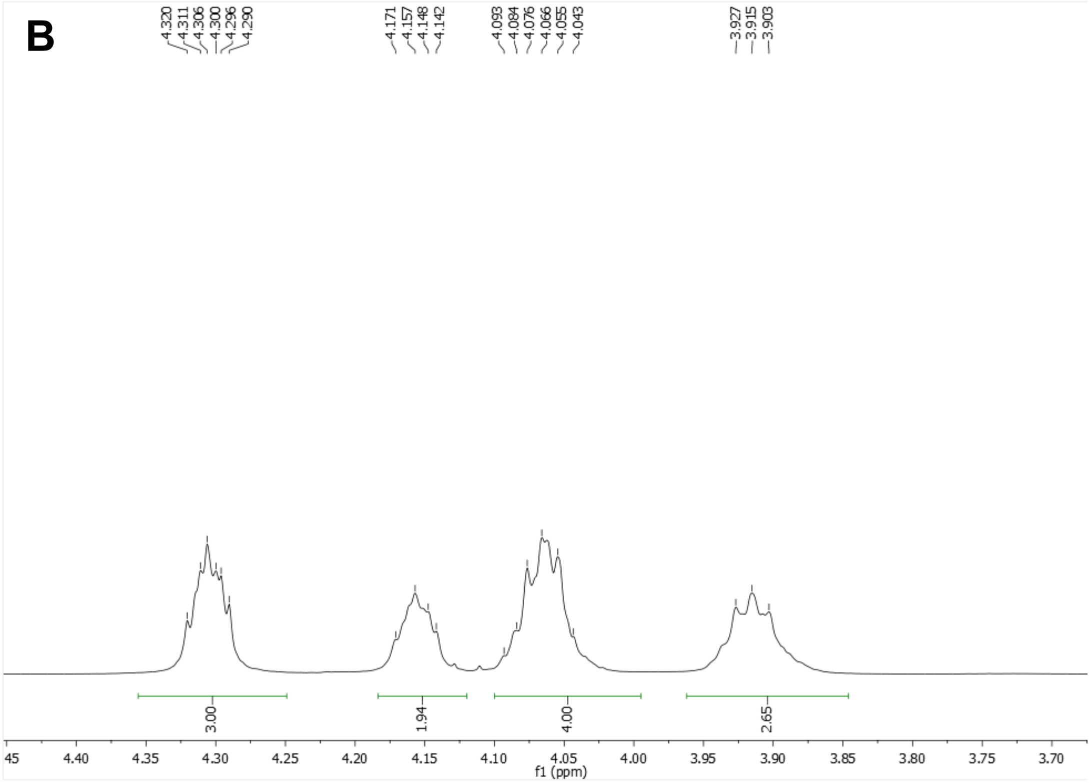

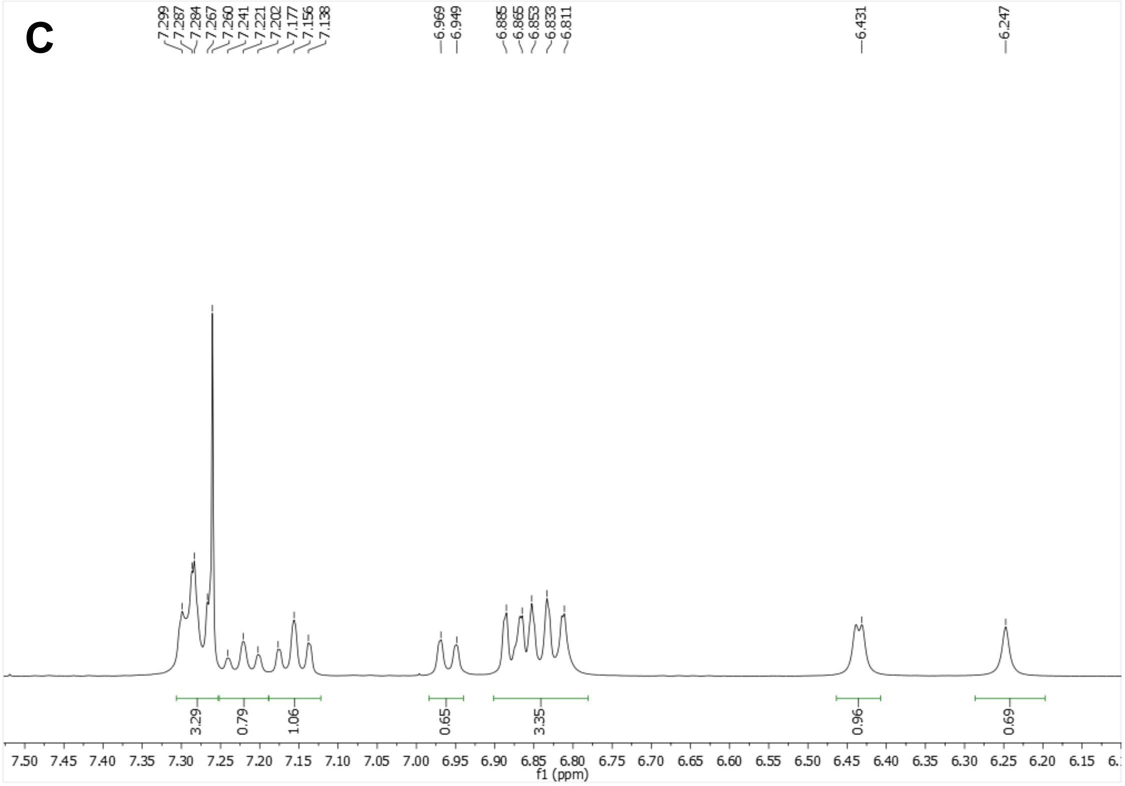

^{13}C-NMR (100.6 MHz, $CDCl_3$)

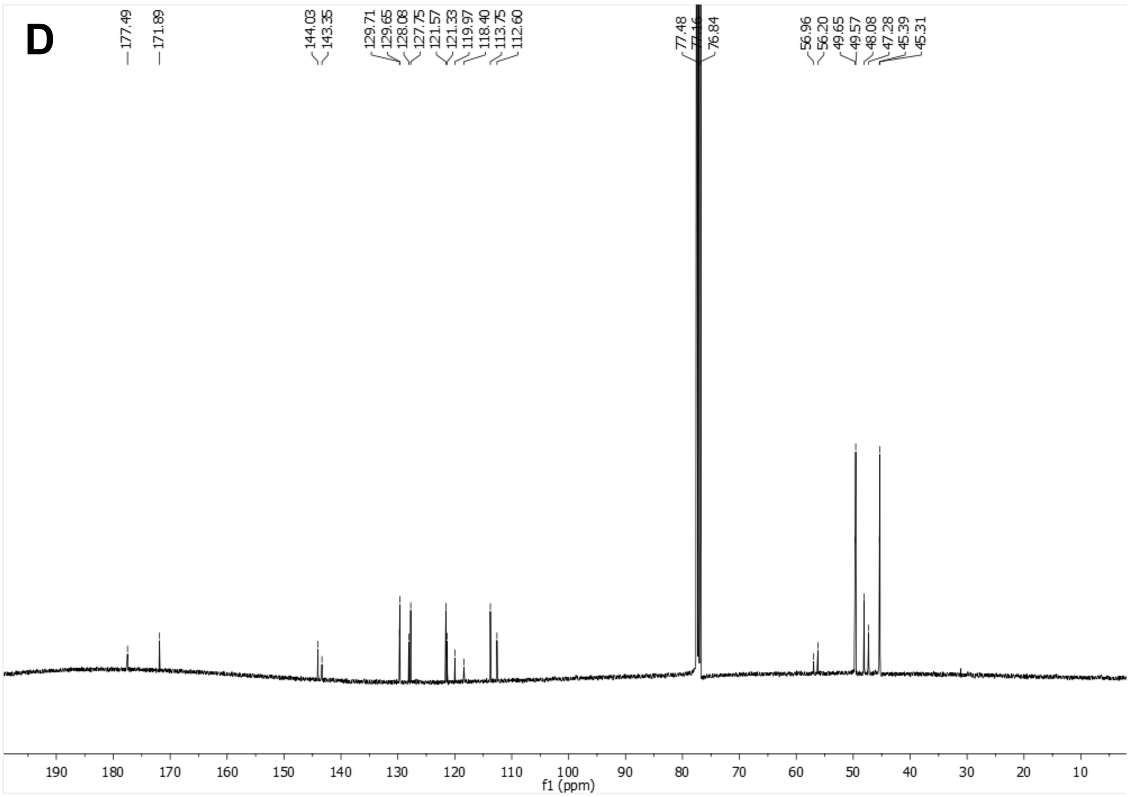

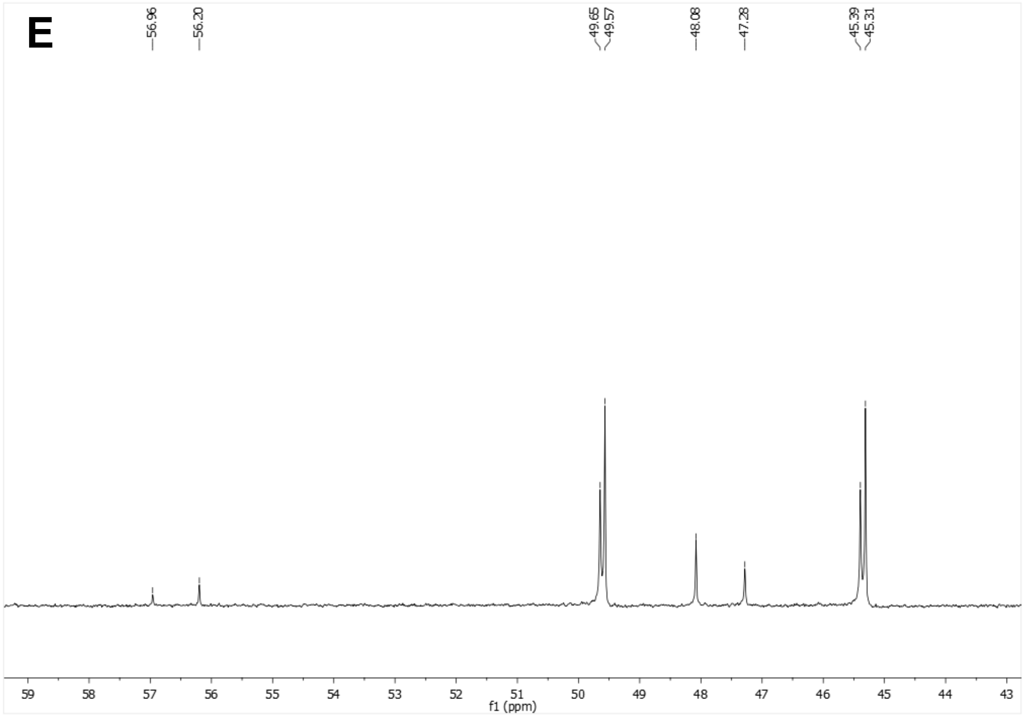
E
56.96
56.20
49.65
49.57
48.08
47.28
45.39
45.31
59 58 57 56 55 54 53 52 51 50 49 48 47 46 45 44 43
f1 (ppm)

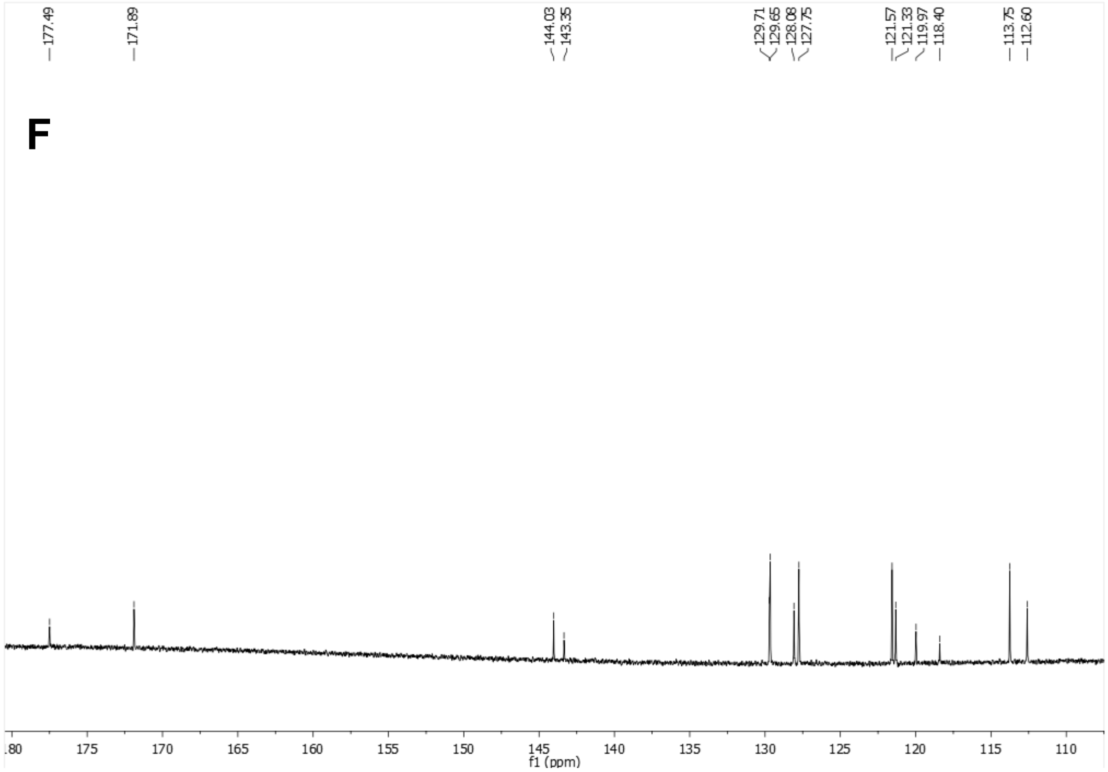
F
177.49
171.89
144.03
143.35
129.71
129.65
128.08
127.75
121.57
121.33
119.97
118.40
113.75
112.60
180 175 170 165 160 155 150 145 140 135 130 125 120 115 110
f1 (ppm)

HSQC ($CDCl_3$)

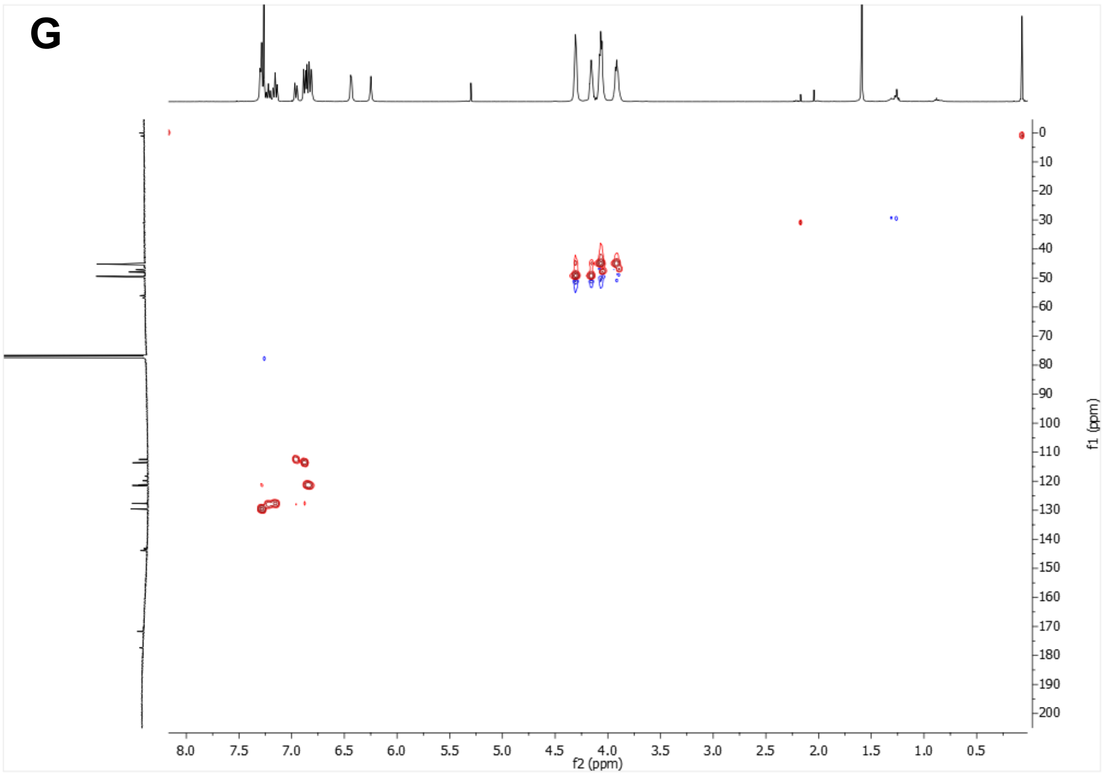
G
f2 (ppm)
f1 (ppm)
8.0
7.5
7.0
6.5
6.0
5.5
5.0
4.5
4.0
3.5
3.0
2.5
2.0
1.5
1.0
0.5
0
10
20
30
40
50
60
70
80
90
100
110
120
130
140
150
160
170
180
190
200

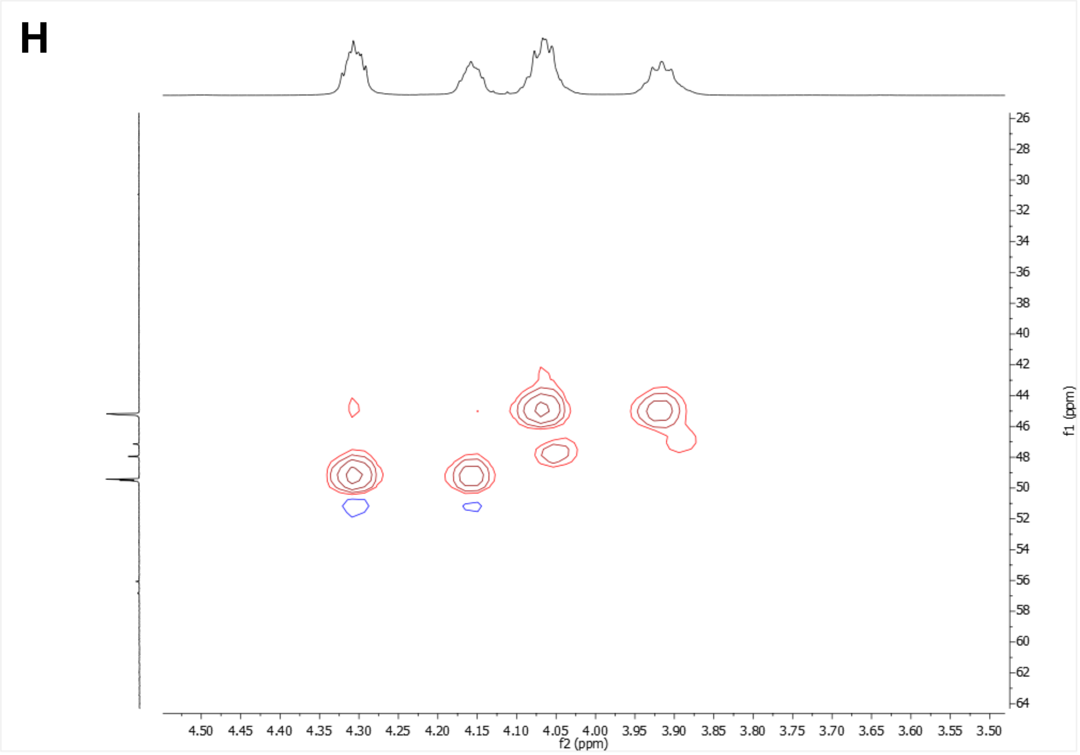
H
f2 (ppm)
f1 (ppm)
4.50
4.45
4.40
4.35
4.30
4.25
4.20
4.15
4.10
4.05
4.00
3.95
3.90
3.85
3.80
3.75
3.70
3.65
3.60
3.55
3.50
26
28
30
32
34
36
38
40
42
44
46
48
50
52
54
56
58
60
62
64

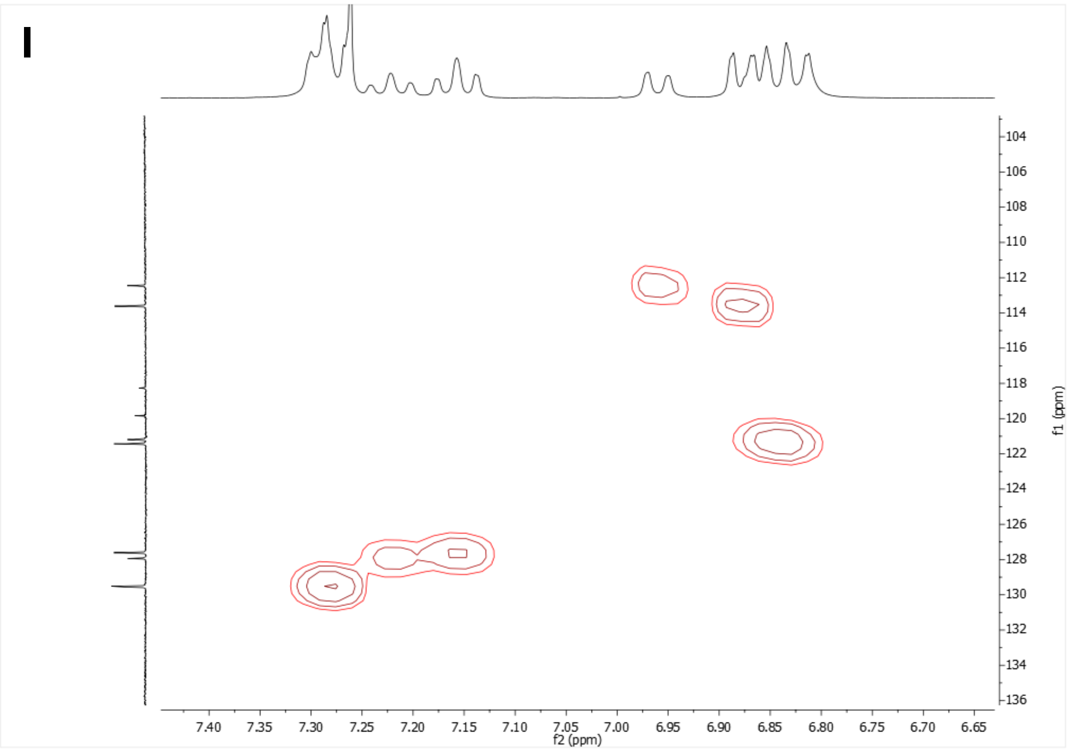
I
7.40
7.35
7.30
7.25
7.20
7.15
7.10
7.05
7.00
6.95
6.90
6.85
6.80
6.75
6.70
6.65
f2 (ppm)
104
106
108
110
112
114
116
118
120
122
124
126
128
130
132
134
136
f1 (ppm)

COSY ($CDCl_3$)

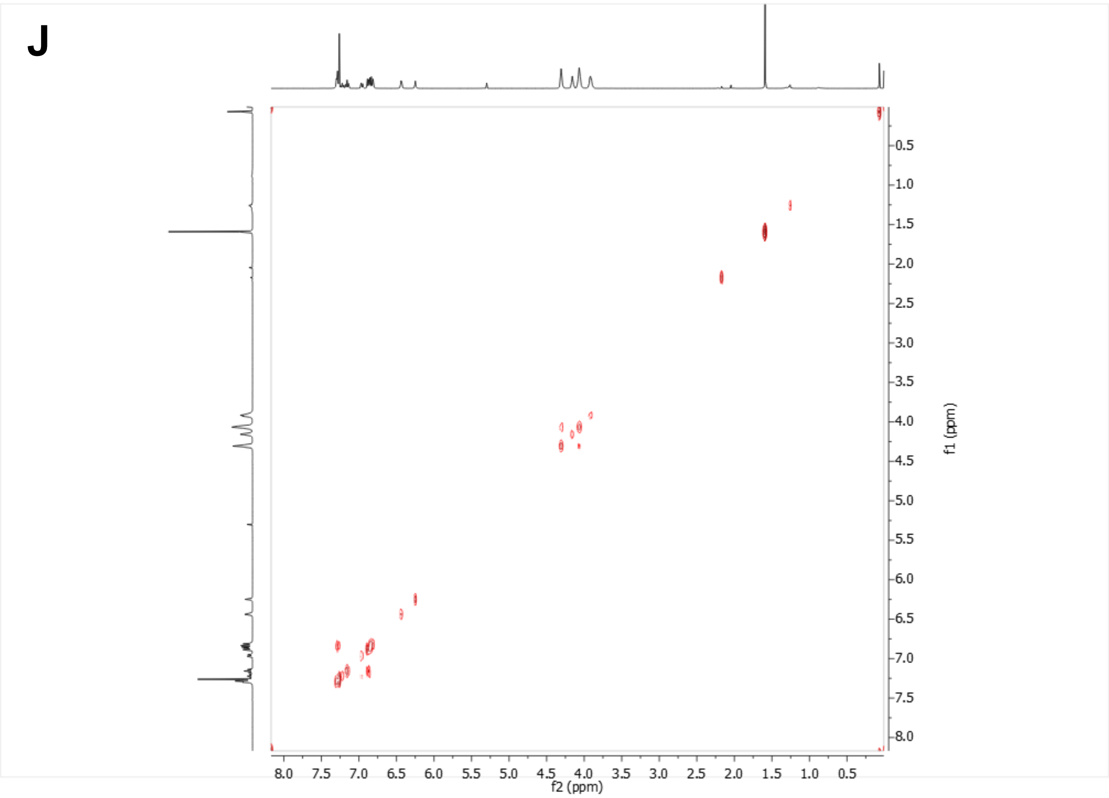
J
8.0
7.5
7.0
6.5
6.0
5.5
5.0
4.5
4.0
3.5
3.0
2.5
2.0
1.5
1.0
0.5
f2 (ppm)
f1 (ppm)

K

HPLC-UV at 254 nm

Blank

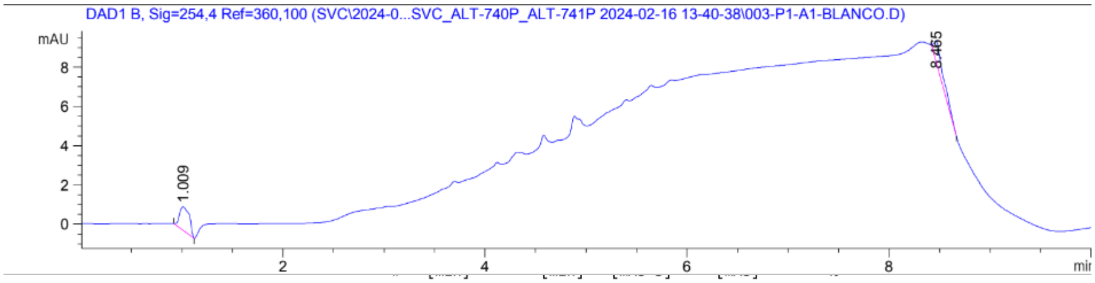

1	1.009	BB	0.1022	8.31069	1.17909	53.3178
2	8.465	BB	0.0943	7.27639	1.05744	46.6822
Totals :				15.58708	2.23653	

UB-ALT-P38

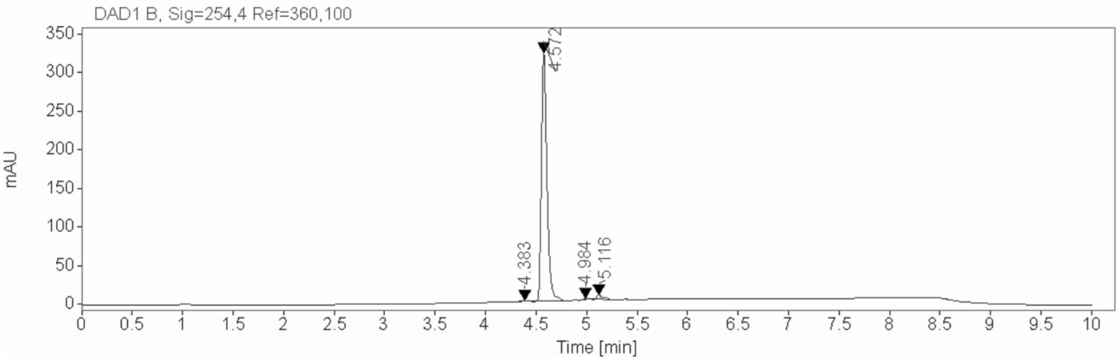

Signal: DAD1 B, Sig=254,4 Ref=360,100

RT [min]	Type	Width [min]	Area	Height	Area%	Name
4.383	BB	0.0579	7.7845	1.9803	0.6080	
4.572	BB	0.0584	1236.1952	324.8564	96.5513	
4.984	BB	0.0585	8.4846	2.2238	0.6627	
5.116	BB	0.0657	27.8861	6.0589	2.1780	
		Sum	1280.3504			

Supplementary Fig. 17: Characterization of *N'*-(2-chlorophenyl)cubane-1-carbohydrazide (UB-ALT-P38). (A-C) ^{1}H NMR spectrum. (D-F) ^{13}C NMR spectrum. (G-I) HSQC spectrum. (J) COSY spectrum. (K) HPLC-UV at 254 nm.

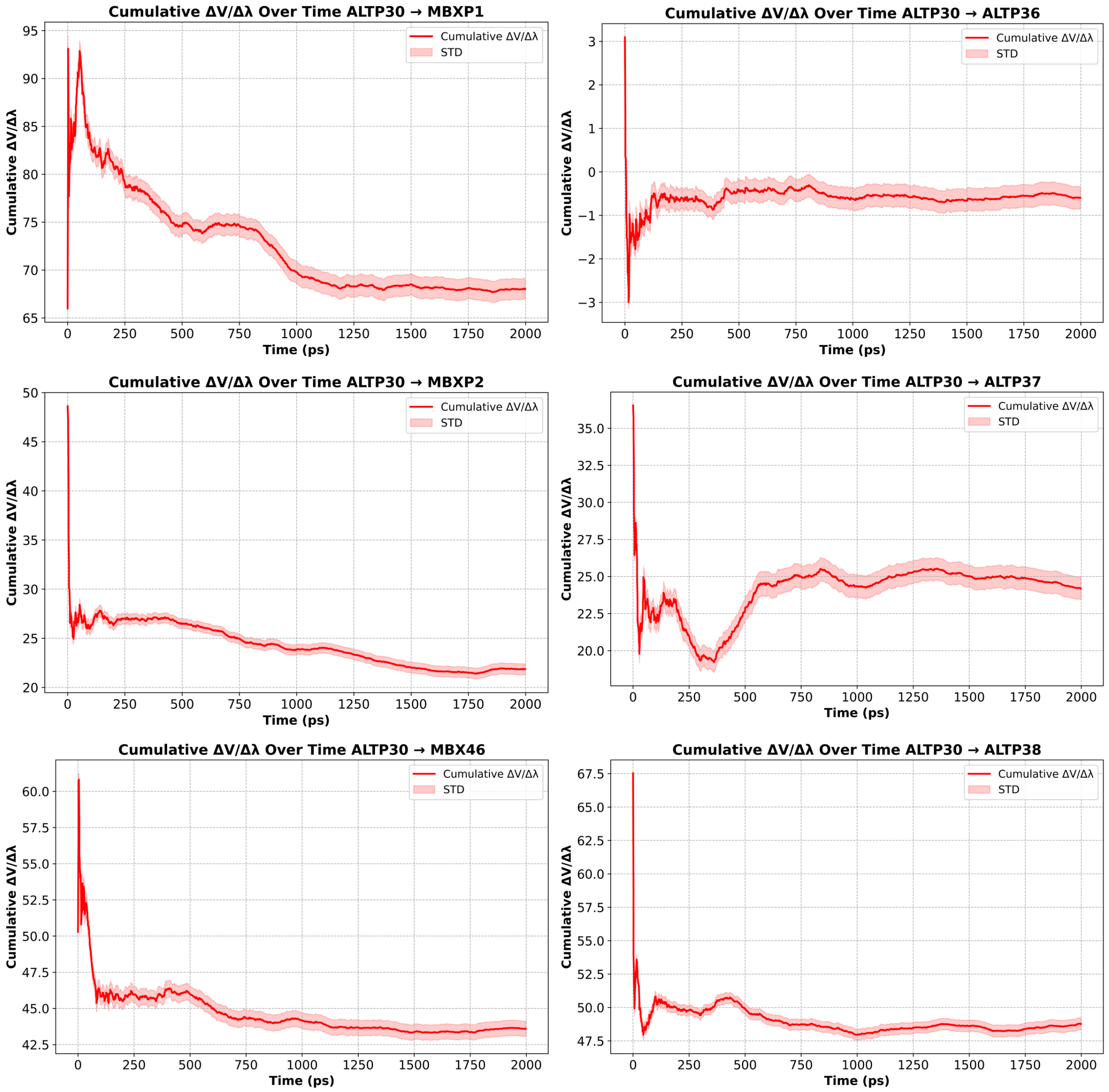

Supplementary Fig. 18: Convergence plots for the perturbative calculations of Table 1. Convergence plot showing the change of $\Delta V/\Delta \lambda$ during the 2-ns simulations for λ=11 at each perturbation transformation indicated in the respective panels. The perturbation transformation was performed on each of the three symmetry-related ligands bound to the three symmetry-related classical allosteric pockets. The plots indicate the simulations for each perturbation transformation reach equilibrium by 2 nanoseconds.

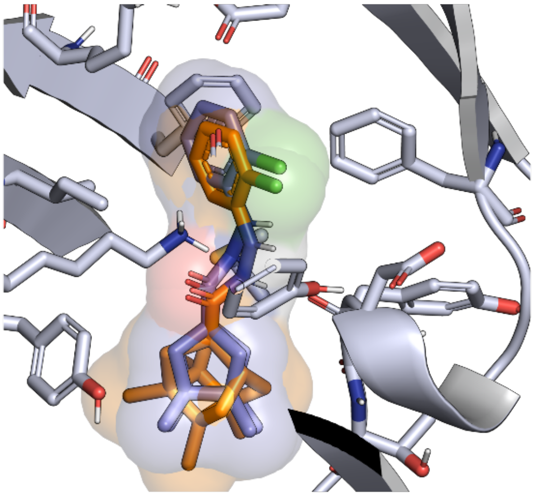

	UB-ALT-P36	UB-ALT-P37	UB-ALT-P38	UB-MBX-46	UB-MBX-P2
RMSD Ligand(Å)	1.15	1.48	0.90	0.98	0.74

Supplementary Fig. 19: (*Top*) The last snapshot of UB-MBX-46 bound to hP2X7R after a 50-ns MD simulation (purple sticks) superimposed on the final state of the 2-ns TI/MD simulation for the perturbative transformation of UB-ALT-P30-hP2X7R → UB-MBX-46-hP2X7R (UB-MBX-46 in orange sticks). The 2-ns MD simulation for each λ-state was enough for convergence (Supplementary Fig. 18). (*Bottom*) RMSD of each ligand in Table 1 after superposition of the corresponding last snaphsot after 50-ns MD simulation with the final state of the 2-ns TI/MD simulation for the correrspondiing perturbative transormation UB-ALT-P30 → ligand complexed with the hP2X7R.

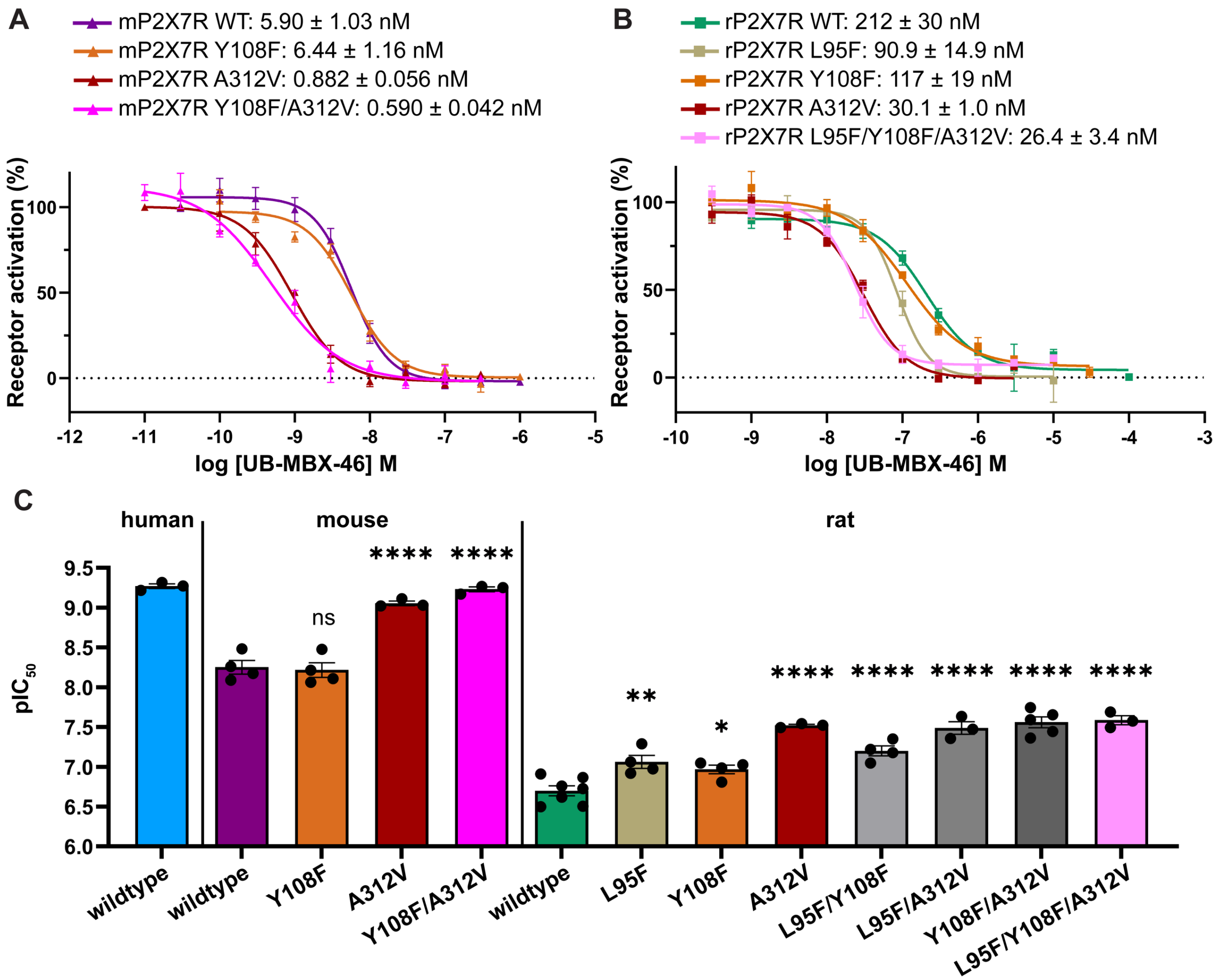

Supplementary Fig. 20: Mutagenesis of the ortholog-specific amino acid residues at positions 95, 108, and 312 for mouse, rat, and human P2X7R. (**A** and **B**) Concentration-dependent inhibition of BzATP-induced calcium influx (IC_{50} curves) by UB-MBX-46 at wild-type and mutant mouse (**A**) and rat (**B**) P2X7Rs expressed in HEK293T cells. (**C**) pIC_{50} values of UB-MBX-46 at wild-type and mutant human, mouse and rat P2X7Rs determined in BzATP-induced calcium influx assays using HEK293T cells expressing the respective P2X7R. Receptor activation was induced by an EC_{80} of the agonist BzATP (hP2X7R: 12.0 µM, mP2X7R: 17.3 µM, rP2X7R: 1.58 µM). Data represent mean ± SEM (hP2X7R wildtype: n=3, mP2X7R wildtype: n=4, mP2X7R Y108F: n=4, mP2X7R A312V: n=3, mP2X7R Y108F/A312V: n=3, rP2X7R wildtype: n=7, rP2X7R L95F: n=4, rP2X7R Y108F: n=4, rP2X7R A312V: n=3, rP2X7R L95F/Y108F: n=4, rP2X7R L95F/A312V: n=3, rP2X7R Y108F/A312V: n=5, rP2X7R L95F/Y108F/A312V: n=3). Statistical analysis of the pIC_{50} values of mutants compared to the respective wildtype receptor was performed using ANOVA (one-way, Dunnett post hoc); ns = not significant ($p > 0.05$), * $p \leq 0.05$, ** $p \leq 0.01$, **** $p \leq 0.0001$.

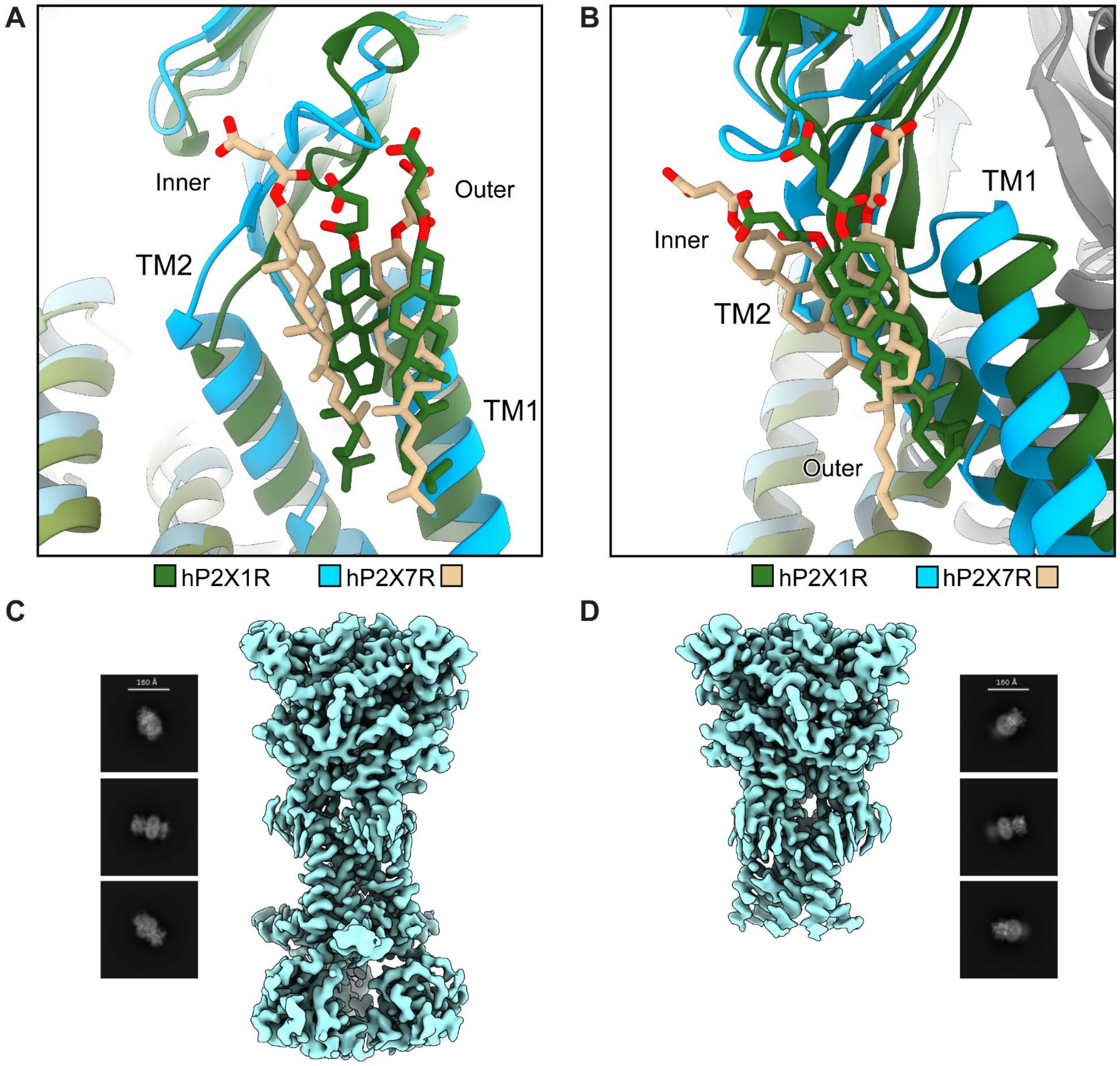

Supplementary Fig. 21: The cytoplasmic domain of the hP2X7R is stabilized by cholesterol. (**A and B**) Aligned structures of the hP2X1R and the hP2X7R highlighting the different positions of CHS molecules within their respective transmembrane domains. (**A**) Same view as Fig. 1C highlighting the position of the inner CHS molecules in the hP2X1R (receptor in green and CHS molecules in green) and the hP2X7R (receptor in blue and CHS molecules in tan). (**B**) Same view as Fig. 1D highlighting the position of the outer CHS molecules in the hP2X1R and the hP2X7R. Structures were aligned within ChimeraX[18]. (**C and D**) Cryo-EM processing of the hP2X7R in the apo closed and antagonist-bound inhibited states was able to classify out reconstructions with a stable (**C**) or flexible (**D**) cytoplasmic domain which includes the cytoplasmic cap, C-cys anchor, and cytoplasmic ballast. 2D projections from each reconstruction are shown next to the map to highlight a lack of ordered cryo-EM density for the ballast-less reconstruction. Reconstructions with or without the cytoplasmic domain contain cryo-EM densities for CHS molecules.

Supplementary Table 1: Cryo-EM collection, refinement, and validation statistics.

	Apo hP2X7R (EMD-47490) (PDB: 9E3M)	ATP hP2X7R (EMD-47491) (PDB: 9E3N)	UB-ALT-P30 hP2X7R (EMD-47492 (PDB: 9E3O)	UB-MBX-46 hP2X7R (EMDB-47493) (PDB: 9E3P)	Apo mP2X7R (EMD-47494) (PDB: 9E3Q)
**Data collection and processing**					
Magnification (kx)	130	37	130	130	130
Voltage (kV)	300	300	300	300	300
Electron exposure (e–/Å)	42	45	43	42	45
Movie frames	50	48	48	44	50
Defocus range (µm)	-0.8 to -1.4	-1.0 to -1.5	-0.9 to -1.5	-0.9 to -1.4	-0.9 to -1.5
Pixel size (Å/pixel)	0.648 (0.324 super-res)	0.623 (0.3115 super-res)	0.6507 (0.32535 super-res)	0.6483 (0.3242 super-res)	0.647 (0.3235 super-res)
Symmetry imposed	C3	C3	C3	C3	C3
Initial micrographs (no.)	8,314	8,721	14,463	24,191	15,909
Final micrographs used (no.)	7,891	7,396	13,586	22,699	13,524
Initial particle images (no.)	1,998,579	1,003,740	1,918,629	2,097,300	1,740,631
Final particle images (no.)	187,914	156,509	194,103	190,425	370,251
Map resolution (Å)	2.48	2.95	2.76	2.53	2.53
FSC threshold	(0.143)	(0.143)	(0.143)	(0.143)	(0.143)
Map resolution range (Å)	1.4 to 7.0	1.8 to 36	1.4 to 43	2.2 to 37	1.5 to 8.1
**Refinement**					
Initial model used (PDB code)	8TR5	6U9W	8TR5	8TR5	8TR5
Model resolution (Å)	2.45	2.91	2.69	2.50	2.49
FSC threshold	(0.143)	(0.143)	(0.143)	(0.143)	(0.143)
Map sharpening *B* factor ($Å^2$)	76.9	97.8	81.9	75.3	84.7
**Model composition**					
Non-hydrogen atoms	14,153	13,616	14,013	14,353	13,622
Protein Residues	1,635	1,623	1,623	1,632	1,620
Ligands	34	27	30	40	16
Waters	121	68	177	273	193
B factors ($Å^2$)					
Protein	17.8/118/49.8	8.03/106/58.7	6.0/63.8/31.4	9.80/68.4/33.0	12.5/90.4/39.4
Ligand	30.0/113/41.2	27.6/131/65.2	14.2/72.6/24.1	13.4/74.4/39.7	23.4/88.8/35.
Nucleotide	66.9/66.9/66.9	90.0/91.8/90.6	47.3/47.3/47.3	39.9/41.1/40.7	37.5/37.5/37.5
Water	15.5/64.1/26.5	17.4/51.1/35/7	5.5/46.8/14.7	8.44/33.8/19.2	11.6/44.2/23.0
R.m.s. deviations					
Bond lengths (Å)	0.008 (0)	0.007 (0)	0.009 (0)	0.007 (0)	0.004 (0)
Bond angles (°)	0.762 (0)	0.921 (0)	0.746 (0)	0.782 (0)	0.600 (0)
Validation					
MolProbity score	1.14	1.26	1.22	1.05	1.27
Clash score	3.50	4.90	4.37	2.66	5.09
Poor rotamers (%)	0.00	0	0	0.00	0.00
Ramachandran plot					
Favored (%)	98.29	98.09	98.27	98.85	98.85
Allowed (%)	1.71	1.91	1.73	1.15	1.15
Disallowed (%)	0	0	0	0	0

Synthesis and characterization methods

Chemistry. General methods

400 MHz ^{1}H NMR and 100.6 MHz ^{13}C NMR spectra were recorded on a Varian Mercury 400 or a Bruker 400. The chemical shifts are reported in ppm (δ scale) relative to internal tetramethylsilane, or to solvent peak, and coupling constants are reported in Hertz (Hz). Assignments given for the NMR spectra of the new compounds have been carried out based on homocorrelation $^{1}H/^{1}H$ (COSY) and/or heterocorrelation $^{1}H/^{13}C$ (HSQC) experiments. The used abbreviations were: s, singlet; d, doublet; m, multiplet; cs, complex signal; broad s, broad singlet, or combinations thereof. IR spectra were run on a FTIR Perkin-Elmer Spectrum RX I or a Perkin-Elmer Spectrum TWO spectrophotometers, using sodium chloride (NaCl) pellets or attenuated total reflectance (ATR) techniques. Absorption values are expressed as wavenumbers (cm^{-1}); only significant absorption bands are given. Column chromatography was performed on silica gel 60 Å (Sigma Aldrich, 40 - 63 μm, 230-400 mesh) or with a CombiFlash Rf 150 Teledine ISCO provided with a UV-Vis detector. Thin Layer Chromatography (TLC) was performed with aluminium-backed sheets with silica gel 60 F254 (Merck, ref 1.05554), and spots were visualized with UV light, 1% aqueous solution of $KMnO_4$, iodine or ninhydrin. Melting points were determined in open capillary tubes with an MFB 59510M Gallenkamp melting point apparatus. Accurate mass spectra were recorded with ESI techniques on a Hewlett-Packard 5988a LC/MSD-TOF instrument or a Thermo LTQ Orbitrap Velos instrument at *Unitat d'Espectrometria de Masses de Caracterització Molecular dels Centres Científics i Tecnològics de la Universitat de Barcelona* (CCiTUB). The elemental analyses were carried out in a Flash 1112 series Thermofinnigan elemental microanalyzer (A5) to determine C, H, and N at the *Servei de Microanàlisis* of IIQAB (CSIC) of Barcelona. The analytical samples of all the new compounds possessed purity ≥95% as evidenced by their elemental analyses or their HPLC-UV. HPLC-UV were determined with a HPLC Agilent 1260 Infinity II LC/MSD coupled to a photodiode array and mass spectrometer. Samples (5 μL, 0.5 mg/mL) in a 1:1 mixture of water with 0.05% formic acid (A) and acetonitrile with 0.05% formic acid (B) were injected using an Agilent Poroshell 120 EC-C18 (2.7 μm, 50 mm × 4.6 mm) column at 40 °C. The mobile phase was a mixture of A and B, with a flow 0.6 mL/min, using the following gradients: from 95% A–5% B to 100% B in 3 min; 100% B for 3 min; from 100% B to 95% A–5% B in 1 min; and 95% A–5% B for 3 min. Purity is given as % of absorbance at 254 nm.

General procedure A for the synthesis of the acyl chlorides

To a solution of the carboxylic acid (0.50 mmol, 1 eq) in thionyl chloride (13.5 mmol, 27 eq) at room temperature a drop of DMF is added and the solution is refluxed for 2 h in a round bottom flask provided with a tube of anhydrous $CaCl_2$. Then, the excess of thionyl chloride is removed under vacuum. Toluene (5 mL) is added to the residue, and the remaining thionyl chloride is azeotropically removed under vacuum (twice), to give the acyl chloride as a waxy solid in quantitative yield.

General procedure B for the synthesis of the carbohydrazides

Under nitrogen atmosphere, 2-chlorophenylhydrazine (0.56 mmol, 1.1 eq) is solved in anhydrous THF (2 mL) at room temperature. Anhydrous triethylamine (1.03 mmol, 2 eq) is added and the mixture is stirred for 10 minutes. Next, the corresponding acyl chloride (0.5

mmol, 1 eq) in anhydrous THF (7 mL) is added and the mixture is left stirring overnight at room temperature. The purification procedure is specified below for each compound.

UB-ALT-P30, N'-(2-chlorophenyl)adamantane-1-carbohydrazide
It was synthesized as previously described[3]. The NMR spectra of the obtained sample was in agreement with the reported data[3].

UB-MBX-46, N'-(2-chlorophenyl)-3,4,8,9-tetramethyltetracyclo[4.4.0.0^{3,9}.0^{4,8}]decane-1-carbohydrazide
3,4,8,9-Tetramethyltetracyclo[4.4.0.0^{3,9}.0^{4,8}]decane-1-carbonyl chloride (128 mg, 0.51 mmol) was obtained following the general procedure **A** from 3,4,8,9-tetramethyltetracyclo[4.4.0.0^{3,9}.0^{4,8}]decane-1-carboxylic acid (synthesized as previously described)[19] and, without further purification, it was reacted with 2-chlorophenylhydrazine hydrochloride (100 mg, 0.56 mmol) in the presence of anhydrous triethylamine (143 µL, 1.02 mmol), following the general procedure **B**. The obtained precipitate was filtered off and the organic layer was concentrated *in vacuo* and subsequently washed with 2 mL of a mixture of DCM/hexane (1/9), yielding the desired product as a beige solid (110 mg, 61% yield), mp 217 °C. IR (ATR) ν: 3858, 3615, 3331, 2941, 2334, 2192, 2131, 2030, 1653, 1590, 1489, 1319, 1271, 1160, 1105, 1029, 748 cm^{-1}. ^{1}H-NMR (400 MHz, $CDCl_3$) δ: 0.79 [dd, *J* = 11.6 Hz, *J'* = 2 Hz, 2H, 5(7)-H_a], 0.96 [d, *J* = 10.4 Hz, 2H, 2(10)-H_a], 0.968 [s, 6H, 3(9)-CH_3 or 4(8)-CH_3], 0.974 [s, 6H, 4(8)-CH_3 or 3(9)-CH_3], 1.84 [dd, *J* = 11.6 Hz, *J'* = 1.2 Hz, 2H, 5(7)-H_b] 1.99 [d, *J* = 11.2 Hz, 2H, 2(10)-H_b], 2.56 (broad s, 1H, 6-H), 6.49 (broad s, 1H, NH), 6.82 (m, 1H, 4'-H), 6.85 (dd, *J* = 8.0 Hz, *J'* = 1.2 Hz, 1H, 6'-H), 7.15 (m, 1H, 5'-H), 7.27 (dd, *J* = 8.0 Hz, *J'* = 1.6 Hz, 1H, 3'-H), 7.48 (s, 1H, NH). ^{13}C-NMR (100.6 MHz, $CDCl_3$) δ: 15.67 [CH_3, C3(9)-$\underline{C}H_3$ or C4(8)-$\underline{C}H_3$], 15.73 [CH_3, C4(8)-$\underline{C}H_3$ or C3(9)-$\underline{C}H_3$], 37.7 (CH, C6), 38.3 [CH_2, C5(7)], 41.2 [CH_2, C2(10)], 45.2 [C, C3(9) or C4(8)], 45.7 [C, C4(8) or C3(9)], 48.4 (C, C1), 113.7 (CH, C6'), 120.1 (C, C2'), 121.5 (CH, C4'), 127.8 (CH, C5'), 129.7 (CH, C3'), 144.4 (C, C1'), 177.0 (C, CO). HRMS-ESI+ *m/z* $[M+H]^+$ calcd for $[C_{21}H_{28}ClN_2O]^+$: 359.1885, found: 359.1892. Elemental analysis: Calculated for $C_{21}H_{27}ClN_2O$: C 70.28, H 7.58, N 7.81. Found: C 70.16, H 7.62, N 7.80.

UB-ALT-P36, N'-(2-chlorophenyl)-3,5-dimethyladamantane-1-carbohydrazide
3,5-Dimethyladamantane-1-carbonyl chloride (217 mg, 0.96 mmol) was obtained following the general procedure **A** from commercially available 3,5-dimethyladamantane-1-carboxylic acid and, without further purification, it was reacted with 2-chlorophenylhydrazine hydrochloride (189 mg, 1.05 mmol) in the presence of anhydrous triethylamine (267 µL, 1.92 mmol), following the general procedure **B**. The solved was evaporated under vacuum and DCM (10 mL) was added. The organic phase was washed with 2 N aqueous HCl solution (2 x 5 mL), 2 N aqueous NaOH solution (2 x 5 mL) and brine (2 x 5 mL), dried over anhydrous Na_2SO_4, filtered and concentrated to dryness under vacuum. An analytical sample of the resulting solid was obtained by crystallization from DCM/Pentane (133 mg, 40 % yield), mp 170-171 °C. IR (ATR) ν: 3355, 3279, 2947, 2894, 2841, 1651, 1593, 1493, 1469, 1447, 1376, 1358, 1290, 1230, 1190, 1157, 1140, 1116, 1047, 1033, 918, 882, 842, 742, 704, 648, 630, 593, 555 cm^{-1}. ^{1}H-NMR (400 MHz, $CDCl_3$) δ: 0.87 [s, 6H, 3(5)-$C\underline{H}_3$], 1.19 (m, 2H, 4-H_2), 1.38 [m, 4H, 6(10)-H_2], 1.54 [m, 4H, 2(9)-H_2], 1.76 (broad s, 2H, 8-H_2), 2.16 (m, 1H, 7-H), 6.44 (broad s, 1H, NH), 6.80-6.85 (cs, 2H, 4'-H

and 6'-H), 7.14 (m, 1H, 5'-H), 7.27 (d, *J* = 7.6 Hz, 1H, 3'-H), 7.48 (s, 1H, NH). ^{13}C-NMR (100.6 MHz, $CDCl_3$) δ: 29.3 (CH, C7), 30.5 [CH_3, C3(5)-$\underline{C}H_3$], 31.2 [C, C3(5)], 37.9 (CH_2, C8), 42.4 (C, C1), 42.8 [CH_2, C6(10)], 45.3 [CH_2, C2(9)], 50.7 (CH_2, C4), 113.6 (CH, C6'), 120.1 (C, C2'), 121.6 (CH, C4'), 127.7 (CH, C5'), 129.7 (CH, C3'), 144.3 (C, C1'), 177.3 (C, CO). HRMS-ESI+ *m/z* $[M+H]^+$ calcd for $[C_{19}H_{26}ClN_2O]^+$: 333.1728, found: 333.1736. Elemental analysis: Calculated for $C_{19}H_{25}ClN_2O$: C 68.56, H 7.57, N 8.42. Found: C 68.50, H 7.46, N 8.30.

UB-ALT-P37, N'-(2-chlorophenyl)-3,5,7-trimethyladamantane-1-carbohydrazide
3,5,7-Trimethyladamantane-1-carbonyl chloride (215 mg, 0.46 mmol) was obtained following the general procedure **A** from commercially available 3,5,7-trimethyladamantane-1-carboxylic acid and, without further purification, it was reacted with 2-chlorophenylhydrazine hydrochloride (94 mg, 0.52 mmol) in the presence of anhydrous triethylamine (133 µL, 0.96 mmol), following the general procedure **B**. The solved was evaporated under vacuum and DCM (10 mL) was added. The organic phase was washed with 2 N aqueous HCl solution (2 x 5 mL), 2 N aqueous NaOH solution (2 x 5 mL) and brine (2 x 5 mL), dried over anhydrous Na_2SO_4, filtered and concentrated to dryness under vacuum. An analytical sample of the isolated solid was obtained by crystallization from DCM/Pentane (112 mg, 67 % yield), mp 170-172 °C. IR (ATR) ν: 3277, 2943, 2890, 2836, 1661, 1597, 1544, 1475, 1454, 1410, 1373, 1355, 1292, 1230, 1208, 1156, 1139, 1116, 1099, 1067, 1044, 1035, 886, 851, 829, 774, 733, 667, 548 cm^{-1}. ^{1}H-NMR (400 MHz, $CDCl_3$) δ: 0.88 [s, 9H, 3(5,7)-$C\underline{H}_3$], 1.13 [m, 6H, 4(6,10)-H_2] 1.48 [broad s, 6H, 2(8,9)-H_2], 6.43 (broad s, 1H, NH), 6.80-6.85 (cs, 2H, 4'-H and 6'-H), 7.15 (m, 1H, 5'-H), 7.27 (d, *J* = 8.0 Hz, 1H, 3'-H), 7.46 (s, 1H, NH). ^{13}C-NMR (100.6 MHz, $CDCl_3$) δ: 30.2 [CH_3, C3(5,7)-$\underline{C}H_3$], 32.0 [C, C3(5,7)], 43.3 (C, C1), 44.7 [CH_2, C2(8,9)], 50.1 [CH_2, C4(6,10)], 113.6 (CH, C6'), 120.2 (C, C2'), 121.6 (CH, C4'), 127.7 (CH, C5'), 129.7 (CH, C3'), 144.3 (C, C1'), 177.2 (C, CO). HRMS-ESI+ *m/z* $[M+H]^+$ calcd for $[C_{20}H_{28}ClN_2O]^+$: 347.1885, found: 347.1894. Elemental analysis: Calculated for $C_{20}H_{27}ClN_2O$: C 69.25, H 7.85, N 8.08. Found: C 69.18, H 7.85, N 7.98.

UB-ALT-P38, N'-(2-chlorophenyl)cubane-1-carbohydrazide
Cubane-1-carbonyl chloride (55 mg, 0.33 mmol) was obtained following the general procedure **A** from commercially available cubane-1-carboxylic acid and, without further purification, it was reacted with 2-chlorophenylhydrazine hydrochloride (65 mg, 0.36 mmol) in the presence of anhydrous triethylamine (87 µL, 0.66 mmol), following the general procedure **B**. The solved was evaporated under vacuum and DCM (10 mL) was added. The organic phase was washed with 2 N aqueous HCl solution (2 x 5 mL), 2 N aqueous NaOH solution (2 x 5 mL) and brine (2 x 5 mL), dried over anhydrous Na_2SO_4, filtered and concentrated to dryness under vacuum. An analytical sample of the isolated solid was obtained by crystallization from DCM/Pentane (61 mg, 68 % yield), mp 189-190 °C. IR (ATR) ν: 3315, 3191, 3060, 2993, 2969, 1625, 1598, 1544, 1481, 1427, 1333, 1291, 1253, 1230, 1217, 1167, 1148, 1124, 1104, 1086, 1058, 1035, 972, 939, 885, 869, 839, 825, 743, 716, 693, 665, 645, 629, 583 cm^{-1}. ^{1}H-NMR (400 MHz, $CDCl_3$) δ: mayor rotamer, 4.04-4.09 [cs, 4H, 3(5,7)-H and 4-H], 4.31 [m, 3H, 2(6,8)], 6.43 (broad s, 1H, NH), 6.80-6.90 (cs, 2H, 4'-H and 6'-H), 7.16 (m, 1H, 5'-H), 7.26-7.30 (cs, 2H, 3'-H and NH): minor rotamer, 3.92 [cs, 4H, 3(5,7)-H and 4-H], 4.16 [m, 3H, 2(6,8)], 6.25 (broad s, 1H, NH), 6.80-6.90 (m, 1H, 4'-H; overlapped with the other rotamer), 6.96 (d, *J* = 8.0 Hz, 1H, 6'-H), 7.22 (m, 1H, 5'-H), 7.26-7.30 (cs, 2H, 3'-H and NH; overlapped with the other rotamer). ^{13}C-NMR

(100.6 MHz, $CDCl_3$) δ: mayor rotamer, 45.3 [CH, C3(5,7)], 48.1 (CH, C4), 49.6 [(CH, C2(6,8)], 56.2 (C, C1), 113.7 (CH, C6’), 120.0 (C, C2’), 121.6 (CH, C4’), 127.7 (CH, C5’), 129.6 (CH, C3’), 144.0 (C, C1’), 171.9 (C, CO); minor rotamer, 45.4 [CH, C3(5,7)], 47.3 (CH, C4), 49.7 [(CH, C2(6,8)], 57.0 (C, C1), 112.6 (CH, C6’), 118.3 (C, C2’), 121.3 (CH, C4’), 128.1 (CH, C5’), 129.7 (CH, C3’), 143.4 (C, C1’), 177.5 (C, CO). HRMS-ESI+ *m/z* $[M+H]^+$ calcd for $[C_{15}H_{14}ClN_2O]^+$: 273.0789, found: 273.0793. Elemental analysis: Calculated for $C_{15}H_{13}ClN_2O \cdot 0.05CH_2Cl_2$: C 65.26, H 4.77, N 10.11. Found: C 65.49, H 4.80, N 9.90. HPLC-UV purity: 96.6%

Detailed molecular dynamics simulations methods

Ligands Preparation

Molecules UB-MBX-46, UB-ALT-P36, UB-ALT-P37, UB-ALT-P38, UB-MBX-P1, and UB-MBX-P2 were generated by building the corresponding cage alkyls on the coordinates of the UB-ALT-P30 in complex with the hP2X7R using the Maestro interface (Schrödinger Release 2021-2: Maestro, Schrödinger, LLC, New York, NY, 2021). Subsequently, a minimization procedure was applied to all atoms of the protein complexes using the OPLS-2005 force field to ensure the stability of the complexes[20-22].

Protein Preparation

The cryo-EM structures of the full-length wild-type hP2X7R in the apo closed state or full-length wild-type hP2X7R in complex with UB-ALT-P30 or UB-MBX-46 were utilized as starting models for MD simulations. In addition, models of the full-length wild-type hP2X7R in complex with UB-ALT-P36, UB-ALT-P37, UB-ALT-P38, UB-MBX-P1, or UB-MBX-P2 were prepared from the experimental structure of UB-ALT-P30 bound to the full-length wild-type hP2X7R. Both the N- and C-termini of the receptor were capped with acetyl and methylamino groups, respectively. The protein structure was optimized using the protein preparation module of Maestro software (Protein Preparation Wizard 2015-2; Schrödinger Release 2021-2: Maestro, Schrödinger, LLC, New York, NY, 2021)[23]. In this process, the bond orders and disulfide bonds were assigned, and missing hydrogen atoms and loops were added using Prime within Maestro[24,25]. As post preparation, all hydrogens in the protein complex were minimized employing the AMBER* force field via Maestro/Macromodel, with a distance-dependent dielectric constant set at 4.0[26,27]. The molecular mechanics minimizations were conducted using a conjugate gradient method, setting a convergence criterion threshold at 0.0001 kJ $Å^{-1}$ mol^{-1}. The ionization states of the compounds at pH 7.5 were confirmed based on the Epik program[28]. The protein complex was subjected in an all-atom minimization using the OPLS2005 force field with heavy atom RMSD values constrained to 0.30 Å (Schrödinger Release 2021-2: Maestro, Schrödinger, LLC, New York, 2020)[29].

System setup for MD simulations

Each model, prepared as mentioned above, was inserted in a pre-equilibrated hydrated POPC bilayer expanding 30 Å from the furthermost vertex of the protein to the edge of the simulation orthorhombic box in all axes. The protein was positioned with respect to the membrane plane (x,y plane) as suggested by the server "Orientations of Proteins in Membranes (OPM)" database using the System setup utility in Schrodinger Maestro software (Schrödinger Release 2021-2: Maestro, Schrödinger, LLC, New York, 2021) using the System Builder Wizard in Maestro software (Schrödinger Release 2021-4: Desmond Molecular Dynamics System, D. E. Shaw Research, New York, NY, 2021)[30]. Using the same utility, water molecules were added while sodium and chloride ions were added randomly in the water phase to neutralize the system and reach the experimental salt concentration of 0.150 M NaCl[31]. The resulting lipid buffer contained approximately ~ 479,000 atoms, consisting of 584 POPC lipids and ~ 123,500 water molecules. The dimensions of the simulation box were 150×148×226 $Å^3$. Periodic boundary conditions were applied. We used the LEaP, the main program for preparing simulations in Amber Software, antechamber and Parmchk2 of AmberTools22 to assign the

ff19sb parameters to model the protein, the lipid21 force field parameters to model the POPC lipids, the GAFF2 parameters to model the ligand, and the TIP3P model for waters and ions[32-39]. Partial charges for ligands were obtained using RESP fitting of the electrostatic potentials calculated with Gaussian03 at the Hartree-Fock (HF)/6-31G* level of theory and the antechamber of AmberTools22[39-41].

Simulations protocol

For each system, we executed an equilibration phase consisting of seven steps, starting with an energy minimization step. The first step includes energy minimization. Thus, systems were equilibrated by 5000 steps of energy minimization (1500 steps with the steepest descent algorithm and 3500 cycles with the conjugate gradient algorithm) in the presence of a harmonic restraint with a force constant of 10 kcal mol^{-1} Å^{-2} and 5 kcal mol^{-1} Å^{-2} on the heavy atoms of protein, ligand and lipid head groups. In the next two constant volume equilibration (*NVT*) steps and the three constant pressure equilibration (*NPT*) simulation steps the temperature was raised from 0 K to 310 K and a gradual reduction in position restraints was implemented to maintain protein-ligand stability and ensure optimal lipid packing while heating was also applied. In the first step an 125 ps *NVT* simulation was applied at 0 K in the presence of a harmonic restraint with a force constant of 10 kcal mol^{-1} Å^{-2} on all protein and ligand heavy atoms and 5 kcal mol^{-1} Å^{-2} on lipid head groups and in the second step an 125 ps *NVT* simulation was performed at 0 K while the force constant was reduced to 5 and 2.5 kcal mol^{-1} Å^{-2}, respectively.

In the third step, the temperature was raised to 310 K in a *NPTγ* (with $\gamma = 10$ dyn cm^{1}) simulation of 125 ps length in the presence of restraint with a force constant of 2.5 kcal mol^{-1} Å^{-2} on protein, ligand heavy atoms and 1.0 kcal mol^{-1} Å^{-2} on lipid head groups. For the remaining three 500 ps NPT simulation equilibration steps the applied restraints correspond to a force constant 1.0 and 0.5 kcal mol^{-1} Å^{-2} then 0.5 and 0.1 kcal mol^{-1} Å^{-2} and finally 0.1 and 0 kcal mol^{-1} Å^{-2}, respectively. In the *NPTγ* simulations a surface tension 0 dyn cm^{1} was implemented on *x*-*y* plane which gives pure semi-isotropic conditions. *NVT* MD simulation steps were performed with Langevin thermostat (dynamics) for temperature control as implemented in Amber22 software, employing a Langevin collision frequency of 2.0 ps and a friction coefficient constant at 1 ps^{-1} in the *NPTγ* MD simulation steps for the pressure control the Berendsen barostat with a target pressure of 1 bar and a 2 ps pressure relaxation time for the pressure control was used and the Langevin thermostat with a collision frequency of 2 ps^{-1} was applied[38,42-44]. The temperature of 310 K was used in MD simulations to ensure that the membrane state is above the main phase transition temperature of 271 K for POPC bilayers[45].

Bonds involving hydrogen atoms were constrained by the SHAKE algorithm and a time step of 1 fs was used for the integration of the equations of motion for the first 2 NVT and the first NPT equilibration steps and for the rest of the NPT steps the time step was set at 2 fs with the leapfrog Verlet integrator[46,47]. Long range electrostatics were calculated using Particle-mesh Ewald summation (PME), with a 1 Å grid, and short-range non-bonding interactions were truncated at 12 Å with a continuum model long range correction applied for energy and pressure. The equilibration phase was followed by production MD simulation for 500 ns or 1 μs for the apo full-length wild-type hP2X7R or its complexes with UB-ALT-P30 and UB-MBX-46,

respectively, using the same protocol as in the final equilibration step. Snapshots were recorded every 100 ps during the production phase. Within this simulation time, the RMSD (Cα) reached a plateau, and the systems were considered equilibrated and suitable for statistical analysis. Three MD simulation repeats were performed for each complex using the same starting structure and applying randomized velocities.

Short duration MD simulations (50 ns) were also performed for the complexes of UB-MBX-46, UB-ALT-P36, UB-ALT-P37, UB-ALT-P38, UB-MBX-P1, UB-MBX-P2 bound to the hP2X7R to compare structures with the last snapshots from the TI/MD simulations.

Particle Mesh Ewald Molecular Dynamics (pmemd) is the primary engine for running MD simulations with Amber22 software and the energy minimization step was performed using the Central Processing Unit (CPU) of the workstations by the implementation of pmemd[38,39]. The rest of the equilibration steps including the unrestraint production were run with Amber22 software on RTX 4090 GPUs in lab workstations using pmemd.CUDA algorithm[38,39,48]. The pmemd.CUDA executable provides the ability to use NVIDIA GPUs to run the MD simulations.

Analysis of MD simulations
The visualization of the MD simulation trajectories was performed using VMD[49]. The analysis of all the MD simulations trajectories was performed by *ptraj* and *cpptraj* of AmberTools22[39,50]. For hydrogen bond interactions distance = 2.5 Å between donor and acceptor heavy atoms, and an angle ≥120° between donor-hydrogen-acceptor atoms and ≥ 90° between hydrogen-acceptor-bonded atoms were considered. Non-specific hydrophobic contacts were measured if the residue fell within 4.0 Å from a ligand's aromatic or aliphatic carbon, while π-π interactions were measured if two aromatic groups are stacked face-to-face or face-to-edge. Water-mediated interactions were measured if the distance between donor and acceptor atoms is 2.7 Å, the angle between donor-hydrogen-acceptor atoms is ≥ 110° and the angle between hydrogen-acceptor-bonded atoms is ≥ 80°.

Calculation of RBFEs
Perturbative free energy calculations of the relative binding free energies for congeneric series of ligands based on experimental structures of membrane proteins have been successfully applied for retrospective and prospective predictions in drug design[51-53]. For the TI/MD simulations, binding poses of UB-ALT-P36, UB-ALT-P37, UB-ALT-P38, UB-MBX-P1, UB-MBX-P2, UB-MBX-46 aligned with the experimental structure of UB-ALT-P30 in complex with the hP2X7R were used as starting structures for the alchemical calculations described in Table 1. TI/MD calculations were also performed for the ligands in solution.

The setup procedure was the same as previously reported for the MD simulations with Amber22 software[38]. The bond constraint SHAKE algorithm was disabled for TI mutations in AMBER GPU-TI module pmemdGTI, and therefore a time step of 1 fs was used for all MD simulations[46,54]. Long range electrostatics were calculated using PME, with a 1 Å grid, and short-range non-bonding interactions were truncated at 12 Å with a continuum model long range correction applied for energy and pressure[55]. The ff19sb was used to model the protein, the

lipid21 force field to model the POPC lipids, the GAFF2 to model the ligand, and the TIP3P model for waters and ions[32-37].

Thus, initial geometries were minimized using 20,000 steps of steepest descent minimization at λ = 0.5. These minimized geometries were then used for simulations at all λ values. Eleven λ values were applied, equally spaced between 0.0 to 1.0 to collect $\partial V/\partial\lambda$ data (see Supplementary Fig. 18). A 12-point Gaussian quadrature was used for the numerical integration of $\partial U/\partial\lambda$ to obtain all necessary ΔA values. The system was heated to 310 K and 500 ps MD simulation in the *NPT* ensemble was performed using the Langevin thermostat (dynamics) for temperature control, as implemented in Amber22 software employing a Langevin collision frequency of 2.0 ps^{-1} in the presence of harmonic restraint with force constant 10 kcal mol^{-1} $Å^{-2}$ on all membrane, protein, and ligand atoms[38,39]. The Berendsen barostat was used to adjust the density over the 500 ps MD simulations at constant pressure (*NPTγ*) (with γ = 10 dyn cm^{1}), with a target pressure of 1 bar and a 2 ps pressure relaxation time[42]. After the 500 ps of *NPT* MD simulation was followed by 2 ns *NVT* production simulation without restraints using the Berendsen thermostat with a coupling constant of 2 ps. The temperature of 310 K was used in MD simulations to ensure that the membrane state is above the main phase transition temperature of 271 K for POPC bilayers[45]. Energies were recorded every 1 ps, and coordinates were saved every 10 ps. Production simulations recalculated the potential energy at each λ value every 1 ps for later analysis with MBAR[55,56].

For each alchemical calculation was applied dual topology and the 1-step protocol was performed which includes disappearing one ligand and appearing the other ligand simultaneously, and the electrostatic and van der Waals interactions are scaled simultaneously using softcore potentials from real atoms that are transformed into dummy atoms[57,58]. Two repeats were performed for the TI/MD calculation for each alchemical transformation and the resultant $\Delta\Delta G_{b,\ TI/MD}$ values are shown in Table 1. The calculation affords the Helmholtz free energy $\Delta\Delta A_b$ values and not the Gibbs free energy $\Delta\Delta G_b$ values. Gibbs free energy is related to the Helmholtz free energy according to the equation: $G=A+PV$. Assuming the volume change upon binding to be negligible, which is often the case at 1 atm due to the incompressibility of the system, then the Gibbs free energy ΔG_{bind} is approximately equal to the Helmholtz free energy ΔA_{bind}[57]. The deviation of $\Delta\Delta A_{bind}$ from $\Delta\Delta G_{bind}$ is further reduced since we calculated differences in ΔA_{bind} values. Therefore $\Delta\Delta A_{bind}$ should be considered that is almost indistinguishable from $\Delta\Delta G_{bind}$).

The $\Delta V/\Delta\lambda$ convergence plots for each of the TI/MD simulations are shown in Supplementary Fig. 18. Additionally, the final state of the alchemical calculation UB-ALT-P30 → ligand in Table 1 complexed with hP2X7R, i.e., the snapshot of the corresponding ligand as resulted from the 2 ns-TI/MD simulation, was compared with the structure of this ligand in its complex with hP2X7R resulted from 50 ns-MD simulations. This was performed to certify that the 2 ns-MD simulation for each λ-state during the TI/MD simulation was enough to converge to the same structure from the 50 ns-MD simulations. In Supplementary Fig. 19 are shown a selected image from superimposed structures of the final snapshots of UB-MBX-46 from the 2 ns-TI/MD simulation of

the perturbative transformation UB-ALT-P30 → UB-MBX-46 complexed with hP2X7R and from the 50 ns unrestrained *NPT*-MD simulation of UB-MBX-46 in complex with hP2X7R as well as the RMSD values in tabulated form of all the ligands in Table 1 after the described superposition.

Experimental relative binding free energies for the alchemical transformations from UB-ALTP-30 (ligand 0) to another ligand 1 were estimated using the experimental $\mathrm{pIC}_{50,0}$ / $\mathrm{pIC}_{50,1}$ values depicted in Table 1 to approximate $\mathrm{p}K_{\mathrm{d},0}$ / $\mathrm{p}K_{\mathrm{d},1}$ according to the following equation (T = 298 K)

$$\Delta G_{\mathrm{b},0\rightarrow 1,\mathrm{exp}} = -1.9872\, T\left(\mathrm{p}K_{\mathrm{d},0} - \mathrm{p}K_{\mathrm{d},1}\right) \sim\ \Delta G_{\mathrm{b},0\rightarrow 1,\mathrm{exp}} = -1.9872\, T\,(\mathrm{pIC}_{50,0} - \mathrm{pIC}_{50,1}) \qquad (1)$$

Calculation of RSFE

To calculate the relative solvation free energy (RSFE) the TI/MD simulations for the alchemical calculation **UB-ALT-P30 → UB-MBX-46** was performed in the water and gas phase. The simulation box extends 15×15×17.5 $Å^3$ from the furthermost vertex of the ligand to the edge of the simulation orthorhombic box in all axes containing 3889 waters. The same parameters and simulation protocol were used as described previously for the alchemical transformation calculations.

Supplemental References

1. Karasawa, A. & Kawate, T. Structural basis for subtype-specific inhibition of the P2X7 receptor. *Elife* **5**(2016).
2. Oken, A.C. et al. P2X7 receptors exhibit at least three modes of allosteric antagonism. *Science Advances* **10**, eado5084 (2024).
3. Nelson, D.W. et al. Structure−Activity Relationship Studies on N′ -Aryl Carbohydrazide P2X7 Antagonists. *Journal of Medicinal Chemistry* **51**, 3030-3034 (2008).
4. Kawate, T., Michel, J.C., Birdsong, W.T. & Gouaux, E. Crystal structure of the ATP-gated P2X(4) ion channel in the closed state. *Nature* **460**, 592-8 (2009).
5. McCarthy, A.E., Yoshioka, C. & Mansoor, S.E. Full-Length P2X(7) Structures Reveal How Palmitoylation Prevents Channel Desensitization. *Cell* **179**, 659-670 e13 (2019).
6. Punjani, A., Rubinstein, J.L., Fleet, D.J. & Brubaker, M.A. cryoSPARC: algorithms for rapid unsupervised cryo-EM structure determination. *Nature Methods* **14**, 290-296 (2017).
7. Punjani, A., Zhang, H. & Fleet, D.J. Non-uniform refinement: adaptive regularization improves single-particle cryo-EM reconstruction. *Nature Methods* **17**, 1214-1221 (2020).
8. Oken, A.C. et al. High-affinity agonism at the P2X7 receptor is mediated by three residues outside the orthosteric pocket. *Nature Communications* **15**, 6662 (2024).
9. Barton, G.J. ALSCRIPT: a tool to format multiple sequence alignments. *Protein Engineering, Design and Selection* **6**, 37-40 (1993).
10. Sievers, F. & Higgins, D.G. Clustal Omega for making accurate alignments of many protein sequences. *Protein Science* **27**, 135-145 (2018).
11. Bond, C.S. & Schuttelkopf, A.W. ALINE: a WYSIWYG protein-sequence alignment editor for publication-quality alignments. *Acta Crystallographica Section D, Biological Crystallography* **65**, 510-2 (2009).
12. Pravda, L. et al. MOLEonline: a web-based tool for analyzing channels, tunnels and pores (2018 update). *Nucleic Acids Research* **46**, W368-W373 (2018).
13. Hille, B. *Ion channels of excitable membranes*, xviii, 814 p. (Sinauer, Sunderland, Mass., 2001).
14. Li, M., Kawate, T., Silberberg, S.D. & Swartz, K.J. Pore-opening mechanism in trimeric P2X receptor channels. *Nat Commun* **1**, 44 (2010).
15. Kawate, T. P2X Receptor Activation. *Adv Exp Med Biol* **1051**, 55-69 (2017).
16. Laskowski, R.A. & Swindells, M.B. LigPlot+: Multiple Ligand–Protein Interaction Diagrams for Drug Discovery. *Journal of Chemical Information and Modeling* **51**, 2778-2786 (2011).
17. Barniol-Xicota, M. et al. Escape from adamantane: Scaffold optimization of novel P2X7 antagonists featuring complex polycycles. *Bioorganic & Medicinal Chemistry Letters* **27**, 759-763 (2017).
18. Meng, E.C. et al. UCSF ChimeraX: Tools for structure building and analysis. *Protein Science* **32**, e4792 (2023).

19. Leiva, R. et al. Pharmacological and Electrophysiological Characterization of Novel NMDA Receptor Antagonists. *ACS Chemical Neuroscience* **9**, 2722-2730 (2018).
20. Jorgensen, W.L., Maxwell, D.S. & Tirado-Rives, J. Development and Testing of the OPLS All-Atom Force Field on Conformational Energetics and Properties of Organic Liquids. *Journal of the American Chemical Society* **118**, 11225-11236 (1996).
21. Jorgensen, W.L. & Tirado-Rives, J. The OPLS [optimized potentials for liquid simulations] potential functions for proteins, energy minimizations for crystals of cyclic peptides and crambin. *Journal of the American Chemical Society* **110**, 1657-1666 (1988).
22. Shivakumar, D. et al. Prediction of Absolute Solvation Free Energies using Molecular Dynamics Free Energy Perturbation and the OPLS Force Field. *Journal of Chemical Theory and Computation* **6**, 1509-1519 (2010).
23. Madhavi Sastry, G., Adzhigirey, M., Day, T., Annabhimoju, R. & Sherman, W. Protein and ligand preparation: parameters, protocols, and influence on virtual screening enrichments. *Journal of Computer-Aided Molecular Design* **27**, 221-234 (2013).
24. Jacobson, M.P., Friesner, R.A., Xiang, Z. & Honig, B. On the Role of the Crystal Environment in Determining Protein Side-chain Conformations. *Journal of Molecular Biology* **320**, 597-608 (2002).
25. Jacobson, M.P. et al. A hierarchical approach to all-atom protein loop prediction. *Proteins: Structure, Function, and Bioinformatics* **55**, 351-367 (2004).
26. Mohamadi, F. et al. Macromodel—an integrated software system for modeling organic and bioorganic molecules using molecular mechanics. *Journal of Computational Chemistry* **11**, 440-467 (1990).
27. Watts, K.S., Dalal, P., Tebben, A.J., Cheney, D.L. & Shelley, J.C. Macrocycle Conformational Sampling with MacroModel. *Journal of Chemical Information and Modeling* **54**, 2680-2696 (2014).
28. Shelley, J.C. et al. Epik: a software program for pKaprediction and protonation state generation for drug-like molecules. *Journal of Computer-Aided Molecular Design* **21**, 681-691 (2007).
29. Kaminski, G.A., Friesner, R.A., Tirado-Rives, J. & Jorgensen, W.L. Evaluation and Reparametrization of the OPLS-AA Force Field for Proteins via Comparison with Accurate Quantum Chemical Calculations on Peptides. *The Journal of Physical Chemistry B* **105**, 6474-6487 (2001).
30. Lomize, M.A., Pogozheva, I.D., Joo, H., Mosberg, H.I. & Lomize, A.L. OPM database and PPM web server: resources for positioning of proteins in membranes. *Nucleic Acids Research* **40**, D370-D376 (2012).
31. Jorgensen, W.L., Chandrasekhar, J., Madura, J.D., Impey, R.W. & Klein, M.L. Comparison of simple potential functions for simulating liquid water. *The Journal of Chemical Physics* **79**, 926-935 (1983).
32. Dickson, C.J., Walker, R.C. & Gould, I.R. Lipid21: Complex Lipid Membrane Simulations with AMBER. *Journal of Chemical Theory and Computation* **18**, 1726-1736 (2022).

33. He, X., Man, V.H., Yang, W., Lee, T.-S. & Wang, J. A fast and high-quality charge model for the next generation general AMBER force field. *The Journal of Chemical Physics* **153**, 114502 (2020).
34. Joung, I.S. & Cheatham, T.E., III. Determination of Alkali and Halide Monovalent Ion Parameters for Use in Explicitly Solvated Biomolecular Simulations. *The Journal of Physical Chemistry B* **112**, 9020-9041 (2008).
35. Sengupta, A., Li, Z., Song, L.F., Li, P. & Merz, K.M., Jr. Parameterization of Monovalent Ions for the OPC3, OPC, TIP3P-FB, and TIP4P-FB Water Models. *Journal of Chemical Information and Modeling* **61**, 869-880 (2021).
36. Li, P., Song, L.F. & Merz, K.M., Jr. Systematic Parameterization of Monovalent Ions Employing the Nonbonded Model. *Journal of Chemical Theory and Computation* **11**, 1645-1657 (2015).
37. Tian, C. et al. ff19SB: Amino-Acid-Specific Protein Backbone Parameters Trained against Quantum Mechanics Energy Surfaces in Solution. *Journal of Chemical Theory and Computation* **16**, 528-552 (2020).
38. Case, D.A. et al. The Amber biomolecular simulation programs. *Journal of Computational Chemistry* **26**, 1668-1688 (2005).
39. Case, D.A. et al. AmberTools. *Journal of Chemical Information and Modeling* **63**, 6183-6191 (2023).
40. Bayly, C.I., Cieplak, P., Cornell, W. & Kollman, P.A. A well-behaved electrostatic potential based method using charge restraints for deriving atomic charges: the RESP model. *Journal of physical chemistry (1952)* **97**, 10269-10280 (1993).
41. Davidson, E.R. & Feller, D. Basis set selection for molecular calculations. *Chemical Reviews* **86**, 681-696 (1986).
42. Berendsen, H.J.C., Postma, J.P.M., van Gunsteren, W.F., DiNola, A. & Haak, J.R. Molecular dynamics with coupling to an external bath. *The Journal of Chemical Physics* **81**, 3684-3690 (1984).
43. Izaguirre, J.A., Reich, S. & Skeel, R.D. Longer time steps for molecular dynamics. *The Journal of Chemical Physics* **110**, 9853-9864 (1999).
44. Izaguirre, J.A., Catarello, D.P., Wozniak, J.M. & Skeel, R.D. Langevin stabilization of molecular dynamics. *The Journal of Chemical Physics* **114**, 2090-2098 (2001).
45. Koynova, R. & Caffrey, M. Phases and phase transitions of the phosphatidylcholines. *Biochimica et Biophysica Acta (BBA) - Reviews on Biomembranes* **1376**, 91-145 (1998).
46. Ryckaert, J.-P., Ciccotti, G. & Berendsen, H.J.C. Numerical integration of the cartesian equations of motion of a system with constraints: molecular dynamics of n-alkanes. *Journal of Computational Physics* **23**, 327-341 (1977).
47. Verlet, L. Computer "Experiments" on Classical Fluids. I. Thermodynamical Properties of Lennard-Jones Molecules. *Physical Review* **159**, 98-103 (1967).
48. Salomon-Ferrer, R., Götz, A.W., Poole, D., Le Grand, S. & Walker, R.C. Routine Microsecond Molecular Dynamics Simulations with AMBER on GPUs. 2. Explicit Solvent Particle Mesh Ewald. *Journal of Chemical Theory and Computation* **9**, 3878-3888 (2013).

49. Humphrey, W., Dalke, A. & Schulten, K. VMD: Visual molecular dynamics. *Journal of Molecular Graphics* **14**, 33-38 (1996).
50. Roe, D.R. & Cheatham, T.E., III. PTRAJ and CPPTRAJ: Software for Processing and Analysis of Molecular Dynamics Trajectory Data. *Journal of Chemical Theory and Computation* **9**, 3084-3095 (2013).
51. Stampelou, M. et al. Dual A1/A3 Adenosine Receptor Antagonists: Binding Kinetics and Structure–Activity Relationship Studies Using Mutagenesis and Alchemical Binding Free Energy Calculations. *Journal of Medicinal Chemistry* **65**, 13305-13327 (2022).
52. Lenselink, E.B. et al. Predicting Binding Affinities for GPCR Ligands Using Free-Energy Perturbation. *ACS Omega* **1**, 293-304 (2016).
53. Matricon, P. et al. Fragment-based design of selective GPCR ligands guided by free energy simulations. *Chemical Communications* **57**, 12305-12308 (2021).
54. Lee, T.-S., Hu, Y., Sherborne, B., Guo, Z. & York, D.M. Toward Fast and Accurate Binding Affinity Prediction with pmemdGTI: An Efficient Implementation of GPU-Accelerated Thermodynamic Integration. *Journal of Chemical Theory and Computation* **13**, 3077-3084 (2017).
55. Essmann, U. et al. A smooth particle mesh Ewald method. *The Journal of Chemical Physics* **103**, 8577-8593 (1995).
56. Shirts, M.R. & Pande, V.S. Comparison of efficiency and bias of free energies computed by exponential averaging, the Bennett acceptance ratio, and thermodynamic integration. *The Journal of Chemical Physics* **122**, 144107 (2005).
57. Mey, A.S.J.S. et al. Best Practices for Alchemical Free Energy Calculations [Article v1.0]. *Living Journal of Computational Molecular Science* **2**, 18378 (2020).
58. Song, L.F., Lee, T.-S., Zhu, C., York, D.M. & Merz, K.M., Jr. Using AMBER18 for relative free energy calculations. *Journal of Chemical Information and Modeling* **59**, 3128-3135 (2019).
